# Genome-Wide DNA Methylation Analysis of Systemic Lupus Erythematosus Reveals Persistent Hypomethylation of Interferon Genes and Compositional Changes to CD4+ T-cell Populations

**DOI:** 10.1371/journal.pgen.1003678

**Published:** 2013-08-08

**Authors:** Devin M. Absher, Xinrui Li, Lindsay L. Waite, Andrew Gibson, Kevin Roberts, Jeffrey Edberg, W. Winn Chatham, Robert P. Kimberly

**Affiliations:** 1 HudsonAlpha Institute for Biotechnology, Huntsville, Alabama, United States of America; 2 University of Alabama at Birmingham, Birmingham, Alabama, United States of America; National Institute of Arthritis and Musculoskeletal and Skin Diseases, United States of America

## Abstract

Systemic lupus erythematosus (SLE) is an autoimmune disease with known genetic, epigenetic, and environmental risk factors. To assess the role of DNA methylation in SLE, we collected CD4+ T-cells, CD19+ B-cells, and CD14+ monocytes from 49 SLE patients and 58 controls, and performed genome-wide DNA methylation analysis with Illumina Methylation450 microarrays. We identified 166 CpGs in B-cells, 97 CpGs in monocytes, and 1,033 CpGs in T-cells with highly significant changes in DNA methylation levels (p<1×10^−8^) among SLE patients. Common to all three cell-types were widespread and severe hypomethylation events near genes involved in interferon signaling (type I). These interferon-related changes were apparent in patients collected during active and quiescent stages of the disease, suggesting that epigenetically-mediated hypersensitivity to interferon persists beyond acute stages of the disease and is independent of circulating interferon levels. This interferon hypersensitivity was apparent in memory, naïve and regulatory T-cells, suggesting that this epigenetic state in lupus patients is established in progenitor cell populations. We also identified a widespread, but lower amplitude shift in methylation in CD4+ T-cells (>16,000 CpGs at FDR<1%) near genes involved in cell division and MAPK signaling. These cell type-specific effects are consistent with disease-specific changes in the composition of the CD4+ population and suggest that shifts in the proportion of CD4+ subtypes can be monitored at CpGs with subtype-specific DNA methylation patterns.

## Author Summary

We have analyzed DNA methylation, an epigenetic modification that influences gene expression, in lupus patients and control subjects. Our analysis was run in three different immune cell types, T-cells, B-cells, and monocytes, to discern common epigenetic effects in lupus from cell type-specific effects. We have identified a lupus-related reduction in methylation around genes that respond to interferon, a cytokine that induces inflammation in response to pathogens. This hypomethylation suggests that lupus patients are hypersensitive to interferon, as DNA methylation is typically an inhibitor of gene expression. We also find that this hypersensitivity is preserved in lupus patients beyond active stages of the disease, and this may help explain the chronic, recurrent nature of the disease. In addition, we have identified DNA methylation changes in T-cells that suggest an alteration in the proportions of these cells in lupus patients, which may help explain the disease process.

## Introduction

Systemic lupus erythematosus (SLE) is a complex autoimmune disease characterized by an impaired clearance of apoptotic cells, the production of auto-antibodies against nuclear antigens, and the deposition of immune complexes that lead to tissue damage in multiple organs. SLE patients suffer from chronic dermatological, musculoskeletal, renal, and cardiovascular problems, and like many autoimmune diseases, these symptoms typically worsen during periods of active disease, called flares, and improve during quiescent phases of the disease. SLE predominantly affects females (∼90% of cases), and is more prevalent in individuals of African descent [1].

SLE is known to have a strong genetic basis, with high sibling risk ratios (λ_s_>8) and higher concordance among monozygotic twins compared to dizygotic twins or full siblings [2]–[4]. Recent genetic studies, including genome-wide association studies, have identified multiple common genetic risk factors, the strongest of which are in the MHC region of chromosome 6, but also include ITGAM, IRF5, STAT4, and at least twenty other genes [5]–[10]. While a few rare variants of strong effect have been identified, the currently favored hypothesis is one of complex etiology involving multiple genetic and environmental risk factors.

Given the complex nature of SLE etiology, epigenetic analyses are likely to provide new insights into the disease, as chromatin structure and DNA methylation patterns are influenced both by the inherited DNA sequence and by environmental exposures. In fact, the importance of DNA methylation in lupus has been appreciated for over 20 years. T-cells from patients with SLE have reduced expression of DNA methyltransferases [11], and DNA methylation inhibitors like 5-azacytidine can induce T-cell autoreactivity and lupus symptoms in mice [12]. Furthermore, drug-induced lupus is associated with reduced DNA methylation and aberrant expression of DNA methyltransferases [13].

A few recent studies have been published on genome-wide analyses of DNA methylation patterns in SLE. These include studies of a few thousand CpGs in CD4+ T-cells from discordant monozygotic twins [14], and either buffy coat DNA or sorted CD4+ T-cells from unrelated individuals [15], [16]. Here, we report the most comprehensive study to date of SLE epigenetics, where we have analyzed >460,000 CpGs, covering >95% of known genes, in CD4+ T-cells, CD19+ B-cells and CD14+ monocytes. Our results uncover a profound hypomethylation of genes regulated by interferon (type I) that is present in patients during and after flares, suggesting that this epigenetic state persists beyond stages when circulating interferon levels are at their highest. Our results also suggest a compositional remodeling of the CD4+ T-cell population in SLE patients that can be observed in DNA methylation patterns.

## Results

To search for epigenetic risk factors for SLE, we performed genome-wide DNA methylation analysis of 49 patients with SLE and 58 control individuals with no known autoimmune disease. The patients were all seen at the UAB Rheumatology Clinic and diagnosed according to the revised ACR criteria (see Materials & Methods). Tables S1 and S2 describe the gender, age, and ethnic makeup of our initial SLE and control samples. Approximately 15 ml of peripheral blood was collected from each of subject, and the blood sample was split into aliquots for isolation of specific cell-types by positive selection with antigen-specific magnetic beads. We collected CD4+ T-cells and CD19+ B-cells from all individuals, and CD14+ monocytes from approximately half of our subjects (27 SLE patients and 27 controls). DNA from each cell type was analyzed with the Illumina Methylation450 array platform to assess DNA methylation genome-wide. After extensive quality filtering, batch normalization, and chemistry correction, we performed linear regression analysis at each CpG, in each cell type independently, to test for differences in DNA methylation levels between patients and controls. Our regression models also included covariates for age, gender and ethnicity at autosomal CpGs (See Materials & Methods). On the X-chromosome, we limited our analysis to females due to the inherent gender differences in methylation due to X-inactivation. These association tests identified highly significant methylation differences (p < 1×10^−8^) at autosomal CpGs in all three cell-types, including 1,033 CpGs in T-cells, 166 CpGs in B-cells, and 97 CpGs in monocytes, where our smaller sample size provides us reduced power. At this p-value significance threshold, our FDR is less than 0.0005% in T-cells, 0.003% in B-cells, and 0.012% in monocytes. Table S3 lists these highly significant CpGs. At sites with the strongest disease association, the mean difference in methylation between SLE patients and controls after covariate corrections was as high as 40%, and p-values were observed below 1×10^−20^ in T- and B-cells, and 1×10^−12^ in monocytes. These large shifts in methylation were almost entirely composed of hypomethylation events where SLE patients showed lower methylation than controls.

**Figure 1:**
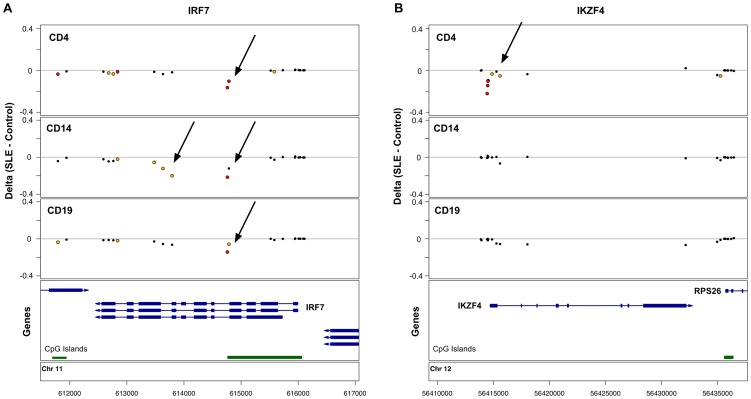
Common and cell type-specific DNA methylation changes in SLE. Differences in mean methylation between SLE and controls are plotted for each cell type at each probe near two genes. **A.** The IRF7 gene shows hypomethylation across all three cell-types at a CpG island, plus monocyte-specific hypomethylation further into the gene body. **B.** The IKZF4 gene shows T-cell-specific hypomethylation at the 5′ end of the gene. Red dots indicate p<1×10^−8^. Yellow dots indicate FDR<1%.

Many of the CpGs that showed the strongest effects were clustered within 5 kb of the same genes, such that 622 genes in T-cells, 95 genes in B-cells, and 27 genes in monocytes were strongly associated with lupus in our study. 50 genes were highly significant in both T- and B-cells, and 19 genes were highly significant across all 3 cell-types. This shared gene list expands to 60 genes if we identify those where at least one CpG is highly significant (p<1×10^−8^) in at least one of the cell types, and there is at least one moderately significant CpG in the other cell types. We defined moderate significance at CpGs where the false discovery rate was <1%, corresponding to p-values of less than 3.6×10^−4^, 3×10^−5^, and 4×10^−6^, in T-cells, B-cells, and monocytes, respectively. This list of 60 shared genes and the significant CpGs within them are shown in Table S4. Although these 60 genes contained SLE-related methylation changes in all three cell-types, the effects were not always observed at the same CpG, and there were numerous examples of cell type-specific effects, even within these shared-effect genes. For example, IRF7 contains both common and cell type-specific methylation changes in SLE patients (Figure 1A).

In addition to the genes with SLE-related methylation differences in all three cell-types, there were 446 genes in T-cells and 7 genes in B-cells with highly significant effects in that cell-type alone. Figure 1B illustrates one such example at IKZF4, where only T-cells show hypomethylation in SLE patients at multiple CpGs near the transcription start site. These cell type-specific genes are listed in Table S5.

**Figure 2:**
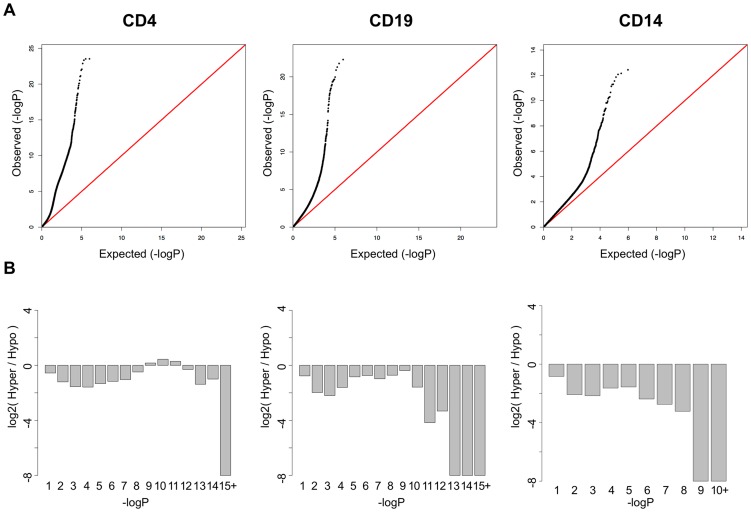
SLE QQ-Plots and the ratio of hyper- and hypomethylation events. **A.** QQ-Plots of the p-values from the SLE association analysis for each cell-type, with a unique inflation in CD4+ T-cells. **B.** Log2 ratios of hyper-to-hypomethylated CpGs within bins of the p-value distribution for each cell type, showing a unique enrichment for hypermethylation among the significant CpGs in CD4+ T-cells.

In addition to the CpGs with strong, highly significant effects, QQ plots of the p-values in each cell type (Figure 2A) suggested that T-cells had many more mild disease associations than either B-cells or monocytes. We found that >16,000 CpGs were significant at an FDR less than 1% in T-cells, while 1,403 and 199 were significant at this threshold in B-cells and monocytes, respectively. In addition, the QQ plot for T-cells displayed an unusual inflation between p-values of 1×10^−5^ and 1×10^−11^, indicating a bi-phasic p-value distribution and suggesting that two overlapping phenomenon were occurring in this cell-type. Most of these CpGs displayed milder shifts in methylation between patients and controls, typically less than 10%. Furthermore, when we plotted the log ratio of hypomethylated to hypermethylated CpGs across bins of the p-value distribution (Figure 2B), we found that T-cells contained a unique shift toward hypermethylation at these lesser effect CpGs. This observation supported the hypothesis that two independent phenomena were occurring in T-cells. These CpGs in this secondary phase of p-values also had a unique distribution of mean methylation levels, both in SLE cases and controls. While the CpGs represented on the Illumina Methylation450 array have a bimodal distribution of methylation, with most CpGs carrying less than 20% or greater than 80% methylation (Figure S1A), the SLE-associated CpGs in T-cells were heavily enriched for intermediate methylation levels. As seen in Figure S1B, the majority of these CpGs had mean methylation levels between 20% and 80%, both in SLE patients and controls. This may indicate that these CpGs are either sites of dynamic regulation with fluctuating methylation levels that average to an intermediate level, or that they are sites with methylation levels that are specific to subpopulations of CD4+ T-cells, and that the mixed population of CD4+ subsets gives rise to an observation of intermediate methylation levels. A third possibility is that these CpGs are maintained in all cells at an intermediate methylation level, similar to an imprinted locus where only one allele is methylated. However, we found no enrichment for imprinted genes near these CpGs (data not shown).

### Functional Analysis

To identify common functional characteristics of genes with aberrant DNA methylation in SLE patients, we performed DAVID Panther GO term analysis and Ingenuity Pathway Analysis (IPA) on the genes that were proximal to each of the most significant (top 100) CpGs in each cell type. Both analyses clearly identified interferon signaling as a common feature of the genes showing the most significant changes in methylation among SLE patients. Table 1 and Table S7 list the results of these analyses, including the top Panther GO terms and IPA canonical pathways. IPA also indicated the type-I interferon IFNA2 (interferon alpha 2) as a common upstream regulator, so we suspect that type-I interferon pathways are the targets of the epigenetic changes in lupus. However, IL-29 (IFNL1) was also significant as a potential upstream regulator of these genes, so it is possible that both type-I and type-III interferons are contributing to the epigenetic patterns we observed.

**Table 1:** Functional analysis of significant CpGs in three cell types.

DAVID/Panther GO Term	p-value	FDR %
**Genes near top 100 CD4 CpGs**		
BP00156: Interferon-mediated immunity	5.80E-07	5.90E-04
BP00148: Immunity and defense	3.80E-03	3.80422
**Genes near top 100 CD14 CpGs**		
BP00156: Interferon-mediated immunity	2.20E-08	2.10E-05
BP00148: Immunity and defense	8.60E-08	8.20E-05
**Genes near top 100 CD19 CpGs**		
BP00156: Interferon-mediated immunity	9.00E-07	9.20E-04
BP00148: Immunity and defense	2.20E-05	2.20E-02
**Genes near CD4 CpGs with 1E-08>P>1E-11**		
BP00064: Protein phosphorylation	4.09E-04	0.4938
BP00281: Oncogenesis	6.16E-04	0.7434
BP00152: B-cell and antibody-mediated immunity	0.0014738	1.7689
BP00063: Protein modification	0.0017254	2.0679
BP00253: Induction of apoptosis	0.0021886	2.6164
BP00148: Immunity and defense	0.0022203	2.6538
BP00207: Cell cycle control	0.0030120	3.5843

Results from DAVID/Panther GO term analysis for the highly significant CpGs in each cell type and the mildly significant CpGs in T-cells.

As Panther and IPA use different gene annotations, their lists of interferon-regulated genes are not identical. Furthermore, many of the putative interferon-inducible genes (IFI44, IFITM1, etc.) are not always properly annotated with interferon GO terms. When we combined the gene lists from each software package with type-I interferon annotations and included these “IFI” genes, we found that at least half of the top 50 most significant CpGs in each cell type were proximal to genes involved in interferon signaling (50% in T-cells, 60% in B-cells, and 54% in monocytes). This represents more than 125 fold enrichment over the ∼0.4% of autosomal CpGs represented on the Methylation450 array that are adjacent to interferon type-I genes (Fisher's exact test p<5×10^−46^). Remarkably, of the 63 CpGs in T-cells, 58 CpGs in B-cells, and 23 CpGs in monocytes that had highly significant changes in methylation (p < 1×10^−8^) near an interferon type-I regulated gene, only 1 CpG in B-cells, located at the 3′ end of STAT3, was hypermethylated in SLE patients. Every other highly significant methylation change near an interferon gene was a hypomethylation effect. This widespread hypomethylation suggests that the primary methylation defect in SLE is a hyper-sensitization of interferon signaling pathways, and this is consistent with gene expression studies that have shown an overexpression of interferon-regulated genes in SLE patients, particularly during flares of the disease [17]–[19].

In addition to the most significant CpGs, we also performed a separate functional analysis of genes in the second phase of the T-cell p-value distribution (limited to p-values between 1×10^−8^ and 1×10^−11^), where we suspect a secondary phenomenon. Both Panther and IPA analyses indicated that these genes were enriched for functions associated with cell division and cancer. IPA specifically identified the p38 mitogen-activated protein kinase pathway as a common feature of these genes, a pathway that has been linked to autoimmune diseases, including SLE [20]. This functional difference between the two phases of the p-value distribution, in addition to the enrichment for hypermethylation effects and intermediate mean methylation levels, is further evidence that two independent phenomena were present in T-cells.

### Disease Activity

**Figure 3:**
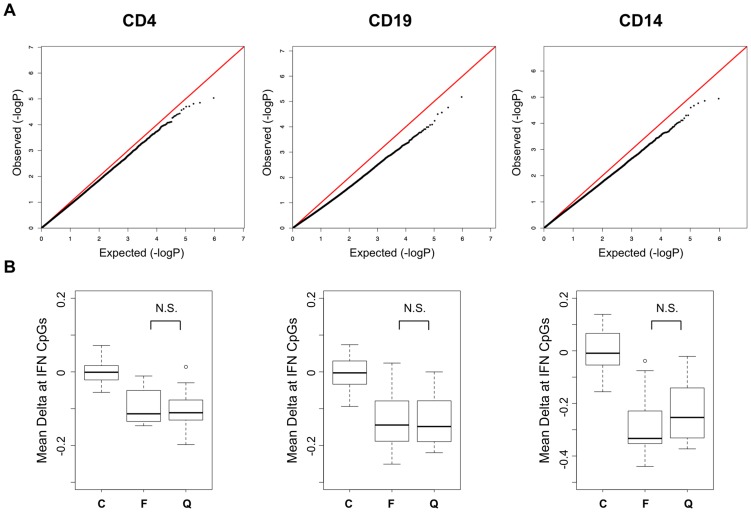
Disease activity QQ-Plots and the persistence of hypomethylation in quiescent patients. **A.** QQ-Plots of the p-values from the flare versus quiescent association analysis for each cell type, illustrating the lack of activity-dependent DNA methylation. **B.** Boxplots of the methylation difference between each individual and the mean of all controls at CpGs in IFN-regulated genes among those that were highly significant in the SLE-control tests. The groups are labeled C, Control, F, SLE collected during a flare, and Q, SLE collected during quiescence.

Previous reports of increased expression of interferon-regulated genes in SLE patients have indicated that this effect is primarily observed during active phases of the disease, while those patients in quiescent phases have normal levels of expression. This observation coincides well with reports that circulating interferon levels correlate with disease activity [21]. We compared the DNA methylation levels between our active and quiescent SLE patients to identify activity-dependent methylation in these patients that might coincide with this gene expression effect. We performed regression analysis in a case-case comparison of flare versus quiescent SLE patients. As seen in the QQ plot from these association tests (Figure 3A), we found no significant differences between these groups. Regression analyses of methylation versus SLEDAI scores as continuous values were also negative (data not shown). Even the strong hypomethylation at interferon-regulated genes was similar in active and quiescent patients (Figure 3B), with no statistically significant difference between the disease groups in any cell type. These results indicate that the methylation changes in SLE persist beyond flares and may be maintained for many months after interferon levels normalize. It also indicates that SLE patients in quiescent stages remain poised for interferon response at an epigenetic level, with a significant number of immune cells carrying this phenotype.

### CD4+ T-cell Subsets

**Figure 4:**
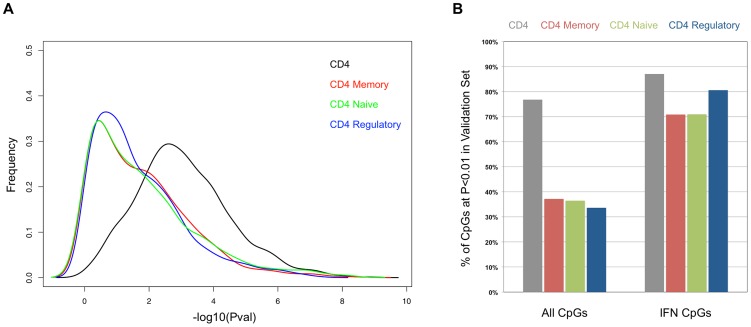
Comparison of the SLE-control p-values in the sorted T-cells from the validation cohort. (**A**) Distribution of the −log10 of the p-values from association tests in the sorted T-cells cells from the validation cohort at CpGs that were highly significant (p<1×10^−8^) in the initial cohort. (**B**) Percentage of CpGs that reported p<0.01 in the sorted T-cells cells from the validation cohort. Values in the left set of bars are from all 1,031 CpGs tested. Values in the right set of bars are from the subset of 62 CpGs near interferon-regulated genes.

**Figure 5:**
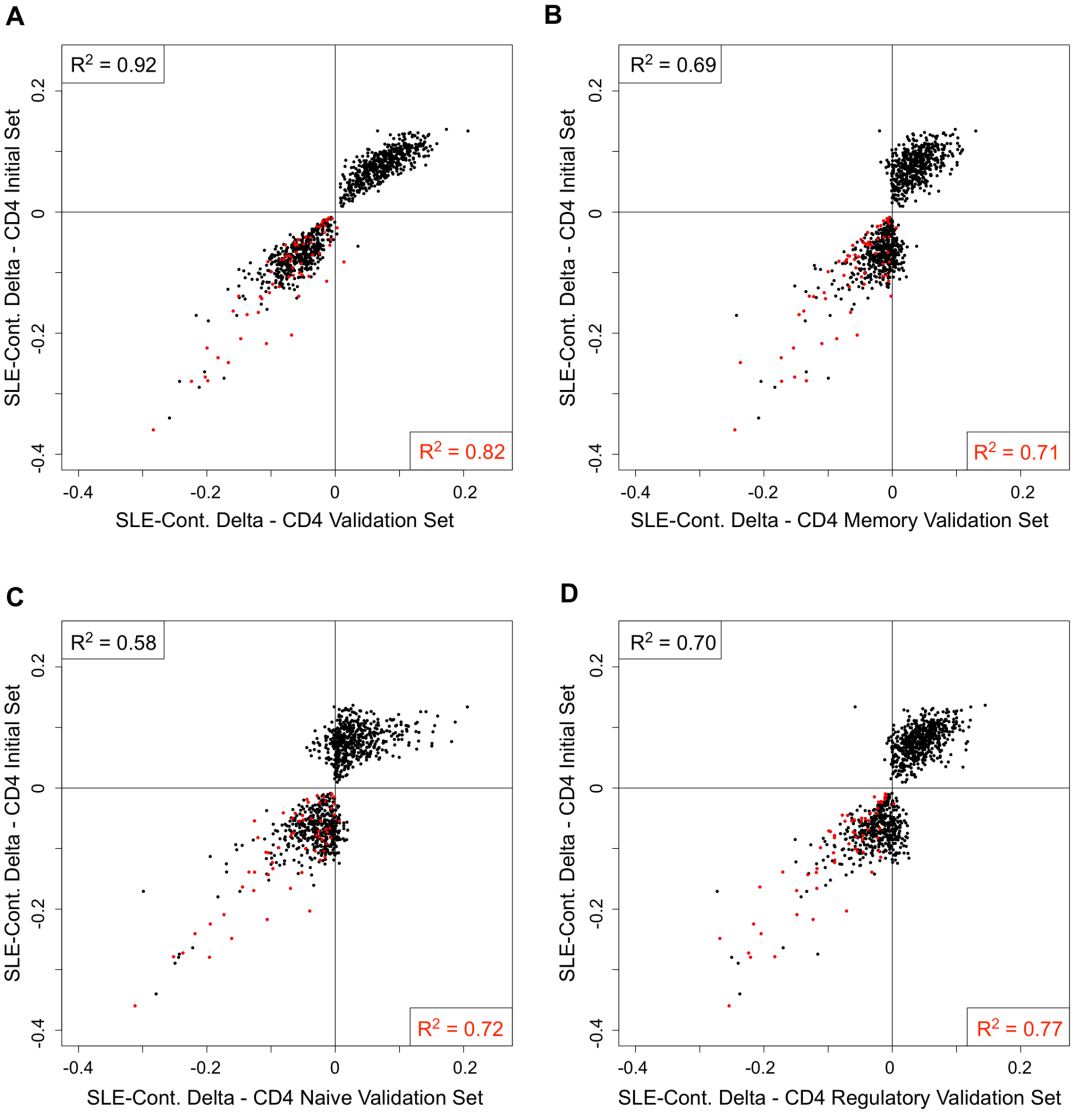
Comparison of the SLE-control methylation differences in sorted T-cell populations. Each scatter plot represents 1,031 CpGs that had p<1×10^−8^ in CD4+ T-cells in our SLE-control association tests. The Y-axis for all plots is the mean SLE-control methylation delta at these CpGs in the initial cohort. The X-axis for each plot is the mean SLE-control methylation delta at the same CpGs in our validation cohort, using (**A**) total CD4+, (**B**) CD4+Memory, (**C**) CD4+Naïve, or (**D**) CD4+Regulatory cells. The red dots represent CpGs near IFN-regulated genes and the squared correlation coefficients (R^2^) represent the values for all plotted CpGs (upper left) or IFN CpGs only (lower right).

One possible explanation for the persistent hypomethylation of interferon-regulated genes could be the endurance of memory cells that carry this epigenetic state since the last flare. Furthermore, some of the methylation changes we observed might be specific to T-cell subtypes, rather than a general feature of the CD4+ pool. To examine these possibilities, we collected CD4+ T-cells from an independent cohort of 26 SLE patients and 18 controls, and further sorted a fraction of these into CD45RA+RO− naïve, CD45RA-RO+ memory, and CD25+CD127− regulatory T-cells. To ensure that this independent validation set recapitulated the results from our initial cohort, we re-tested for SLE-related methylation changes in CD4+ T-cells using our regression model at 1,031 CpGs that were highly significant in the initial cohort (2 of the original 1,033 failed QC in the validation set). Despite the smaller size of the validation set, 76.8% of the CpGs were significant at p<0.01 in these validation tests (see Figure 4A, black line, and 4B, gray bar). Furthermore, a comparison of the direction and amplitude of the changes in methylation observed in the SLE patients' T-cells indicated a very high correlation with the initial cohort (R^2^ = 0.92, see Figure 5A). These tests strongly validate our initial findings in an independent cohort.

We next tested for SLE-related methylation changes in the sorted T-cell subsets from the same individuals from our validation cohort. If any of the observed methylation changes were specific to memory, naïve, or regulatory T-cells, the enrichment of these cell types should reveal a stronger effect than is seen in the CD4+ pool as a whole. However, when we ran our regression tests on the same 1,031 CpGs in the sorted subsets, the distribution of p-values indicated much weaker effects than those seen in the CD4+ pool (Figure 4A). The number of CpGs that validated at p<0.01 was less than 38% in all three sorted subtypes, or approximately half of that observed in the CD4+ pool from the same individuals (Figure 4B). Furthermore, the correlations in direction and amplitude of the SLE-related methylation changes were weaker in the sorted CD4+ subtypes, where the R^2^ dropped below 0.70 for each sorted cell type (Figure 5 B–D).

When we limited our analysis to only those significant CpGs near interferon-regulated genes, the trend was dramatically different. The number of these CpGs that validated at p<0.01 was similar in the sorted subtypes (71% in naïve and memory, 81% in regulatory) compared to the CD4+ pool as a whole (87%) (Figure 4B). Furthermore, the direction and amplitude correlations with the initial CD4+ results were stronger at the interferon CpGs than at the non-interferon CpGs (Figure 5 B–D, red dots), but no stronger than the correlations observed in the CD4+ population as a whole. This suggests that the methylation changes we observed at interferon-regulated genes in CD4+ T-cells are intrinsic to memory, naïve, and regulatory T-cells, but not specific to any one population. So, it is unlikely that the persistence of these changes during quiescent stages of SLE can be explained simply by the endurance of memory cells. Furthermore, since the milder changes in methylation at non-interferon loci that were observed in the CD4+ T-cells, appear to be absent or greatly diminished in the sorted subtypes, the observed differences in methylation are not likely to be intrinsic to memory, naïve or regulatory T-cells. Thus, the most likely explanation for the widespread, moderate changes at thousands of CpGs in the CD4+ T-cells is a change in the composition of the CD4+ pool. Changes in the proportions of CD4+ subtypes in SLE patients would generate disease associations at any loci that had cell type-specific methylation patterns, and as we have observed, these loci would likely have intermediate mean methylation levels due to the mixture of these cell types in the CD4+ population. For example, a 10% methylation difference between SLE patients and controls could be due to a 50% difference in methylation within a CD4+ subtype that makes up 20% of CD4+ cells. Conversely, the same 10% methylation difference could be generated if that same subtype dropped in number among SLE patients to alter the composition of the CD4+ population. Any CpG with a subtype-specific methylation pattern would show this trend. Our data on sorted CD4+ subsets is consistent with the latter, as we observe a reduction, rather than an enrichment of the SLE-control methylation differences, as we purify CD4+ subtypes.

### X-chromosome

The analysis of X-chromosome methylation is hampered by the inherent differences in methylation between males and females, so our disease association tests on this chromosome were limited to females, in which we have the largest sample size. For this reason, it is difficult to compare test statistics to those at the autosomal CpGs. Nonetheless, we ran regression tests at 11,122 X-chromosome CpGs to compare female SLE patients to females controls. Only in T-cells did we observe moderately significant associations (FDR<1%), although none were genome-wide significant (p<1×10^−8^). Table S6 lists the 43 significant X-chromosome CpGs in T-cells. These include TLR7 and FOXP3, both of which have been previously linked to SLE.

## Discussion

We have performed a comprehensive analysis of DNA methylation changes in SLE in two lymphoid cell-types (T- and B-cells), and one myeloid cell-type (monocytes). Our analysis has identified a strong hypomethylation of loci involved in type-I interferon signaling, which indicates that SLE patients are hypersensitive to interferon. While this is not entirely surprising, given that interferon-related gene expression changes have been documented in active SLE patients, we have also discovered that the hypomethylation is observed in both active and quiescent patients. This is remarkable because circulating interferon and the expression of the genes it induces, are known to increase during flares of the disease, but return to normal during quiescent periods. So, the epigenetic hypersensitivity at the DNA methylation level appears to be independent of interferon levels and is maintained in the immune system beyond active stages of the disease. Exactly when these epigenetic changes occur is not clear. Studies have demonstrated mildly elevated IFN-α in unaffected relatives of SLE patients, suggesting that there is a genetic basis of higher IFN levels [22]. So it is feasible that SLE patients had higher baseline IFN prior to disease onset, and that chronic exposure could have induced long-lasting epigenetic hypersensitivity. In any case, the persistence of the hypomethylation in patients during quiescence is important, as it may help explain the chronic nature of the disease and the potential for recurrent flares in SLE patients. Our data suggest that these patients are poised for elevated interferon responses, but until some event triggers IFN-α production, the responsive genes remain near normal expression levels.

We have also observed the hypomethylation of interferon genes in sorted subpopulations of CD4+ T-cells, including memory, naïve and regulatory T-cells. Given that this appears to be a universal effect, and is apparent in lymphoid and myeloid lineages, the most likely explanation is that a multi-potent progenitor population carries this epigenetic state and produces lineages that are programmed to respond to interferon. Future studies of DNA methylation in early progenitor populations from SLE patients will be needed to establish the responsible cells, and to define the events that might induce this epigenetic state in progenitor cells.

 In addition to the primary interferon effect, we have identified widespread moderate changes in methylation in T-cells that are best explained by SLE-related compositional changes to the CD4+ population, rather than intrinsic methylation changes in any CD4+ subtype. We did not observe an enrichment of these effects in sorted memory, naïve or regulatory T-cells, although we cannot rule out a role for subtypes such as Th1, Th2, or Th17, as we did not sort along these lines. This is not to suggest that methylation effects are absent from CD4+ subtypes, but rather that the widespread, moderate changes we can observe in the CD4+ population cannot be explained solely by intrinsic methylation changes in memory, naïve or regulatory T-cells. Further sub-fractionation of the CD4+ cells will be required to establish which subtypes are responsible for these subtle changes in SLE patients, either because they carry subtype-specific methylation patterns and are changing in number, or because they carry intrinsic methylation differences in SLE patients. Some studies have indicated that regulatory T-cells are reduced in number in SLE patients. While this may be one contributor to the compositional effect, our quantification of memory, naïve and regulatory T-cells is insufficient to explain the entirety of the methylation changes we observe in CD4+ cells. Our functional analysis of the genes affected by these methylation changes, indicate that they are involved in immune cell signaling and cell division. All of these might be interpreted as part of the T-cell activation process, and perhaps the compositional changes occurring in the CD4+ population are due to increases in the number of activated T-cells that cut across traditional definitions of the CD4+ subsets. A complete characterization of the genome-wide DNA methylation profiles in the CD4+ milieu will be required to understand how different epigenetic states correlate with classic cell type definitions.

Finally, while our study was not designed to detect methylation patterns that were induced by medications or might be predictive of a patient's response to medications, this is clearly an area of great interest. The fact that we observe similar methylation patterns in quiescent and active SLE patients, who typically increase their medication levels during a flare, suggests that these medications do not induce a large epigenetic effect. Nonetheless, studies that examine the epigenetic impact of anti-inflammatories, as well as the epigenetic states that modulate their efficacy, may have an impact on the clinical management of SLE.

## Materials and Methods

### Ethics Statement

All patient samples were collected with consent at UAB under compliance with the Institutional Review Board.

### Patient Samples

Patients were recruited through the UAB outpatient Rheumatology clinic. Diagnosis was performed according to revised ACR criteria [23]–[25] and disease activity and SLEDAI scores were collected from each patient, along with gender, age and ethnicity information. Disease activity (flare versus quiescent) was defined by a recent increase in SLEDAI without using a specific SLEDAI threshold. However, all patients considered to be active had a SLEDAI > = 4 (mean = 8.5), and all of our quiescent patients had a SLEDAI < = 6 (mean = 1.5).

### Cell and DNA Isolation

CD4+ T-cells, CD19+ B-cells and CD14+ monocytes were isolated from ∼5 ml each of freshly collected peripheral blood. All three cell types were isolated in parallel using positive selection by antigen-specific Dynabeads (Invitrogen), according to the manufacturer's standard protocol. The cells captured on the beads were lysed and DNA was extracted with QIAGEN DNAeasy kits. Purity of separated populations was verified to be above 95%.

 For experiments with CD4+ subsets, CD4+ cells were isolated using positive selection (Invitrogen) followed by sorting of subsets by flow cytometry (FACSAriaII, BD Biosciences). Very pure populations (95–100%) of memory T cells (CD45RO+RA−), naïve T cells (CD45RA+RO−), and T regulatory cells (CD25+CD127−) were collected using anti-CD4-Alexa488, anti-CD45RO-APC, anti-CD45RA-PE, anti-CD25-PerCP-Cy5.5 and anti-CD127-Pacific Blue antibodies (Biolegend, Inc). Cells were then lysed and DNA extracted with QIAGEN DNAeasy kits.

### Methylation450 Assays, Data QC and Batch Normalization

500 ng of each DNA sample was treated with sodium bisulfite (Zymo EZ DNA) prior to standard Illumina amplification, hybridization, and imaging steps. To limit confounding from batch effects, we distributed SLE cases and controls equally among the 12 slots on each array. The samples were also grouped on the arrays by cell type. The resulting intensity files were analyzed with Illumina's GenomeStudio, which generated beta scores (proportion of total signal from the methylation-specific probe or color channel) and “detection p-values” (probability that the total intensity for a given probe falls within the background signal intensity). Beta scores were generated without background subtraction or Illlumina normalization options. Those beta scores with an associated detection p-value greater than 0.01 were removed and samples with more than 1.5% missing data points across ∼470,000 autosomal CpGs were eliminated from further analysis. Furthermore, any CpG probes where more than 10% of samples failed to yield adequate intensity were removed.

The filtered beta scores were then subjected to non-parametric batch normalization with the ComBat package for R software (http://http://www.bu.edu/jlab/wp-assets/ComBat/Abstract.html). To parallelize this process on our computational cluster, normalization was performed on non-overlapping subsets of no more than 20,000 CpGs per job (randomly selected), and each array of 12 samples was used as a “batch”. We also separately normalized probes from the Infinium I and II chemistries, as their beta score distributions are slightly different. For example, the 131,715 autosomal Infinium I CpGs were split into 6 randomly chosen sets of 20,000 CpGs each, plus one set of 11,715 CpGs, and each set was batch normalized in parallel. Figure S3A shows QQ-plots of explicit tests for batch effects at each CpG, before and after ComBat normalization. These tests were linear regression tests for batch ID, with disease, age, gender, and ethnicity as covariates. In addition, we compared our subsetting approach of 20,000 CpGs to similar ComBat runs with larger numbers of CpGs, but the efficacy of batch correction was virtually identical, while greatly reducing the computational time for normalization. Furthermore, as indicated in Figure S3B, our batch normalization process did not introduce any systematic bias into our data, as our disease-specific regression results applied before and after ComBat were highly similar. Data from the X chromosome was normalized separately for males and females due to the gender-specific effect of X-inactivation on the beta score distribution. After batch normalization, we further adjusted the beta scores for probes that utilized the Infinium II chemistry to better match the Infinium I chemistry using the equation β′ = 0.001514 + 0.3323* β + 0.7411* β ^2^. This equation was derived from fitting a second order polynomial to the observed pairs of beta scores across all pairs of probes located <50 bp apart, where one probe was Infinium I and one was Infinium II. At this proximity, within-chemistry correlations are extremely high (R>0.99) due to locally correlated methylation patterns, and the non-linear relationship between the two chemistries is easily estimated. Figure S4 illustrates the improved scaling of the two chemistries after our corrections have been applied.

Our dataset was further reduced by eliminating any CpGs where the probe sequence either mapped to a location in the genome that was different that the location found in Illumina's annotation file, or where the probe could potentially map to more than one locus. The list of these problematic CpGs was generated by re-aligning all probes (with unconverted Cs) to the human reference genome with BLAT. We also maintained a list of probes where known SNPs would fall within the probe sequence or at the CpG itself, but did not explicitly filter out these probes. There was no apparent enrichment for CpG probes that overlapped a SNP in dbSNP 135 among our most significant results.

### Data Analysis

To perform genome-wide association testing, we ran linear regression models at each CpG (lm package in R) to test for associations between DNA methylation levels and SLE disease state (case/control comparison) or flare status (case/case comparison). Since DNA methylation is influenced by age, gender, and ethnicity, we included these as covariates in our models.
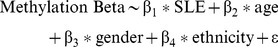
(1)


For analysis of the X-chromosome CpGs, females were analyzed separately so gender correction was unnecessary. The p-values and beta coefficients for the disease term in our regression models were used to establish the significance of the association at each CpG, and to estimate the post-correction differences in methylation between cases and controls, respectively. FDR correction was performed on the p-values using R (p.adjust function). We also selected 20,000 CpGs at random to perform permutation tests that randomized the disease state variable to estimate empirical p-values (lmp package). After 10^8^ permutations, the permutation-based p-value was compared to the regression estimate, and both p-values were highly correlated. Figure S2 displays the genome-wide QQ plot for CD4+ cells, with the permuted p-values for 20,000 random CpGs overlayed in green. The biphasic trend in the QQ plot was recapitulated with permutation-based p-values.

We performed two types of analyses (Ingenuity and DAVID) to identify gene annotation terms that were enriched among our most significant associations. Our annotation of interferon-regulated genes was expanded to include the “IFI” gene symbols, which have been termed “interferon-inducible transcripts”, but have not all been given GO terms that reflect this functionality.

## Supporting Information

Figure S1 Enrichment for intermediate beta scores at the non-IFN CpGs in T-cells. Histograms of the beta score distributions in control samples for (**A**) all autosomal CpGs on the array, (**B**) the significant (p<1×10^−8^) CpGs in T-cells, excluding CpGs near IFN genes, and (**C**) the significant CpGs near IFN genes.
(TIFF)

Figure S2 Comparison of regression p-values to permutation-based p-values. 10^8^ permutations of disease status were performed on 20,000 random CpGs and tested for disease association. The permutation-based p-values (green) were plotted over the standard regression p-values on the QQ-plot from T-cells. The values were highly correlated and the permutations recapitulated the unusual inflation pattern observed in T-cells.
(TIFF)

Figure S3 Batch correction with ComBat software. (**A**) QQ-plot of linear regression tests for batch effects at each Infinium I CpG in monocytes. Black dots are prior to ComBat normalization, and illustrate significant batch effects across the dataset. Blue, green and orange dots are after batch normalization with ComBat using 20K subsetting, 70K subsetting, or all CpGs at once, respectively. (**B**) −log10 of the p-values from our SLE-control regression analysis in monocytes are similar before and after batch normalization, with no evidence of systematic bias.
(TIFF)

Figure S4 Chemistry correction. (**A**) Distribution of beta scores from monocytes in controls among the probes from the two Infinium chemistries. (**B**) Distributions of the same beta scores after chemistry corrections have been applied.
(TIFF)

Table S1 Characteristics of SLE and control individuals. Clinical and demographic features of our primary cohort are summarized. Age values are mean +/− Std.Dev. Gender, ethnicity and activity values are the numbers of individuals in each category.
(DOCX)

Table S2 SLE and control individuals. Table of all individuals in the primary cohort, their age, gender, ethnicity, disease status, disease activity, and which cell types were successfully analyzed for each individual.
(DOCX)

Table S3 List of CpGs with aberrant methylation in any cell type. Table of CpGs with p-values<1×10^−8^ in T-cells, B-cells or monocytes. The columns for each cell type indicate mean methylation proportion after correction for all covariates in controls/SLE patients. Highly significant effects (p<1×10^−8^) have double asterisks. Mildly significant (FDR<1%) have single asterisks.
(DOCX)

Table S4 List of CpGs near 42 genes with aberrant methylation in more than one cell type. Table of CpGs near genes with at least one CpG at 1×10^−8^ in one cell type and at least one CpG at FDR<1% in at least one other cell-type. The columns for each cell type indicate mean methylation proportion after correction for all covariates in controls/SLE patients. Highly significant effects (p<1×10^−8^) have double asterisks. Moderately significant (FDR<1%) have single asterisks.
(DOCX)

Table S5 List of cell type-specific genes in T-cells and B-cells. Listed are those genes with a highly significant (p<1×10^−8^) SLE-associated methylation difference that is unique to one cell-type.
(DOCX)

Table S6 CpGs showing altered methylation on the X-chromosome in females. Listed are the top CpGs from the disease association tests on the X-chromosome in females. CpGs with FDR<1% are listed. The rightmost column indicates mean methylation proportion after correction for all covariates in controls/SLE patients.
(DOCX)

Table S7 Functional analysis of significant CpGs in three cell types. Results from Ingenuity Pathway Analysis for the highly significant CpGs in each cell type and the mildly significant CpGs in T-cells.
(DOCX)
