# Supplementary figures and images for: Genome-Wide DNA Methylation Analysis of Systemic Lupus Erythematosus Reveals Persistent Hypomethylation of Interferon Genes and Compositional Changes to CD4+ T-cell Populations
Source: PLoS Genet. 2013 Aug 8;9(8):e1003678. doi: 10.1371/journal.pgen.1003678 (PMC3738443; doi:10.1371/journal.pgen.1003678)

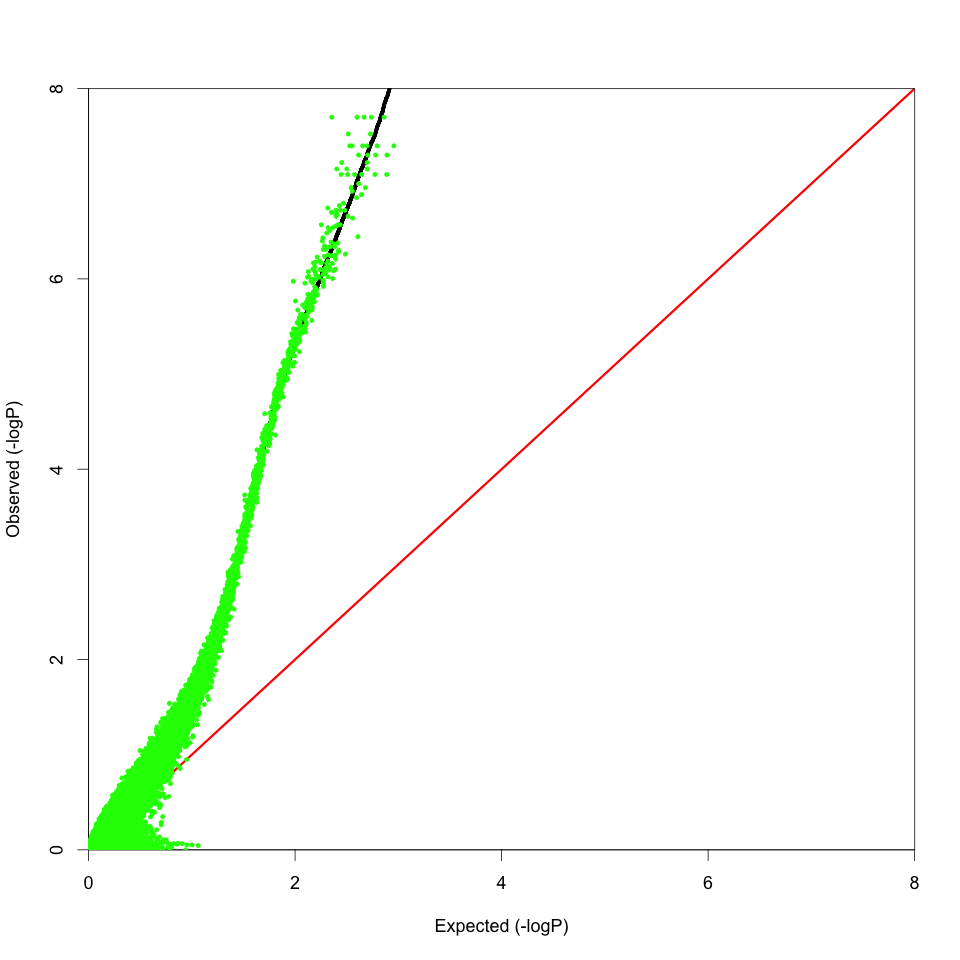

Supplement: Figure S2 — Comparison of regression p-values to permutation-based p-values. 108 permutations of disease status were performed on 20,000 random CpGs and tested for disease association. The permutation-based p-values (green) were plotted over the standard regression p-values on the QQ-plot from T-cells. The values were highly correlated and the permutations recapitulated the unusual inflation pattern observed in T-cells. (TIFF) [file pgen.1003678.s002.tiff]

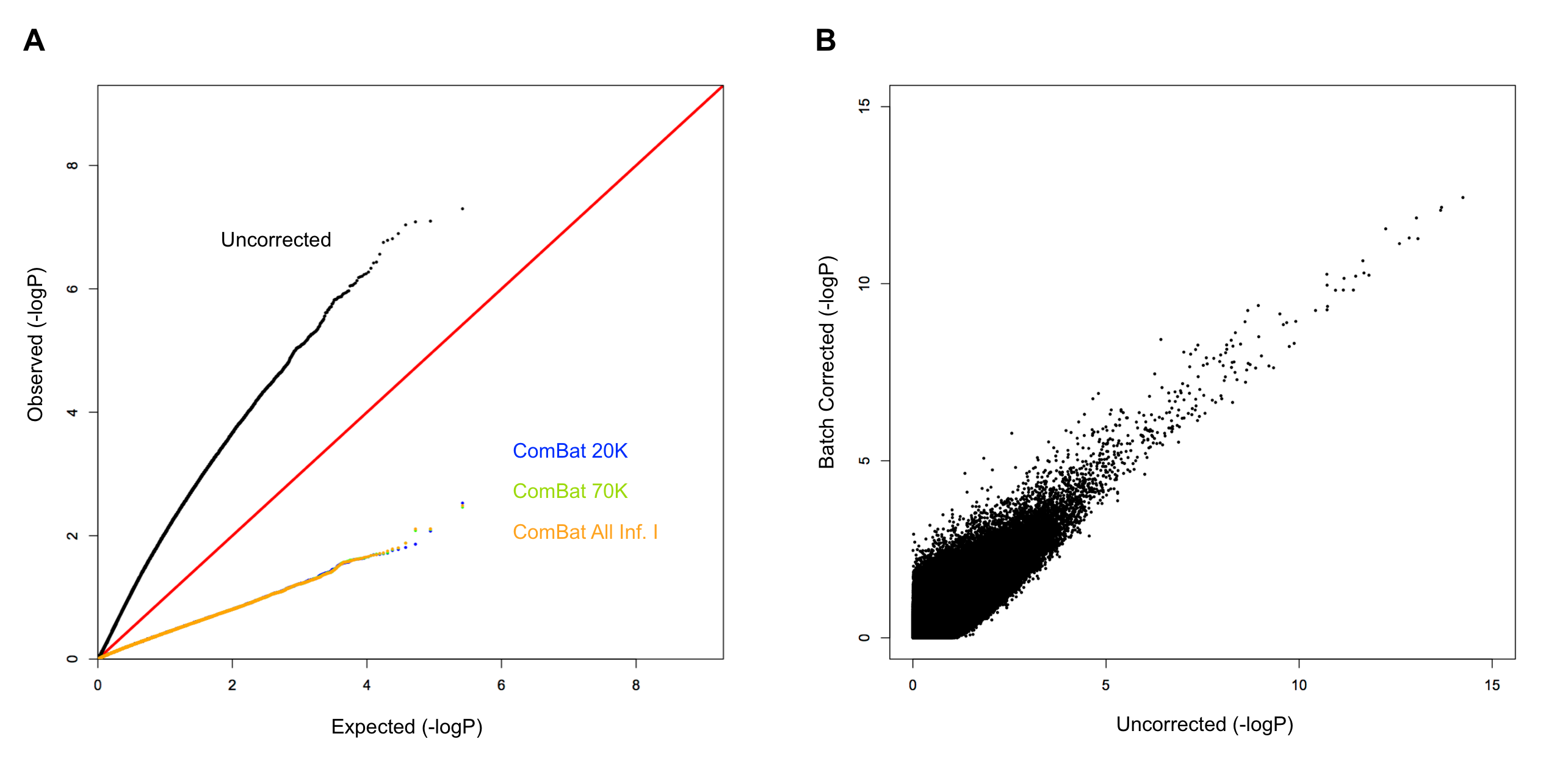

Supplement: Figure S3 — Batch correction with ComBat software. (A) QQ-plot of linear regression tests for batch effects at each Infinium I CpG in monocytes. Black dots are prior to ComBat normalization, and illustrate significant batch effects across the dataset. Blue, green and orange dots are after batch normalization with ComBat using 20K subsetting, 70K subsetting, or all CpGs at once, respectively. (B) −log10 of the p-values from our SLE-control regression analysis in monocytes are similar before and after batch normalization, with no evidence of systematic bias. (TIFF) [file pgen.1003678.s003.tiff]

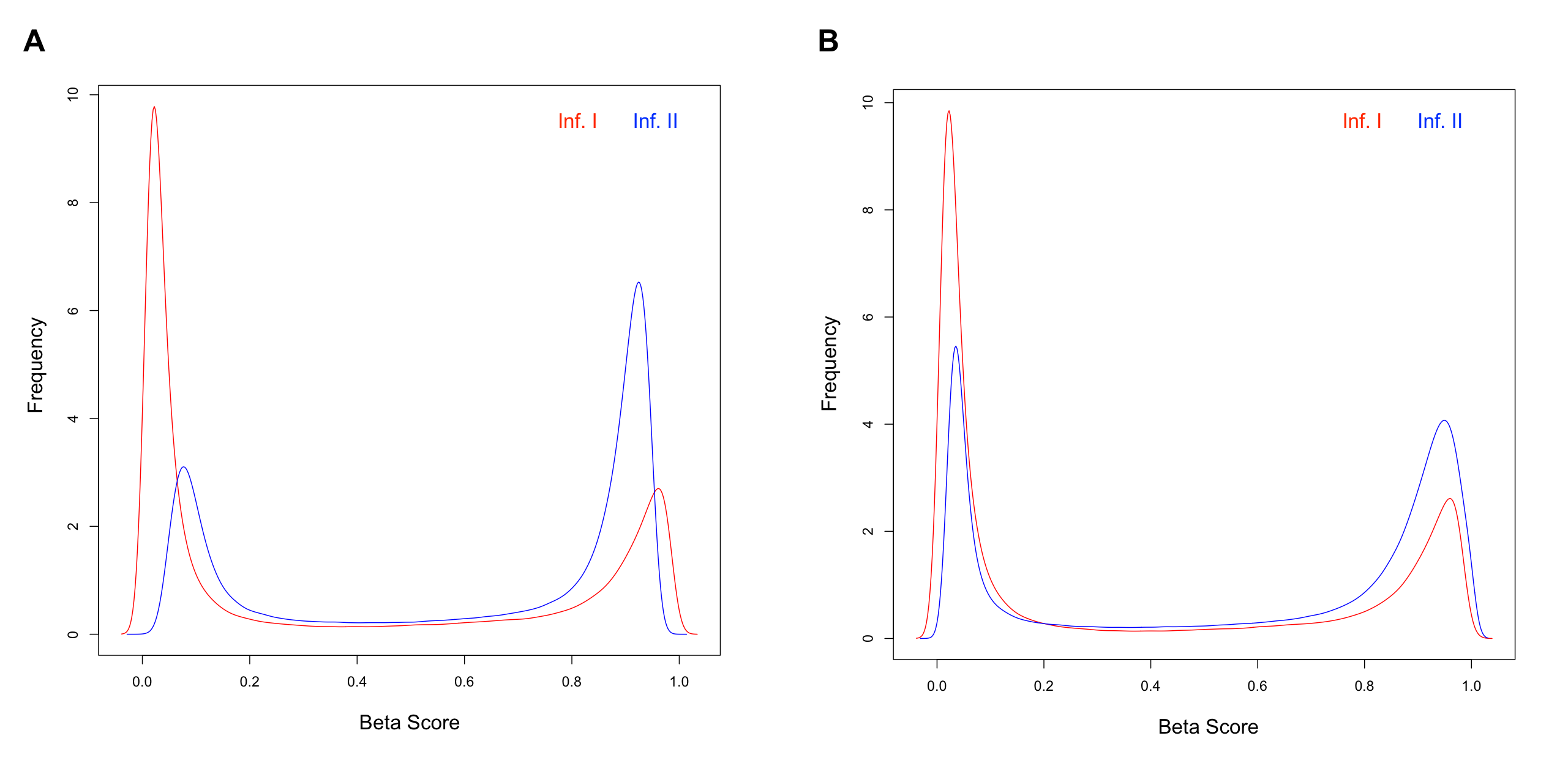

Supplement: Figure S4 — Chemistry correction. (A) Distribution of beta scores from monocytes in controls among the probes from the two Infinium chemistries. (B) Distributions of the same beta scores after chemistry corrections have been applied. (TIFF) [file pgen.1003678.s004.tiff]
