# Supplementary material for: Genome-Wide DNA Methylation Analysis of Systemic Lupus Erythematosus Reveals Persistent Hypomethylation of Interferon Genes and Compositional Changes to CD4+ T-cell Populations
Source: PLoS Genet. 2013 Aug 8;9(8):e1003678. doi: 10.1371/journal.pgen.1003678 (PMC3738443; doi:10.1371/journal.pgen.1003678)
Supplement: Table S1 — Characteristics of SLE and control individuals. Clinical and demographic features of our primary cohort are summarized. Age values are mean +/− Std.Dev. Gender, ethnicity and activity values are the numbers of individuals in each category. (DOCX) [file pgen.1003678.s005.docx]

**Table S1. Characteristics of SLE and control individuals**.

|  | **N** | **Age +/- SD** | **Gender** | **Ethnicity** | **Flare/Quiescent** | **SLEDAI range** |
| --- | --- | --- | --- | --- | --- | --- |
| **SLE** | 49 | 31.6 +/- 8.9 | 42F; 7M | 34 AA; 15 EA | 13 F; 34 Q | 0-17 |
| **Controls** | 57 | 30.8 +/- 8.4 | 43F; 14M | 34 AA; 23 EA | NA | NA |

Clinical and demographic features of our primary cohort are summarized. Age values are mean +/- Std.Dev. Gender, ethnicity and activity values are the numbers of individuals in each category.
