# Supplementary material for: Genome-Wide DNA Methylation Analysis of Systemic Lupus Erythematosus Reveals Persistent Hypomethylation of Interferon Genes and Compositional Changes to CD4+ T-cell Populations
Source: PLoS Genet. 2013 Aug 8;9(8):e1003678. doi: 10.1371/journal.pgen.1003678 (PMC3738443; doi:10.1371/journal.pgen.1003678)
Supplement: Table S2 — SLE and control individuals. Table of all individuals in the primary cohort, their age, gender, ethnicity, disease status, disease activity, and which cell types were successfully analyzed for each individual. (DOCX) [file pgen.1003678.s006.docx]

**Table S2. SLE and Control Individuals**.

| **Sample** | **AGE** | **GENDER** | **ETH** | **SLE** | **Flare/Quiescent** | **CD4** | **CD19** | **CD14** |
| --- | --- | --- | --- | --- | --- | --- | --- | --- |
| SLE 10011 | 22 | F | AA | Y | Q | X | X | X |
| SLE 10024 | 30 | F | EA | Y | Q | X | X |  |
| SLE 10347 | 23 | F | AA | Y | Q | X | X | X |
| SLE 10374 | 20 | F | AA | Y | F | X | X |  |
| SLE 10494 | 31 | F | EA | Y | Q | X | X |  |
| SLE 10495 | 26 | F | AA | Y | Q | X | X |  |
| SLE 10593 | 37 | F | EA | Y | F | X | X | X |
| SLE 10643 | 20 | F | EA | Y | Q | X | X | X |
| SLE 1072 | 47 | F | EA | Y | Q | X | X | X |
| SLE 10797 | 21 | F | AA | Y | F | X | X | X |
| SLE 10984 | 23 | M | AA | Y | Q | X | X | X |
| SLE 1120 | 29 | M | AA | Y | Q | X | X |  |
| SLE 1121 | 32 | F | AA | Y | F | X |  | X |
| SLE 11352 | 32 | F | EA | Y | Q | X | X | X |
| SLE 11414 | 29 | F | AA | Y | Q | X | X |  |
| SLE 11439 | 21 | F | AA | Y | Q | X | X |  |
| SLE 11440 | 45 | F | EA | Y | Q | X | X | X |
| SLE 11445 | 19 | F | AA | Y | Q | X | X |  |
| SLE 11453 | 34 | F | EA | Y | F | X | X |  |
| SLE 11473 | 41 | F | AA | Y | Q | X | X |  |
| SLE 11476 | 46 | F | AA | Y | Q | X | X |  |
| SLE 11530 | 37 | F | EA | Y | F | X | X | X |
| SLE 11546 | 39 | F | EA | Y | Q | X | X |  |
| SLE 11548 | 44 | F | EA | Y | F | X | X | X |
| SLE 11555 | 26 | F | AA | Y | Q | X | X | X |
| SLE 11563 | 24 | M | EA | Y | Q | X | X | X |
| SLE 11585 | 29 | F | AA | Y | F | X | X | X |
| SLE 11586 | 25 | F | EA | Y | F | X | X | X |
| SLE 1167 | 28 | F | AA | Y | F | X | X |  |
| SLE 1182 | 45 | F | AA | Y | Q | X | X | X |
| SLE 1190 | 45 | F | AA | Y | Q | X | X | X |
| SLE 1296 | 41 | F | AA | Y | Q | X | X |  |
| SLE 1627 | 41 | F | AA | Y | F | X | X |  |
| SLE 1796 | 25 | F | AA | Y | Q | X | X |  |
| SLE 2534 | 29 | F | AA | Y | Q | X | X | X |
| SLE 3667 | 30 | F | AA | Y | Q | X | X | X |
| SLE 3686 | 25 | F | EA | Y | Q | X | X | X |
| SLE 3696 | 44 | M | EA | Y | Q | X | X | X |
| SLE 4042 | 37 | M | AA | Y | Q | X | X |  |
| SLE 4046 | 49 | F | AA | Y | F | X | X |  |
| SLE 4048 | 48 | F | AA | Y | Q | X | X | X |
| SLE 4090 | 25 | F | AA | Y | Q | X | X |  |
| SLE 4097 | 30 | M | AA | Y | F | X | X |  |
| SLE 4440 | 22 | F | AA | Y | F | X | X | X |
| SLE 5024 | 28 | M | AA | Y | Q | X | X | X |
| SLE 5274 | 24 | F | AA | Y | Q | X | X |  |
| SLE 5422 | 22 | F | AA | Y | F | X |  | X |
| SLE 5424 | 26 | F | AA | Y | Q | X | X | X |
| SLE 92 | 33 | F | AA | Y | Q | X | X |  |
| 10412 | 33 | F | AA | N |  | X | X |  |
| 10413 | 25 | F | AA | N |  | X | X |  |
| 10414 | 26 | F | AA | N |  | X | X |  |
| 10436 | 22 | M | AA | N |  | X | X |  |
| 10450 | 28 | F | AA | N |  | X | X |  |
| 10576 | 33 | F | AA | N |  | X | X |  |
| 10897 | 27 | F | AA | N |  | X | X |  |
| 10943 | 48 | F | AA | N |  | X | X |  |
| 11016 | 33 | M | EA | N |  | X | X |  |
| 11080 | 27 | F | AA | N |  | X | X |  |
| 11246 | 26 | M | EA | N |  | X | X |  |
| 11301 | 22 | F | AA | N |  | X | X |  |
| 11310 | 35 | M | AA | N |  | X | X |  |
| 11314 | 26 | M | AA | N |  | X | X |  |
| 11369 | 31 | F | AA | N |  | X | X |  |
| 11394 | 21 | F | EA | N |  | X | X |  |
| 11406 | 29 | F | EA | N |  | X | X |  |
| 11423 | 28 | F | EA | N |  | X | X |  |
| 11429 | 24 | M | EA | N |  | X | X |  |
| 11432 | 25 | F | AA | N |  | X | X | X |
| 11436 | 31 | M | EA | N |  | X | X |  |
| 11451 | 24 | F | EA | N |  | X | X |  |
| 11463 | 38 | F | EA | N |  | X | X | X |
| 11471 | 21 | F | EA | N |  | X | X |  |
| 11533 | 28 | F | EA | N |  | X | X | X |
| 11534 | 37 | M | AA | N |  | X | X | X |
| 11538 | 40 | F | AA | N |  | X | X | X |
| 11542 | 36 | F | EA | N |  | X | X | X |
| 11550 | 25 | F | EA | N |  | X | X | X |
| 11556 | 44 | F | EA | N |  | X | X | X |
| 11558 | 32 | F | AA | N |  | X | X | X |
| 11559 | 23 | F | EA | N |  | X | X | X |
| 11560 | 23 | F | AA | N |  | X | X | X |
| 11561 | 31 | F | AA | N |  | X | X | X |
| 11568 | 24 | F | AA | N |  | X | X | X |
| 11570 | 25 | F | AA | N |  | X | X | X |
| 11571 | 37 | F | EA | N |  | X | X | X |
| 11573 | 22 | F | EA | N |  | X | X | X |
| 11575 | 22 | F | AA | N |  | X | X | X |
| 11577 | 25 | F | AA | N |  | X | X | X |
| 11578 | 34 | F | AA | N |  | X | X | X |
| 11579 | 24 | M | AA | N |  | X | X | X |
| 1433 | 30 | M | EA | N |  |  | X | X |
| 1451 | 51 | M | EA | N |  |  | X | X |
| 1501 | 41 | F | AA | N |  | X | X | X |
| 1690 | 65 | F | EA | N |  |  | X | X |
| 3755 | 32 | M | AA | N |  | X | X |  |
| 3968 | 32 | F | AA | N |  | X | X |  |
| 4010 | 45 | F | AA | N |  | X | X |  |
| 4122 | 21 | F | EA | N |  | X | X |  |
| 4136 | 32 | F | AA | N |  | X | X | X |
| 4184 | 34 | F | AA | N |  | X | X |  |
| 4492 | 34 | F | AA | N |  | X | X | X |
| 4665 | 26 | F | AA | N |  | X | X |  |
| 4791 | 38 | F | AA | N |  | X | X |  |
| 4996 | 27 | M | EA | N |  | X | X |  |
| TW | 35 | M | EA | N |  |  |  | X |

Table of all individuals in the primary cohort, their age, gender, ethnicity, disease status, disease activity, and which cell types were successfully analyzed for each individual.
