# Supplementary material for: Genome-Wide DNA Methylation Analysis of Systemic Lupus Erythematosus Reveals Persistent Hypomethylation of Interferon Genes and Compositional Changes to CD4+ T-cell Populations
Source: PLoS Genet. 2013 Aug 8;9(8):e1003678. doi: 10.1371/journal.pgen.1003678 (PMC3738443; doi:10.1371/journal.pgen.1003678)
Supplement: Table S3 — List of CpGs with aberrant methylation in any cell type. Table of CpGs with p-values<1×10^−8^ in T-cells, B-cells or monocytes. The columns for each cell type indicate mean methylation proportion after correction for all covariates in controls/SLE patients. Highly significant effects (p<1×10^−8^) have double asterisks. Mildly significant (FDR<1%) have single asterisks. (DOCX) [file pgen.1003678.s007.docx]

**Table S3. List of CpGs with Aberrant Methylation in Any Cell Type.**

| **CpG** | **Chr** | **Pos** | **Genes** | **IFN** | **CD4** | **CD19** | **CD14** |
| --- | --- | --- | --- | --- | --- | --- | --- |
| cg20062691 | 1 | 949392 | ISG15 | IFN | 0.6564/0.5262 ** | 0.744/0.6047 ** | 0.8137/0.6689 * |
| cg03811829 | 1 | 949449 | ISG15 | IFN | 0.6993/0.6041 * | 0.811/0.7301 ** | 0.8257/0.7156 * |
| cg11211792 | 1 | 949634 | ISG15 | IFN | 0.6879/0.6065 * | 0.8019/0.7234 ** | 0.8332/0.7625 |
| cg04788999 | 1 | 949850 | ISG15 | IFN | 0.5952/0.4748 ** | 0.6705/0.5732 ** | 0.6981/0.5785 * |
| cg16526047 | 1 | 949893 | ISG15 | IFN | 0.4901/0.3854 ** | 0.5689/0.4595 ** | 0.637/0.4974 * |
| cg07123796 | 1 | 950971 |  |  | 0.5326/0.4643 ** | 0.5564/0.5222 | 0.4278/0.3624 * |
| cg09363892 | 1 | 954619 | AGRN |  | 0.1352/0.1531 * | 0.1638/0.2269 ** | 0.1448/0.1997 * |
| cg03185752 | 1 | 954631 | AGRN |  | 0.096/0.1066 | 0.0966/0.1291 ** | 0.0919/0.1236 |
| cg09182189 | 1 | 1709203 | NADK |  | 0.2616/0.2247 ** | 0.1543/0.1331 | 0.2034/0.1723 |
| cg07842327 | 1 | 2174725 | SKI |  | 0.9167/0.9366 ** | 0.9723/0.969 | 0.9765/0.9775 |
| cg08942499 | 1 | 2219932 | SKI |  | 0.4213/0.5 ** | 0.8288/0.8194 | 0.8473/0.8294 |
| cg15605124 | 1 | 3230250 | PRDM16 |  | 0.7854/0.7463 ** | 0.8365/0.8166 | 0.756/0.729 |
| cg21848084 | 1 | 3264381 | PRDM16 |  | 0.3631/0.3963 * | 0.2804/0.353 ** | 0.3122/0.3796 |
| cg08840010 | 1 | 8000314 | TNFRSF9 |  | 0.6102/0.5177 ** | 0.8288/0.778 | 0.1733/0.1752 |
| cg20244489 | 1 | 8209787 |  |  | 0.3751/0.5016 ** | 0.8607/0.8589 | 0.9323/0.9269 |
| cg07145988 | 1 | 8692312 | RERE |  | 0.4837/0.5775 ** | 0.8122/0.833 | 0.957/0.9542 |
| cg20305595 | 1 | 9293833 | H6PD |  | 0.5452/0.4917 ** | 0.6359/0.6068 | 0.322/0.3118 |
| cg06829969 | 1 | 10460205 | PGD |  | 0.7758/0.7129 ** | 0.751/0.7203 | 0.492/0.47 |
| cg17282688 | 1 | 11516947 |  |  | 0.3246/0.4208 ** | 0.7761/0.781 | 0.8759/0.8903 |
| cg21826784 | 1 | 11795937 | AGTRAP |  | 0.5183/0.4536 ** | 0.5131/0.5048 | 0.0417/0.0473 |
| cg25467652 | 1 | 11795976 | AGTRAP |  | 0.3304/0.2768 ** | 0.2864/0.2568 | 0.0457/0.0439 |
| cg03593358 | 1 | 12039985 | MFN2 |  | 0.1683/0.1297 ** | 0.1472/0.1526 | 0.0398/0.0372 |
| cg11217193 | 1 | 12538341 | VPS13D |  | 0.6152/0.6727 ** | 0.7443/0.7482 | 0.7682/0.7747 |
| cg19620994 | 1 | 12774904 | AADACL3 |  | 0.7426/0.6705 ** | 0.8668/0.8435 | 0.4327/0.3871 |
| cg01017257 | 1 | 15059738 | KIAA1026 |  | 0.7629/0.704 ** | 0.7458/0.7036 | 0.6789/0.6347 |
| cg09063663 | 1 | 16484811 |  |  | 0.291/0.3359 ** | 0.4064/0.4196 | 0.5301/0.5312 |
| cg20065005 | 1 | 16712408 | C1orf144 |  | 0.2595/0.3019 ** | 0.4229/0.4358 | 0.4884/0.5075 |
| cg09119494 | 1 | 23851471 | E2F2 |  | 0.063/0.0522 ** | 0.0353/0.0367 | 0.0401/0.0385 |
| cg13977835 | 1 | 23883338 |  |  | 0.4889/0.5856 ** | 0.4783/0.5901 ** | 0.9395/0.9295 |
| cg00171783 | 1 | 23953480 | MDS2 |  | 0.7526/0.8198 ** | 0.783/0.8163 | 0.8703/0.8635 |
| cg18418928 | 1 | 23953778 | MDS2 |  | 0.3694/0.5014 ** | 0.1628/0.2514 * | 0.9786/0.9828 |
| cg15519096 | 1 | 24833311 | RCAN3 |  | 0.3129/0.4068 * | 0.2141/0.3731 ** | 0.9727/0.9821 |
| cg20146241 | 1 | 24861604 | RCAN3 |  | 0.1198/0.1701 ** | 0.0723/0.0849 | 0.3857/0.4411 |
| cg18128887 | 1 | 24861708 | RCAN3 |  | 0.1312/0.1925 ** | 0.0727/0.0933 | 0.447/0.5184 |
| cg15498134 | 1 | 25246854 | RUNX3 |  | 0.5569/0.5359 | 0.8059/0.7112 ** | 0.8985/0.8993 |
| cg16022904 | 1 | 26870173 | RPS6KA1 |  | 0.366/0.3096 ** | 0.0597/0.0762 | 0.0832/0.0918 |
| cg05649922 | 1 | 27029290 | ARID1A |  | 0.1317/0.1658 ** | 0.2473/0.2359 | 0.3353/0.3739 |
| cg17820878 | 1 | 27440463 | SLC9A1 |  | 0.6882/0.7546 ** | 0.9693/0.9693 | 0.9655/0.967 |
| cg13300580 | 1 | 27440539 | SLC9A1 |  | 0.2927/0.3444 ** | 0.7874/0.7708 | 0.6082/0.5921 |
| cg25130381 | 1 | 27440721 | SLC9A1 |  | 0.4617/0.521 ** | 0.8349/0.8305 | 0.6786/0.6892 |
| cg09725874 | 1 | 27480106 | SLC9A1 |  | 0.4995/0.5556 ** | 0.2516/0.2678 | 0.7573/0.7664 |
| cg03604774 | 1 | 27849102 |  |  | 0.1834/0.2735 ** | 0.8828/0.8538 | 0.8281/0.8315 |
| cg12759387 | 1 | 27849177 |  |  | 0.2955/0.3531 ** | 0.7206/0.7034 | 0.6988/0.7096 |
| cg03018771 | 1 | 27940619 | FGR |  | 0.3872/0.4932 ** | 0.7681/0.7656 | 0.8462/0.8524 |
| cg04089743 | 1 | 27977270 |  |  | 0.6268/0.5298 ** | 0.825/0.787 | 0.1715/0.1561 |
| cg12424383 | 1 | 27990967 |  |  | 0.7994/0.7259 ** | 0.5484/0.5592 | 0.7009/0.6214 * |
| cg15459165 | 1 | 31223850 | LAPTM5 |  | 0.1257/0.1697 ** | 0.0376/0.0523 * | 0.3404/0.3873 |
| cg03741348 | 1 | 32712237 | FAM167B |  | 0.6178/0.5648 * | 0.1562/0.2044 ** | 0.3341/0.3583 |
| cg10250177 | 1 | 32739752 | LCK |  | 0.1682/0.2371 ** | 0.0644/0.0781 | 0.5873/0.6159 |
| cg00625963 | 1 | 32740034 | LCK |  | 0.5143/0.5958 ** | 0.8553/0.854 | 0.9717/0.969 |
| cg15482893 | 1 | 32837667 | BSDC1 |  | 0.1606/0.1995 ** | 0.2113/0.2278 | 0.2794/0.309 |
| cg17971578 | 1 | 36852463 | STK40 |  | 0.3536/0.2909 ** | 0.2458/0.2341 | 0.1779/0.1732 |
| cg04430911 | 1 | 36914349 | OSCP1 |  | 0.6971/0.6147 ** | 0.8966/0.8634 | 0.3871/0.3518 |
| cg22483610 | 1 | 37920947 |  |  | 0.1808/0.1501 ** | 0.1245/0.1155 | 0.0344/0.0333 |
| cg17743381 | 1 | 39024825 |  |  | 0.2842/0.3159 ** | 0.3839/0.404 | 0.4084/0.4257 |
| cg14285050 | 1 | 40778294 | COL9A2 |  | 0.2166/0.1763 ** | 0.0706/0.0729 | 0.0599/0.0593 |
| cg01763916 | 1 | 40849202 | SMAP2 |  | 0.6392/0.5396 ** | 0.7715/0.7465 | 0.2824/0.2291 |
| cg14028856 | 1 | 41845592 |  |  | 0.5416/0.4906 ** | 0.2492/0.2579 | 0.2517/0.2439 |
| cg12666727 | 1 | 42128487 | HIVEP3 |  | 0.2869/0.2179 ** | 0.0247/0.0272 | 0.0133/0.0182 |
| cg24254842 | 1 | 42193353 | HIVEP3 |  | 0.2625/0.2007 ** | 0.0421/0.0432 | 0.0393/0.0389 |
| cg12590902 | 1 | 44771985 | ERI3 |  | 0.2366/0.2897 ** | 0.2192/0.2209 | 0.3981/0.4504 |
| cg11448683 | 1 | 45274099 | TCTEX1D4; BTBD19 |  | 0.2317/0.182 ** | 0.2447/0.2338 | 0.052/0.0496 |
| cg06619077 | 1 | 47656003 | PDZK1IP1 |  | 0.6848/0.6271 ** | 0.7465/0.7106 | 0.489/0.4578 |
| cg11399254 | 1 | 47694517 | TAL1 |  | 0.6333/0.5683 ** | 0.5655/0.5211 | 0.4782/0.4241 |
| cg04542977 | 1 | 51982669 | EPS15 |  | 0.4386/0.3721 ** | 0.235/0.2045 | 0.2448/0.2191 |
| cg24741609 | 1 | 54122060 | GLIS1 |  | 0.1578/0.1918 ** | 0.1304/0.1422 | 0.3142/0.3185 |
| cg19379103 | 1 | 54751576 | SSBP3 |  | 0.6459/0.7012 ** | 0.8817/0.8797 | 0.9445/0.9406 |
| cg15687600 | 1 | 59280952 |  |  | 0.211/0.1628 ** | 0.0954/0.0921 | 0.0439/0.0419 |
| cg18231025 | 1 | 59573471 |  |  | 0.7923/0.7142 ** | 0.8856/0.8726 | 0.644/0.5885 |
| cg11842367 | 1 | 78703580 | MGC27382 |  | 0.3587/0.4502 ** | 0.4066/0.4987 * | 0.5367/0.6503 |
| cg13304609 | 1 | 79085162 | IFI44L | IFN | 0.8625/0.6636 ** | 0.8779/0.6748 ** | 0.721/0.3671 ** |
| cg06872964 | 1 | 79085250 | IFI44L | IFN | 0.462/0.2585 ** | 0.5915/0.3136 ** | 0.4975/0.1554 ** |
| cg03607951 | 1 | 79085586 | IFI44L | IFN | 0.5923/0.3264 ** | 0.6037/0.3107 ** | 0.3572/0.1 * |
| cg17980508 | 1 | 79085713 | IFI44L | IFN | 0.5238/0.3053 ** | 0.4951/0.316 ** | 0.2256/0.1069 * |
| cg00855901 | 1 | 79085765 | IFI44L | IFN | 0.3132/0.1541 ** | 0.2848/0.1544 ** | 0.0441/0.0316 |
| cg05696877 | 1 | 79088769 | IFI44L | IFN | 0.5582/0.3247 ** | 0.7181/0.3958 ** | 0.6878/0.2886 ** |
| cg01079652 | 1 | 79118191 | IFI44 | IFN | 0.7437/0.5309 ** | 0.8273/0.6453 ** | 0.7095/0.3486 ** |
| cg17142950 | 1 | 84766782 | SAMD13 |  | 0.3986/0.3406 ** | 0.3516/0.3263 | 0.1684/0.1661 |
| cg01167323 | 1 | 90284686 |  |  | 0.5319/0.6195 ** | 0.8907/0.8821 | 0.9192/0.9201 |
| cg01890417 | 1 | 91488275 | ZNF644 |  | 0.545/0.6604 ** | 0.5549/0.5798 | 0.951/0.951 |
| cg03260624 | 1 | 91970722 | CDC7 |  | 0.8219/0.7626 ** | 0.868/0.844 | 0.7704/0.7141 |
| cg07243548 | 1 | 108231160 | VAV3 |  | 0.6977/0.6257 ** | 0.9286/0.9023 | 0.7054/0.6612 |
| cg03725309 | 1 | 109757585 | SARS |  | 0.3126/0.2678 ** | 0.5258/0.4809 | 0.1842/0.148 |
| cg07706695 | 1 | 111179008 |  |  | 0.2378/0.3261 * | 0.0698/0.1304 ** | 0.8498/0.8543 |
| cg09516523 | 1 | 111769133 | CHI3L2 |  | 0.4586/0.5505 ** | 0.9103/0.8963 | 0.9549/0.9573 |
| cg02590572 | 1 | 111769481 | CHI3L2 |  | 0.4887/0.5732 ** | 0.9455/0.9362 | 0.9934/0.9901 |
| cg14414943 | 1 | 111770718 | CHI3L2 |  | 0.5942/0.6736 ** | 0.9376/0.9199 | 0.9723/0.9675 |
| cg08529825 | 1 | 114489550 | HIPK1 |  | 0.3969/0.4667 ** | 0.7321/0.732 | 0.7753/0.7734 |
| cg00356916 | 1 | 116256618 | CASQ2 |  | 0.6097/0.7002 ** | 0.9135/0.9043 | 0.9542/0.9483 |
| cg17745262 | 1 | 144539026 |  |  | 0.5051/0.5693 ** | 0.8256/0.8037 | 0.8692/0.8603 |
| cg11120551 | 1 | 146713996 | CHD1L |  | 0.6226/0.5469 ** | 0.5445/0.505 | 0.509/0.4652 |
| cg17850088 | 1 | 150119278 |  |  | 0.3887/0.4758 ** | 0.7668/0.7552 | 0.7601/0.7961 |
| cg21262032 | 1 | 154437693 | IL6R |  | 0.3262/0.3976 ** | 0.8772/0.8665 | 0.7619/0.7779 |
| cg24304309 | 1 | 154577895 | ADAR | IFN | 0.5467/0.4916 ** | 0.668/0.5988 | 0.7411/0.6537 * |
| cg26189283 | 1 | 155109378 | RAG1AP1 |  | 0.365/0.2998 ** | 0.3079/0.2462 | 0.4321/0.335 |
| cg21877565 | 1 | 156461550 | MEF2D |  | 0.4227/0.3315 ** | 0.0489/0.0593 | 0.1218/0.1276 |
| cg16374333 | 1 | 157103641 | ETV3 |  | 0.3366/0.4614 ** | 0.9562/0.9412 | 0.9269/0.9269 |
| cg25597580 | 1 | 157964529 | KIRREL |  | 0.5782/0.6596 ** | 0.9242/0.9166 | 0.9581/0.9558 |
| cg11525252 | 1 | 158041312 | KIRREL |  | 0.51/0.6185 ** | 0.9469/0.9446 | 0.9863/0.9892 |
| cg15593510 | 1 | 158369112 | OR10T2 |  | 0.4303/0.5255 ** | 0.8014/0.8114 | 0.9428/0.9534 |
| cg18865207 | 1 | 160765919 | LY9 |  | 0.0777/0.103 ** | 0.0504/0.0596 | 0.2151/0.2591 |
| cg05138397 | 1 | 160768549 | LY9 |  | 0.5904/0.6583 ** | 0.8677/0.8658 | 0.9057/0.9039 |
| cg07106927 | 1 | 160769081 | LY9 |  | 0.6069/0.6957 ** | 0.9046/0.9003 | 0.9665/0.9667 |
| cg17013990 | 1 | 161091682 | DEDD |  | 0.5427/0.4403 ** | 0.1633/0.1887 | 0.0456/0.0435 |
| cg00160981 | 1 | 161691911 | FCRLB |  | 0.2642/0.2088 ** | 0.3178/0.3377 | 0.0727/0.0722 |
| cg09554443 | 1 | 167487762 | CD247 |  | 0.1525/0.2179 ** | 0.1958/0.2149 | 0.4557/0.5206 |
| cg03945538 | 1 | 173447427 | PRDX6 |  | 0.6007/0.5075 * | 0.3988/0.3436 ** | 0.1797/0.1398 |
| cg17267239 | 1 | 173640200 | ANKRD45 |  | 0.4655/0.4021 ** | 0.5563/0.4706 ** | 0.2168/0.1929 |
| cg02482603 | 1 | 174843754 | RABGAP1L |  | 0.3844/0.3041 ** | 0.2167/0.1803 * | 0.2602/0.2078 |
| cg05702218 | 1 | 174843909 | RABGAP1L |  | 0.3423/0.2691 ** | 0.2076/0.1754 | 0.1925/0.1706 |
| cg13130398 | 1 | 174844397 | RABGAP1L |  | 0.8388/0.7189 ** | 0.7722/0.6008 ** | 0.853/0.6473 ** |
| cg07285983 | 1 | 174844490 | RABGAP1L |  | 0.7489/0.6105 ** | 0.7092/0.4618 ** | 0.8035/0.5472 ** |
| cg03471346 | 1 | 179112077 | ABL2 |  | 0.418/0.5256 ** | 0.8143/0.8066 | 0.8485/0.8494 |
| cg12593541 | 1 | 181110845 |  |  | 0.0712/0.0575 ** | 0.0702/0.0619 | 0.0272/0.0259 |
| cg05937055 | 1 | 181128764 |  |  | 0.1576/0.2084 ** | 0.0986/0.116 | 0.3613/0.3767 |
| cg07504763 | 1 | 198575077 |  |  | 0.6632/0.5872 ** | 0.5809/0.5426 | 0.4195/0.3493 |
| cg08272268 | 1 | 200380059 | ZNF281 |  | 0.6861/0.6261 ** | 0.5535/0.5339 | 0.6577/0.6365 |
| cg01798157 | 1 | 203276595 | BTG2 |  | 0.0957/0.1461 ** | 0.0498/0.063 | 0.3184/0.392 |
| cg20867633 | 1 | 204183116 | GOLT1A |  | 0.0757/0.0591 ** | 0.0362/0.0359 | 0.0341/0.0311 |
| cg20240347 | 1 | 204465584 |  |  | 0.3374/0.4232 ** | 0.4763/0.4834 | 0.8555/0.8303 |
| cg19638572 | 1 | 206733139 | RASSF5 |  | 0.1426/0.1999 ** | 0.0816/0.0937 | 0.6081/0.6262 |
| cg23088126 | 1 | 207078912 | FAIM3 |  | 0.4368/0.5116 ** | 0.3206/0.343 | 0.8764/0.8775 |
| cg17460386 | 1 | 207095668 | FAIM3 |  | 0.1274/0.1682 ** | 0.1113/0.1402 * | 0.3531/0.3869 |
| cg18234296 | 1 | 210407896 | C1orf133; SERTAD4 |  | 0.4884/0.558 ** | 0.7747/0.7759 | 0.8482/0.8562 |
| cg27433482 | 1 | 211498962 | TRAF5 |  | 0.5563/0.6149 ** | 0.6652/0.678 | 0.8533/0.8678 |
| cg07107916 | 1 | 212457424 | PPP2R5A |  | 0.3287/0.4309 ** | 0.9105/0.9036 | 0.9107/0.9193 |
| cg06093152 | 1 | 212662017 |  |  | 0.6026/0.5086 ** | 0.769/0.7203 | 0.4016/0.2955 |
| cg15009294 | 1 | 214813712 | CENPF |  | 0.4602/0.5825 ** | 0.915/0.901 | 0.9045/0.9022 |
| cg26221105 | 1 | 223914343 | CAPN2 |  | 0.0924/0.0729 ** | 0.0795/0.0762 | 0.0326/0.0314 |
| cg26341831 | 1 | 226036279 | TMEM63A |  | 0.1153/0.1526 ** | 0.0422/0.0606 * | 0.2777/0.3581 |
| cg21837189 | 1 | 226085836 |  |  | 0.3182/0.3781 * | 0.1591/0.23 ** | 0.7695/0.7555 |
| cg12221970 | 1 | 226884832 | ITPKB |  | 0.7193/0.7768 ** | 0.7375/0.7572 | 0.9803/0.9835 |
| cg13420364 | 1 | 234857659 |  |  | 0.4497/0.3812 ** | 0.3666/0.3204 * | 0.4475/0.3843 |
| cg00851028 | 1 | 234905772 |  |  | 0.4155/0.4955 ** | 0.8498/0.8316 | 0.7954/0.8053 |
| cg05926640 | 1 | 235091187 |  |  | 0.2928/0.2411 ** | 0.0827/0.073 | 0.0459/0.0418 |
| cg07367601 | 1 | 235114342 |  |  | 0.3069/0.2385 ** | 0.0498/0.0512 | 0.0535/0.0498 |
| cg00348992 | 1 | 245365165 | KIF26B |  | 0.0793/0.1012 ** | 0.0817/0.0797 | 0.0747/0.0869 |
| cg17855595 | 1 | 249148077 | ZNF692 |  | 0.3878/0.4585 ** | 0.6602/0.6805 | 0.6928/0.6827 |
| cg01028142 | 2 | 7004578 | CMPK2 |  | 0.7915/0.5247 ** | 0.8164/0.5701 ** | 0.7155/0.3683 ** |
| cg24935042 | 2 | 7006627 | CMPK2 |  | 0.0649/0.0355 ** | 0.0443/0.0337 * | 0.0285/0.0222 |
| cg23213327 | 2 | 7016509 | RSAD2 |  | 0.6145/0.5015 * | 0.3183/0.2127 ** | 0.1211/0.0861 |
| cg15346781 | 2 | 7017571 | RSAD2 |  | 0.2693/0.2109 ** | 0.2999/0.2344 * | 0.1976/0.1482 |
| cg10959651 | 2 | 7018020 | RSAD2 |  | 0.1878/0.114 ** | 0.3728/0.2302 ** | 0.2625/0.1151 * |
| cg10549986 | 2 | 7018153 | RSAD2 |  | 0.1694/0.0773 ** | 0.4787/0.2439 ** | 0.1551/0.0377 |
| cg04618171 | 2 | 10470465 | HPCAL1 |  | 0.6084/0.7129 ** | 0.9425/0.9293 | 0.924/0.933 |
| cg00141688 | 2 | 10517352 | HPCAL1 |  | 0.7416/0.8102 ** | 0.8566/0.8538 | 0.8938/0.9044 |
| cg06200244 | 2 | 12862337 | TRIB2 |  | 0.3261/0.4151 ** | 0.8769/0.8706 | 0.8738/0.8617 |
| cg08569786 | 2 | 23616768 | KLHL29 |  | 0.3337/0.4069 ** | 0.6474/0.6331 | 0.6441/0.652 |
| cg00050692 | 2 | 25524877 | DNMT3A |  | 0.3941/0.3212 ** | 0.3988/0.3686 | 0.0902/0.0922 |
| cg08364093 | 2 | 28114164 | LOC100302650; RBKS; BRE |  | 0.1706/0.139 ** | 0.1326/0.1331 | 0.0584/0.0565 |
| cg15078838 | 2 | 28114349 | LOC100302650; RBKS; BRE |  | 0.374/0.2928 ** | 0.333/0.3252 | 0.0331/0.0383 |
| cg21200667 | 2 | 30628085 |  |  | 0.5362/0.6093 ** | 0.9113/0.9049 | 0.9311/0.9337 |
| cg24523650 | 2 | 37381088 | EIF2AK2 | IFN | 0.5323/0.4428 ** | 0.6237/0.5952 | 0.5284/0.4417 |
| cg17326313 | 2 | 37383568 | EIF2AK2 | IFN | 0.1693/0.0737 ** | 0.0929/0.0585 ** | 0.0355/0.0341 |
| cg16795804 | 2 | 37384523 | EIF2AK2 | IFN | 0.3261/0.2502 ** | 0.2429/0.2114 | 0.2382/0.1976 |
| cg14126601 | 2 | 37384708 | EIF2AK2 | IFN | 0.552/0.4173 ** | 0.2895/0.1881 ** | 0.4918/0.4091 * |
| cg00841141 | 2 | 37416819 | SULT6B1 |  | 0.7971/0.7412 ** | 0.864/0.8441 | 0.6522/0.5904 |
| cg05627557 | 2 | 37418009 |  |  | 0.8394/0.784 ** | 0.898/0.8665 | 0.6695/0.5959 |
| cg15657390 | 2 | 38155843 | FAM82A1 |  | 0.4945/0.5647 ** | 0.6079/0.6172 | 0.8581/0.8659 |
| cg01644640 | 2 | 40147801 |  |  | 0.3565/0.4635 ** | 0.0567/0.0686 | 0.8668/0.8699 |
| cg00484711 | 2 | 42331922 |  |  | 0.353/0.4615 ** | 0.5126/0.5293 | 0.9152/0.8898 |
| cg10588617 | 2 | 43027940 |  |  | 0.1881/0.2502 ** | 0.4654/0.4895 | 0.3952/0.4888 |
| cg09988805 | 2 | 43278552 |  |  | 0.2118/0.301 ** | 0.488/0.4597 | 0.8247/0.8128 |
| cg09999348 | 2 | 46398848 | PRKCE |  | 0.6064/0.6824 ** | 0.9133/0.9125 | 0.9618/0.9566 |
| cg17411016 | 2 | 47100912 |  |  | 0.4266/0.3586 ** | 0.6573/0.6531 | 0.1036/0.1029 |
| cg00883689 | 2 | 54802904 | SPTBN1 |  | 0.4521/0.3735 ** | 0.8917/0.8657 | 0.2604/0.2545 |
| cg23598480 | 2 | 60102800 |  |  | 0.2014/0.2471 ** | 0.5053/0.5243 | 0.4522/0.4694 |
| cg17253709 | 2 | 62442007 | B3GNT2 |  | 0.4862/0.412 ** | 0.6979/0.6572 | 0.2158/0.2169 |
| cg25521400 | 2 | 62445279 | B3GNT2 |  | 0.6011/0.4848 ** | 0.8082/0.7993 | 0.0527/0.0514 |
| cg12627844 | 2 | 64245000 | VPS54 |  | 0.5782/0.5063 ** | 0.7229/0.6454 ** | 0.5106/0.4442 |
| cg26164488 | 2 | 64440295 |  |  | 0.6019/0.4978 ** | 0.5745/0.5308 | 0.2571/0.218 |
| cg22737154 | 2 | 64631614 |  |  | 0.6913/0.6156 ** | 0.704/0.6389 * | 0.4265/0.3667 |
| cg14209730 | 2 | 64632636 |  |  | 0.8167/0.7464 ** | 0.9307/0.9069 * | 0.558/0.4955 |
| cg25413977 | 2 | 66651619 |  |  | 0.7076/0.6423 ** | 0.6912/0.6433 | 0.743/0.6636 |
| cg25420477 | 2 | 70319121 |  |  | 0.3316/0.4262 ** | 0.9262/0.9156 | 0.929/0.9322 |
| cg02956499 | 2 | 74280825 | TET3 |  | 0.5393/0.6198 ** | 0.7783/0.7891 | 0.9716/0.9671 |
| cg12393275 | 2 | 79312224 | REG1B |  | 0.9564/0.956 | 0.4149/0.5503 ** | 0.9369/0.9352 |
| cg17512187 | 2 | 85082845 | C2orf89 |  | 0.237/0.2914 ** | 0.4733/0.4724 | 0.5802/0.5713 |
| cg22242842 | 2 | 85167149 |  |  | 0.2683/0.2155 ** | 0.2222/0.2213 | 0.1107/0.1173 |
| cg00533827 | 2 | 86163827 |  |  | 0.8727/0.8374 ** | 0.9198/0.9044 | 0.7701/0.7372 |
| cg26155674 | 2 | 86793873 |  |  | 0.5181/0.5939 ** | 0.8248/0.8218 | 0.9195/0.9195 |
| cg11714752 | 2 | 96926568 | TMEM127 |  | 0.2592/0.2265 | 0.085/0.1075 ** | 0.0393/0.036 |
| cg16469046 | 2 | 99062999 | INPP4A |  | 0.3156/0.4406 ** | 0.8834/0.8767 | 0.9063/0.9133 |
| cg11555067 | 2 | 99081350 | INPP4A |  | 0.2715/0.336 ** | 0.6463/0.5911 | 0.5703/0.5419 |
| cg20923498 | 2 | 99096920 | INPP4A |  | 0.3973/0.4767 ** | 0.8596/0.8541 | 0.8964/0.8741 |
| cg25015733 | 2 | 99342986 | MGAT4A |  | 0.6152/0.7161 ** | 0.9276/0.9223 | 0.956/0.9518 |
| cg23527387 | 2 | 100056660 | REV1 |  | 0.8719/0.8164 ** | 0.8777/0.848 | 0.8706/0.818 |
| cg10523140 | 2 | 101768941 | TBC1D8 |  | 0.2856/0.244 ** | 0.1834/0.1741 | 0.0763/0.0751 |
| cg06770878 | 2 | 105372265 |  |  | 0.6204/0.54 ** | 0.7307/0.7052 | 0.3312/0.3533 |
| cg01124420 | 2 | 109605518 | EDAR |  | 0.2365/0.2998 ** | 0.5158/0.5233 | 0.6038/0.6111 |
| cg02543462 | 2 | 113885116 | IL1RN |  | 0.3683/0.2899 ** | 0.5728/0.5049 | 0.0437/0.0421 |
| cg03989987 | 2 | 113885277 | IL1RN |  | 0.4059/0.3211 ** | 0.6869/0.636 | 0.0243/0.0231 |
| cg25265126 | 2 | 113891507 | IL1RN |  | 0.5451/0.5971 ** | 0.8179/0.8145 | 0.8488/0.85 |
| cg24750513 | 2 | 127819455 | BIN1 |  | 0.8189/0.7597 ** | 0.7978/0.7511 * | 0.6421/0.5925 |
| cg06021088 | 2 | 127822551 | BIN1 |  | 0.3308/0.2745 ** | 0.2844/0.2631 | 0.0402/0.0413 |
| cg23229770 | 2 | 129491004 |  |  | 0.6476/0.55 ** | 0.6829/0.6453 | 0.0607/0.0536 |
| cg16200531 | 2 | 135532566 |  |  | 0.3029/0.3613 ** | 0.729/0.7214 | 0.4189/0.4429 |
| cg00602811 | 2 | 145278564 | ZEB2 |  | 0.5257/0.4263 * | 0.6333/0.5221 ** | 0.1465/0.1377 |
| cg12940181 | 2 | 145353012 |  |  | 0.6056/0.4852 ** | 0.8808/0.8188 * | 0.0403/0.0373 |
| cg16682903 | 2 | 158694670 | ACVR1 |  | 0.7088/0.6309 ** | 0.7317/0.693 | 0.5332/0.4655 |
| cg05874176 | 2 | 172019979 | TLK1 |  | 0.0663/0.066 | 0.2168/0.2698 ** | 0.1083/0.1035 |
| cg11119767 | 2 | 174024669 | ZAK |  | 0.6765/0.5729 ** | 0.856/0.8418 | 0.044/0.0385 |
| cg14951497 | 2 | 191875807 | STAT1 | IFN | 0.5272/0.4356 * | 0.9181/0.8385 ** | 0.9397/0.8747 |
| cg00676801 | 2 | 191876673 | STAT1 | IFN | 0.4402/0.3895 | 0.8641/0.7269 ** | 0.9476/0.8724 |
| cg03110996 | 2 | 191883483 |  |  | 0.5776/0.4865 ** | 0.6776/0.5937 * | 0.7654/0.7153 |
| cg15636519 | 2 | 191894418 | STAT4 | IFN | 0.1595/0.1491 | 0.4738/0.363 ** | 0.2795/0.1727 * |
| cg08153883 | 2 | 201170809 | SPATS2L |  | 0.2516/0.1874 ** | 0.0907/0.0851 | 0.0577/0.0603 |
| cg14106933 | 2 | 201194453 | SPATS2L |  | 0.6146/0.6927 ** | 0.466/0.4887 | 0.8632/0.8688 |
| cg06226703 | 2 | 201242945 | SPATS2L |  | 0.8739/0.7912 ** | 0.8403/0.7799 * | 0.919/0.9091 |
| cg21443659 | 2 | 217745721 |  |  | 0.5164/0.4335 ** | 0.5555/0.4709 * | 0.4855/0.3487 * |
| cg19412109 | 2 | 220082807 | ABCB6 |  | 0.2384/0.2042 ** | 0.0997/0.0939 | 0.042/0.0391 |
| cg10835083 | 2 | 222395101 | EPHA4 |  | 0.3715/0.4741 ** | 0.7694/0.784 | 0.8666/0.8998 |
| cg02225786 | 2 | 224808252 | WDFY1 |  | 0.8382/0.7958 ** | 0.8228/0.7744 | 0.8821/0.8821 |
| cg17607231 | 2 | 231090329 | SP140 |  | 0.1736/0.1229 ** | 0.0437/0.0491 | 0.1158/0.0812 |
| cg17147909 | 2 | 231277705 |  |  | 0.7108/0.6665 ** | 0.7853/0.7619 | 0.6895/0.6743 |
| cg23539753 | 2 | 231280698 | SP100 | IFN | 0.0601/0.0506 ** | 0.0469/0.0447 | 0.0373/0.0371 |
| cg06096336 | 2 | 231989800 | PSMD1; HTR2B |  | 0.243/0.3387 ** | 0.6846/0.6926 | 0.6662/0.7256 |
| cg10318368 | 2 | 232507774 |  |  | 0.1134/0.0855 ** | 0.0484/0.0498 | 0.0495/0.0478 |
| cg17490196 | 2 | 232507842 |  |  | 0.5097/0.4287 ** | 0.1472/0.1234 | 0.169/0.1421 |
| cg20793665 | 2 | 232549224 |  |  | 0.5225/0.5964 ** | 0.3316/0.3501 | 0.8944/0.9159 |
| cg05208178 | 2 | 233928423 | INPP5D |  | 0.1472/0.1907 ** | 0.0548/0.0713 * | 0.3674/0.392 |
| cg16000520 | 2 | 235408237 |  |  | 0.6832/0.7839 ** | 0.9355/0.9328 | 0.9697/0.961 |
| cg18141622 | 2 | 238525524 |  |  | 0.5849/0.49 ** | 0.1777/0.1549 | 0.141/0.1243 |
| cg05879734 | 2 | 238850757 |  |  | 0.0856/0.107 ** | 0.1988/0.2005 | 0.1143/0.1159 |
| cg19324997 | 2 | 240169161 | HDAC4 |  | 0.7233/0.7877 ** | 0.9801/0.9769 | 0.9791/0.978 |
| cg26495711 | 2 | 240169280 | HDAC4 |  | 0.4586/0.5249 ** | 0.7481/0.736 | 0.8904/0.8945 |
| cg00144180 | 2 | 240294362 | HDAC4 |  | 0.5556/0.6239 ** | 0.9585/0.9488 | 0.9609/0.9642 |
| cg16967583 | 2 | 241807859 | AGXT |  | 0.5402/0.4624 ** | 0.7769/0.7512 | 0.1127/0.1032 |
| cg19789753 | 2 | 242805962 |  |  | 0.1033/0.0808 ** | 0.065/0.061 | 0.0393/0.0374 |
| cg27217350 | 2 | 242813634 | C2orf85 |  | 0.447/0.5122 ** | 0.0674/0.0933 | 0.7384/0.7429 |
| cg14766620 | 3 | 5018860 |  |  | 0.3229/0.2876 | 0.2712/0.2139 ** | 0.0319/0.0287 |
| cg26269881 | 3 | 5023310 | BHLHE40 |  | 0.4711/0.3626 ** | 0.5386/0.4867 | 0.0667/0.0646 |
| cg20916523 | 3 | 10184584 | VHL |  | 0.3168/0.4208 ** | 0.5885/0.6122 | 0.7953/0.8151 |
| cg21387009 | 3 | 10280255 | IRAK2 |  | 0.3479/0.4423 ** | 0.7743/0.7517 | 0.7966/0.8216 |
| cg00506299 | 3 | 16469127 | RFTN1 |  | 0.7578/0.6703 ** | 0.9028/0.8937 | 0.4383/0.4099 |
| cg21902966 | 3 | 18391029 | SATB1 |  | 0.6174/0.6996 ** | 0.8978/0.889 | 0.9303/0.9291 |
| cg01373248 | 3 | 18480297 | SATB1 |  | 0.1423/0.2013 ** | 0.0438/0.0555 | 0.4088/0.4565 |
| cg04916416 | 3 | 30651317 | TGFBR2 |  | 0.4741/0.593 ** | 0.6999/0.7091 | 0.9719/0.9665 |
| cg19615017 | 3 | 30673459 | TGFBR2 |  | 0.3172/0.4077 ** | 0.6547/0.6469 | 0.7646/0.7982 |
| cg15171154 | 3 | 30722557 | TGFBR2 |  | 0.6338/0.7253 ** | 0.9201/0.9216 | 0.9781/0.9723 |
| cg17980786 | 3 | 32933637 | TRIM71 |  | 0.3731/0.4377 ** | 0.7638/0.7657 | 0.606/0.6303 |
| cg25780219 | 3 | 35785041 | ARPP-21; MIR128-2 |  | 0.7307/0.7839 ** | 0.9278/0.9128 | 0.9369/0.9231 |
| cg21113478 | 3 | 36782467 | DCLK3 |  | 0.2803/0.3568 ** | 0.6434/0.6537 | 0.6572/0.6711 |
| cg00252934 | 3 | 36782736 | DCLK3 |  | 0.3885/0.4733 ** | 0.8852/0.8789 | 0.9491/0.9443 |
| cg23654821 | 3 | 39188656 | CSRNP1 |  | 0.1013/0.082 ** | 0.0908/0.0804 | 0.0481/0.0473 |
| cg03341377 | 3 | 39309355 | CX3CR1 |  | 0.3136/0.3935 ** | 0.7962/0.7674 | 0.7247/0.7886 |
| cg05461503 | 3 | 45837170 | SLC6A20 |  | 0.3839/0.4688 ** | 0.6455/0.6536 | 0.7194/0.7355 |
| cg24940967 | 3 | 45837197 | SLC6A20 |  | 0.253/0.3373 ** | 0.5529/0.5598 | 0.4917/0.5304 |
| cg20496896 | 3 | 46579532 | LRRC2 |  | 0.3832/0.4679 ** | 0.1385/0.1435 | 0.8841/0.8815 |
| cg07220903 | 3 | 46973284 | CCDC12 |  | 0.4611/0.3624 ** | 0.3655/0.3539 | 0.0452/0.0464 |
| cg04625862 | 3 | 48341880 | NME6 |  | 0.1149/0.1592 ** | 0.1942/0.2328 | 0.3246/0.3369 |
| cg01870865 | 3 | 48507087 | TREX1 |  | 0.5259/0.4114 ** | 0.4526/0.4453 | 0.0241/0.0195 |
| cg21788755 | 3 | 48508391 | TREX1 |  | 0.2486/0.2537 | 0.4267/0.3843 | 0.5592/0.4809 ** |
| cg14494596 | 3 | 48542040 | SHISA5 |  | 0.2902/0.2426 ** | 0.1798/0.1702 | 0.1379/0.1226 |
| cg21800196 | 3 | 48673931 | SLC26A6; CELSR3 |  | 0.6791/0.6127 ** | 0.667/0.6314 | 0.4947/0.4573 |
| cg24296397 | 3 | 49692537 | BSN |  | 0.2887/0.3471 ** | 0.1329/0.1698 * | 0.6456/0.7064 |
| cg19381811 | 3 | 49851713 | UBA7 | IFN | 0.3938/0.3297 ** | 0.3233/0.2831 | 0.1408/0.1285 |
| cg01932734 | 3 | 50376409 | RASSF1 |  | 0.2055/0.1605 ** | 0.1734/0.1747 | 0.0412/0.0387 |
| cg05548488 | 3 | 50658472 | MAPKAPK3 |  | 0.1035/0.0832 ** | 0.0908/0.0809 | 0.0231/0.0217 |
| cg07615364 | 3 | 52088726 | DUSP7 |  | 0.2028/0.2576 ** | 0.4383/0.4242 | 0.4223/0.4214 |
| cg25236791 | 3 | 52302252 | WDR82 |  | 0.4232/0.5148 ** | 0.9027/0.8935 | 0.9001/0.8946 |
| cg22117188 | 3 | 52347940 |  |  | 0.5014/0.4138 ** | 0.0802/0.1067 | 0.1738/0.1912 |
| cg15417641 | 3 | 53700141 | CACNA1D |  | 0.7332/0.6505 ** | 0.797/0.7137 * | 0.5892/0.5005 |
| cg21188533 | 3 | 53700263 | CACNA1D |  | 0.7109/0.6099 ** | 0.7278/0.6426 | 0.4997/0.3749 |
| cg18482892 | 3 | 56833426 | ARHGEF3 |  | 0.0986/0.1074 | 0.2701/0.2137 ** | 0.3435/0.2983 |
| cg25799109 | 3 | 57102900 | ARHGEF3; SPATA12 |  | 0.6429/0.5594 ** | 0.1416/0.1304 | 0.3995/0.3412 |
| cg02923224 | 3 | 60133085 | FHIT |  | 0.3959/0.5231 ** | 0.8658/0.8651 | 0.9069/0.9316 |
| cg08992499 | 3 | 63956514 | ATXN7 |  | 0.548/0.6516 ** | 0.8785/0.8747 | 0.9346/0.9329 |
| cg25481160 | 3 | 71111489 | FOXP1 |  | 0.4992/0.5703 ** | 0.6561/0.6742 | 0.7397/0.7426 |
| cg06691963 | 3 | 71149599 | FOXP1 |  | 0.8241/0.8643 ** | 0.8717/0.872 | 0.9659/0.9671 |
| cg24969820 | 3 | 98497864 | ST3GAL6 |  | 0.3586/0.315 ** | 0.3014/0.2934 | 0.0399/0.0375 |
| cg03416645 | 3 | 105087206 | ALCAM |  | 0.0834/0.0625 ** | 0.0452/0.0465 | 0.0423/0.0428 |
| cg02487233 | 3 | 107810687 | CD47 |  | 0.5888/0.6785 ** | 0.8784/0.8761 | 0.9098/0.9113 |
| cg00492070 | 3 | 107810716 | CD47 |  | 0.5981/0.6977 ** | 0.9118/0.9134 | 0.9211/0.9166 |
| cg19400179 | 3 | 108321607 | DZIP3 |  | 0.1413/0.2203 ** | 0.155/0.203 | 0.412/0.4335 |
| cg05894970 | 3 | 119041204 | CDGAP |  | 0.42/0.3381 ** | 0.1262/0.1047 * | 0.062/0.0573 |
| cg11099041 | 3 | 119041385 | CDGAP |  | 0.0515/0.0343 ** | 0.0154/0.0168 | 0.018/0.0175 |
| cg14535274 | 3 | 121151186 | POLQ |  | 0.0655/0.088 ** | 0.0403/0.0424 | 0.176/0.1994 |
| cg22930808 | 3 | 122281881 | DTX3L; PARP9 |  | 0.6826/0.3521 ** | 0.71/0.3511 ** | 0.5913/0.1628 ** |
| cg08122652 | 3 | 122281939 | DTX3L; PARP9 |  | 0.6809/0.3991 ** | 0.6242/0.3325 ** | 0.7143/0.3031 ** |
| cg00959259 | 3 | 122281975 | DTX3L; PARP9 |  | 0.5046/0.2325 ** | 0.3983/0.1833 ** | 0.4932/0.1619 ** |
| cg01948202 | 3 | 122400474 | PARP14 |  | 0.2674/0.1452 ** | 0.1914/0.0915 ** | 0.2144/0.0826 ** |
| cg01721555 | 3 | 122401300 | PARP14 |  | 0.8606/0.7749 ** | 0.5517/0.3954 ** | 0.8956/0.8188 * |
| cg14750551 | 3 | 122401343 | PARP14 |  | 0.9079/0.7801 ** | 0.5038/0.3602 ** | 0.874/0.7343 * |
| cg26313511 | 3 | 125053815 | ZNF148 |  | 0.6352/0.521 ** | 0.8657/0.8136 * | 0.2406/0.2098 |
| cg00867018 | 3 | 128268051 |  |  | 0.6882/0.5995 ** | 0.6446/0.6015 | 0.4501/0.3866 |
| cg07140459 | 3 | 142165032 | XRN1 |  | 0.1287/0.0964 ** | 0.1999/0.1541 * | 0.0608/0.0578 |
| cg18686270 | 3 | 146258875 | PLSCR1 |  | 0.8097/0.6877 ** | 0.6747/0.4638 ** | 0.9206/0.8479 |
| cg06981309 | 3 | 146260954 | PLSCR1 |  | 0.5292/0.2724 ** | 0.5524/0.2467 ** | 0.3738/0.1068 ** |
| cg26283496 | 3 | 146269126 |  |  | 0.4855/0.4132 ** | 0.6506/0.6229 | 0.4861/0.4404 |
| cg07578772 | 3 | 150420821 | FAM194A |  | 0.3231/0.4326 ** | 0.3798/0.4296 | 0.9328/0.9468 |
| cg01044025 | 3 | 151613372 |  |  | 0.2532/0.3257 ** | 0.7436/0.7519 | 0.4222/0.4702 |
| cg13702222 | 3 | 152017240 | MBNL1 |  | 0.2151/0.3045 ** | 0.6279/0.6277 | 0.613/0.6509 |
| cg14969094 | 3 | 156848003 |  |  | 0.2039/0.2693 ** | 0.2907/0.3027 | 0.3638/0.4059 |
| cg25857569 | 3 | 160717237 | PPM1L |  | 0.5832/0.6787 ** | 0.8261/0.8382 | 0.9353/0.9341 |
| cg14612335 | 3 | 170074131 | SKIL |  | 0.1992/0.1415 ** | 0.0199/0.0219 | 0.0217/0.02 |
| cg22611980 | 3 | 177063402 |  |  | 0.1633/0.1241 ** | 0.1261/0.1124 | 0.067/0.056 |
| cg02351277 | 3 | 177397780 |  |  | 0.3996/0.3242 ** | 0.2909/0.2689 | 0.0467/0.0418 |
| cg07266910 | 3 | 178745575 | ZMAT3 |  | 0.3483/0.4322 ** | 0.6937/0.7071 | 0.6367/0.6741 |
| cg04400533 | 3 | 185788716 | ETV5 |  | 0.4691/0.5438 ** | 0.3121/0.351 | 0.7026/0.7433 |
| cg03206681 | 3 | 193512175 |  |  | 0.2917/0.2389 ** | 0.3271/0.2852 | 0.0929/0.0969 |
| cg09709457 | 3 | 194875295 | C3orf21 |  | 0.5569/0.4724 ** | 0.199/0.1916 | 0.1804/0.1935 |
| cg25586848 | 3 | 194875931 | C3orf21 |  | 0.1269/0.093 ** | 0.0318/0.0337 | 0.0403/0.0387 |
| cg23548201 | 3 | 195623792 | TNK2 |  | 0.729/0.6247 ** | 0.7721/0.7048 ** | 0.2811/0.2293 |
| cg15065340 | 3 | 195632915 | TNK2 |  | 0.4455/0.2713 ** | 0.5725/0.45 * | 0.182/0.1055 |
| cg10133462 | 4 | 1305113 | MAEA |  | 0.4043/0.4786 ** | 0.7755/0.7998 | 0.8795/0.8716 |
| cg02035018 | 4 | 2299988 | ZFYVE28 |  | 0.6005/0.6734 ** | 0.6366/0.6483 | 0.8417/0.8385 |
| cg17276535 | 4 | 3372123 | RGS12 |  | 0.2151/0.2687 ** | 0.538/0.5409 | 0.4757/0.4776 |
| cg03132824 | 4 | 3373006 | RGS12 |  | 0.5019/0.587 ** | 0.8425/0.8331 | 0.7886/0.779 |
| cg26852894 | 4 | 11157341 |  |  | 0.5632/0.6663 ** | 0.8823/0.8874 | 0.9394/0.9303 |
| cg07809027 | 4 | 15007205 | CPEB2 |  | 0.6352/0.5309 ** | 0.4857/0.4048 * | 0.5535/0.4483 * |
| cg00598235 | 4 | 17580680 | LAP3 |  | 0.7477/0.6677 ** | 0.6998/0.6149 * | 0.8177/0.7286 * |
| cg26163153 | 4 | 40197912 | RHOH |  | 0.436/0.5482 ** | 0.1691/0.1862 | 0.8957/0.8929 |
| cg20171453 | 4 | 40198129 | RHOH |  | 0.4239/0.5249 * | 0.1012/0.1596 ** | 0.9065/0.8932 |
| cg06970472 | 4 | 40910981 | APBB2 |  | 0.2954/0.2357 ** | 0.3182/0.2784 | 0.1693/0.1371 |
| cg26542660 | 4 | 56813860 | CEP135 |  | 0.1774/0.1326 ** | 0.0457/0.0475 | 0.0508/0.049 |
| cg13468041 | 4 | 74902951 | CXCL3 |  | 0.5239/0.5009 | 0.087/0.1178 ** | 0.3312/0.3507 |
| cg21446955 | 4 | 86851425 | ARHGAP24 |  | 0.0778/0.0989 ** | 0.1682/0.1443 | 0.0689/0.0657 |
| cg02262553 | 4 | 87849250 |  |  | 0.4821/0.3884 ** | 0.8309/0.7717 | 0.0343/0.0322 |
| cg05850997 | 4 | 89207307 | PPM1K |  | 0.8259/0.7833 * | 0.7236/0.6165 ** | 0.8529/0.8429 |
| cg09168222 | 4 | 89299733 | HERC6 |  | 0.8199/0.7615 ** | 0.7761/0.732 | 0.7536/0.6975 |
| cg08750951 | 4 | 89378894 | HERC5 |  | 0.0474/0.0236 * | 0.0585/0.0274 ** | 0.0216/0.0171 |
| cg02215171 | 4 | 89379156 | HERC5 |  | 0.4054/0.2926 ** | 0.4097/0.3049 ** | 0.2159/0.149 * |
| cg15252243 | 4 | 90031538 |  |  | 0.7017/0.7516 ** | 0.8977/0.8964 | 0.91/0.9048 |
| cg08979352 | 4 | 99580530 | TSPAN5 |  | 0.6449/0.5384 ** | 0.5774/0.5184 | 0.4394/0.3891 |
| cg25663524 | 4 | 103489295 | NFKB1 |  | 0.7785/0.8414 ** | 0.9251/0.912 | 0.9452/0.9522 |
| cg14459011 | 4 | 103998497 | NHEDC2 |  | 0.5604/0.4852 ** | 0.4324/0.4088 | 0.3037/0.2603 |
| cg01150799 | 4 | 108955838 | HADH |  | 0.2795/0.3465 ** | 0.1452/0.1556 | 0.699/0.719 |
| cg12623364 | 4 | 109038130 | LEF1 |  | 0.0436/0.0583 ** | 0.0457/0.0621 * | 0.1323/0.1539 |
| cg27190654 | 4 | 109387521 |  |  | 0.4492/0.5121 ** | 0.5799/0.5656 | 0.7744/0.7558 |
| cg09451574 | 4 | 113069076 | C4orf32 |  | 0.7425/0.6503 ** | 0.4786/0.4476 | 0.5651/0.4882 |
| cg24124145 | 4 | 139936112 | CCRN4L |  | 0.4856/0.4374 ** | 0.5461/0.5295 | 0.4007/0.3904 |
| cg02830749 | 4 | 154451068 | KIAA0922 |  | 0.5577/0.4455 ** | 0.6671/0.5972 | 0.0683/0.0538 |
| cg05883128 | 4 | 169239131 | DDX60 |  | 0.2529/0.1368 ** | 0.4618/0.2283 ** | 0.4945/0.1943 ** |
| cg17384323 | 4 | 169242366 |  |  | 0.7326/0.6221 ** | 0.9097/0.881 | 0.9517/0.9419 |
| cg07406498 | 5 | 969899 |  |  | 0.4964/0.5582 ** | 0.7004/0.692 | 0.772/0.7706 |
| cg00675600 | 5 | 1255458 | TERT |  | 0.4567/0.5431 ** | 0.8697/0.8664 | 0.9162/0.9384 |
| cg13390570 | 5 | 1255616 | TERT |  | 0.3712/0.4917 ** | 0.89/0.8884 | 0.9589/0.9583 |
| cg24894783 | 5 | 10577031 | ANKRD33B |  | 0.7187/0.649 ** | 0.8065/0.7463 | 0.5534/0.4734 |
| cg22953759 | 5 | 10632397 | ANKRD33B |  | 0.3134/0.2353 ** | 0.0488/0.0545 | 0.0443/0.044 |
| cg15637765 | 5 | 14145681 | TRIO |  | 0.4095/0.415 | 0.1209/0.1842 ** | 0.3232/0.3449 |
| cg15529432 | 5 | 16615750 | FAM134B |  | 0.5958/0.6897 ** | 0.9449/0.9448 | 0.982/0.9851 |
| cg00461022 | 5 | 16618052 | FAM134B |  | 0.3219/0.4006 ** | 0.4613/0.5028 | 0.783/0.8453 |
| cg07749597 | 5 | 42840698 |  |  | 0.6254/0.5262 ** | 0.6823/0.6482 | 0.1069/0.1026 |
| cg27031754 | 5 | 54185940 |  |  | 0.7147/0.7759 ** | 0.0599/0.0866 | 0.9421/0.9396 |
| cg09452568 | 5 | 54275198 | ESM1 |  | 0.7722/0.6895 ** | 0.9333/0.9062 | 0.3398/0.3046 |
| cg01096487 | 5 | 55184235 | IL31RA |  | 0.4687/0.5709 ** | 0.9161/0.9115 | 0.9504/0.9416 |
| cg20536364 | 5 | 55790102 |  |  | 0.8294/0.7668 ** | 0.8423/0.8188 | 0.6995/0.6423 |
| cg11497377 | 5 | 65465543 | SFRS12 |  | 0.3602/0.444 ** | 0.2716/0.2834 | 0.6971/0.7193 |
| cg10616795 | 5 | 76464212 |  |  | 0.902/0.8523 * | 0.9104/0.8529 ** | 0.8384/0.7628 |
| cg02125365 | 5 | 78813058 |  |  | 0.4141/0.5089 ** | 0.8019/0.79 | 0.8401/0.851 |
| cg06677021 | 5 | 79490511 | SERINC5 |  | 0.2552/0.3403 ** | 0.7089/0.7229 | 0.6035/0.6605 |
| cg16518115 | 5 | 79549315 | SERINC5 |  | 0.3453/0.4329 ** | 0.753/0.7475 | 0.837/0.8211 |
| cg10154812 | 5 | 80253900 |  |  | 0.3989/0.5174 ** | 0.7844/0.8045 | 0.9174/0.913 |
| cg07019857 | 5 | 106822849 | EFNA5 |  | 0.72/0.6484 ** | 0.7785/0.756 | 0.499/0.4768 |
| cg09035699 | 5 | 131310461 | ACSL6 |  | 0.481/0.5885 ** | 0.9354/0.9276 | 0.9747/0.9705 |
| cg27024654 | 5 | 133904674 | PHF15 |  | 0.7285/0.6612 ** | 0.7258/0.6851 | 0.6166/0.5902 |
| cg12499311 | 5 | 134120499 | DDX46 |  | 0.6916/0.7467 ** | 0.791/0.81 | 0.8811/0.8936 |
| cg21069500 | 5 | 139050159 | CXXC5 |  | 0.532/0.4361 ** | 0.0656/0.0745 | 0.2824/0.2716 |
| cg08523384 | 5 | 141488047 | NDFIP1 |  | 0.2217/0.2886 ** | 0.6743/0.6614 | 0.5101/0.5299 |
| cg27579771 | 5 | 142431272 | ARHGAP26 |  | 0.6482/0.5364 ** | 0.6178/0.6025 | 0.0545/0.0511 |
| cg05203346 | 5 | 143278195 |  |  | 0.5582/0.6547 ** | 0.9348/0.9287 | 0.9714/0.9646 |
| cg04605590 | 5 | 148185121 |  |  | 0.1875/0.1439 ** | 0.0623/0.0572 | 0.0241/0.0233 |
| cg11671363 | 5 | 148810177 | MIR145; LOC728264 |  | 0.8542/0.786 ** | 0.9126/0.862 * | 0.5993/0.5279 |
| cg22941668 | 5 | 148810180 | MIR145; LOC728264 |  | 0.8691/0.8157 ** | 0.923/0.8802 * | 0.6896/0.6352 |
| cg15989436 | 5 | 150465875 |  |  | 0.4166/0.3311 ** | 0.4238/0.4479 | 0.157/0.1725 |
| cg18816534 | 5 | 158385443 | EBF1 |  | 0.2451/0.3227 ** | 0.0497/0.0729 * | 0.6548/0.6437 |
| cg08688907 | 5 | 159890925 |  |  | 0.6641/0.5708 ** | 0.9153/0.9 | 0.2969/0.285 |
| cg17969271 | 5 | 171430188 | FBXW11 |  | 0.457/0.3626 ** | 0.5761/0.5207 | 0.1265/0.1095 |
| cg06625767 | 5 | 176836695 | F12 |  | 0.7349/0.667 ** | 0.8313/0.8059 | 0.5117/0.4647 |
| cg13995774 | 5 | 179189810 | MAML1 |  | 0.7299/0.6621 ** | 0.8671/0.8343 | 0.4771/0.4341 |
| cg20661080 | 5 | 180111966 |  |  | 0.3059/0.4197 ** | 0.8471/0.8351 | 0.9034/0.8908 |
| cg25937884 | 5 | 180674133 |  |  | 0.1635/0.1221 ** | 0.0979/0.0896 | 0.0756/0.0731 |
| cg26166854 | 6 | 6614447 | LOC285780; LY86 |  | 0.2359/0.3349 ** | 0.925/0.9047 | 0.9194/0.917 |
| cg07970799 | 6 | 6614719 | LOC285780; LY86 |  | 0.3571/0.4582 ** | 0.9007/0.8907 | 0.9313/0.9331 |
| cg11302401 | 6 | 6688847 |  |  | 0.722/0.654 ** | 0.8442/0.8041 | 0.5455/0.4529 |
| cg09202659 | 6 | 15380565 | JARID2 |  | 0.8527/0.8077 ** | 0.866/0.8419 | 0.7926/0.7553 |
| cg00151370 | 6 | 16323285 | ATXN1 |  | 0.1216/0.1737 ** | 0.2215/0.2135 | 0.2655/0.2989 |
| cg19988367 | 6 | 21808465 | FLJ22536 |  | 0.4903/0.5702 ** | 0.7492/0.743 | 0.723/0.7728 |
| cg00895196 | 6 | 22147182 | FLJ22536 |  | 0.6144/0.6753 ** | 0.9698/0.9621 | 0.9532/0.9551 |
| cg22146593 | 6 | 24919003 |  |  | 0.4764/0.5497 ** | 0.4647/0.4877 | 0.7748/0.7967 |
| cg15019694 | 6 | 26217890 | HIST1H2BG |  | 0.0942/0.0656 ** | 0.0413/0.0373 | 0.033/0.03 |
| cg09296453 | 6 | 29692035 | HLA-F | IFN | 0.1135/0.0935 | 0.2123/0.1359 ** | 0.1664/0.108 |
| cg12588917 | 6 | 29692082 | HLA-F | IFN | 0.3727/0.3472 | 0.5192/0.4449 ** | 0.5117/0.4246 * |
| cg23892836 | 6 | 29692085 | HLA-F | IFN | 0.3968/0.3798 | 0.5674/0.4859 ** | 0.6663/0.5684 * |
| cg24351901 | 6 | 29692092 | HLA-F | IFN | 0.1982/0.18 | 0.3447/0.289 ** | 0.3597/0.2866 * |
| cg15331332 | 6 | 29692111 | HLA-F | IFN | 0.3512/0.3406 | 0.5315/0.4507 * | 0.6839/0.5552 ** |
| cg11617938 | 6 | 29692281 | HLA-F | IFN | 0.607/0.592 | 0.7683/0.7219 | 0.9131/0.8392 ** |
| cg03725115 | 6 | 30458102 | HLA-E | IFN | 0.0935/0.0825 | 0.149/0.111 ** | 0.1235/0.0848 |
| cg15878619 | 6 | 30687373 | TUBB |  | 0.1369/0.1046 ** | 0.0436/0.0413 | 0.0397/0.037 |
| cg23953820 | 6 | 30851051 | DDR1 |  | 0.5133/0.578 * | 0.1524/0.2048 ** | 0.8276/0.8301 |
| cg08469255 | 6 | 30851069 | DDR1 |  | 0.5732/0.648 ** | 0.2985/0.3606 | 0.9055/0.9027 |
| cg16215084 | 6 | 30853948 | DDR1 |  | 0.3221/0.4122 ** | 0.8452/0.8437 | 0.8659/0.8805 |
| cg25251478 | 6 | 30853959 | DDR1 |  | 0.3952/0.4812 ** | 0.867/0.8631 | 0.8816/0.8892 |
| cg26321999 | 6 | 30854011 | DDR1 |  | 0.2162/0.2679 ** | 0.4882/0.4959 | 0.4501/0.492 |
| cg25954539 | 6 | 31323677 | HLA-B | IFN | 0.1325/0.1125 | 0.268/0.1867 ** | 0.319/0.1889 |
| cg17159161 | 6 | 31323760 | HLA-B | IFN | 0.0296/0.0258 | 0.0647/0.0454 ** | 0.0333/0.0251 |
| cg25843003 | 6 | 31431312 | HCP5 |  | 0.0967/0.0785 * | 0.1706/0.1199 ** | 0.2105/0.106 * |
| cg00218406 | 6 | 31431407 | HCP5 |  | 0.2284/0.1932 * | 0.3433/0.2337 ** | 0.508/0.3009 * |
| cg18808777 | 6 | 31431503 | HCP5 |  | 0.2222/0.1935 | 0.389/0.2556 ** | 0.5182/0.3251 * |
| cg09037630 | 6 | 31528239 |  |  | 0.378/0.3035 ** | 0.0312/0.0338 | 0.035/0.0335 |
| cg19279042 | 6 | 31550090 | LTB |  | 0.1272/0.166 ** | 0.0623/0.0768 | 0.352/0.3691 |
| cg14943908 | 6 | 31589196 | BAT2 |  | 0.1166/0.0939 ** | 0.093/0.0813 * | 0.0462/0.0451 |
| cg05554966 | 6 | 31648514 | LY6G5C |  | 0.1957/0.1688 ** | 0.1151/0.1098 | 0.0882/0.0824 |
| cg09993780 | 6 | 31648544 | LY6G5C |  | 0.1813/0.1544 ** | 0.1093/0.1046 | 0.0646/0.0626 |
| cg05032934 | 6 | 31789161 |  |  | 0.1636/0.1272 ** | 0.1464/0.128 | 0.0665/0.0633 |
| cg14635654 | 6 | 32154447 | PBX2 |  | 0.6978/0.7089 | 0.686/0.7187 ** | 0.7572/0.7815 |
| cg22940798 | 6 | 32805554 | TAP2 |  | 0.3409/0.2833 ** | 0.4533/0.3796 * | 0.3877/0.3207 * |
| cg08998192 | 6 | 32805570 | TAP2 |  | 0.3905/0.3323 ** | 0.4475/0.3838 * | 0.446/0.3662 * |
| cg08818207 | 6 | 32820355 | TAP1 |  | 0.222/0.1974 | 0.4135/0.2874 ** | 0.5448/0.359 ** |
| cg24154161 | 6 | 32820421 | TAP1 |  | 0.0233/0.021 | 0.049/0.0364 ** | 0.0449/0.0271 * |
| cg13403689 | 6 | 32820641 | TAP1; PSMB9 |  | 0.0642/0.0592 * | 0.0708/0.0599 ** | 0.0998/0.0768 ** |
| cg02567488 | 6 | 32822565 | TAP1; PSMB9 |  | 0.0321/0.03 | 0.0453/0.0322 ** | 0.0403/0.0289 |
| cg10453850 | 6 | 32905320 | HLA-DMB |  | 0.5578/0.4635 * | 0.3018/0.3931 ** | 0.0279/0.0225 |
| cg21470947 | 6 | 32909493 | HLA-DMB |  | 0.0868/0.1136 ** | 0.0275/0.0338 | 0.1851/0.218 |
| cg07623567 | 6 | 32909523 | HLA-DMB |  | 0.0954/0.1222 ** | 0.0325/0.0388 | 0.2424/0.2634 |
| cg20595453 | 6 | 33219392 | VPS52 |  | 0.5706/0.5275 * | 0.7345/0.6641 ** | 0.6973/0.6116 |
| cg09027493 | 6 | 35109548 | TCP11 |  | 0.6607/0.7301 ** | 0.7994/0.7915 | 0.976/0.9764 |
| cg03546163 | 6 | 35654363 | FKBP5 |  | 0.2869/0.2056 * | 0.6387/0.5218 ** | 0.429/0.2296 * |
| cg20813374 | 6 | 35657180 | FKBP5 |  | 0.4965/0.4441 ** | 0.3888/0.3386 | 0.4997/0.4298 * |
| cg00052684 | 6 | 35694245 | FKBP5 |  | 0.2102/0.1589 ** | 0.8396/0.8002 | 0.0921/0.0842 |
| cg25114611 | 6 | 35696870 | LOC285847; FKBP5 |  | 0.3282/0.2713 ** | 0.2312/0.2156 | 0.1661/0.1392 |
| cg12800266 | 6 | 37225002 | TMEM217; TBC1D22B |  | 0.7287/0.6732 ** | 0.8077/0.7759 | 0.546/0.4841 |
| cg05255811 | 6 | 39192009 | KCNK5 |  | 0.6118/0.5121 ** | 0.7816/0.7608 | 0.078/0.0689 |
| cg09130674 | 6 | 39195019 | KCNK5 |  | 0.4553/0.3812 ** | 0.4573/0.4415 | 0.1022/0.0858 |
| cg05568549 | 6 | 41907198 | CCND3 |  | 0.3539/0.2838 ** | 0.4304/0.4041 | 0.0917/0.091 |
| cg27370104 | 6 | 42417938 | TRERF1 |  | 0.2962/0.3614 ** | 0.682/0.6622 | 0.5783/0.585 |
| cg05945608 | 6 | 42739639 |  |  | 0.1262/0.1642 ** | 0.2809/0.2746 | 0.3709/0.4073 |
| cg06560379 | 6 | 44231305 | NFKBIE |  | 0.0558/0.0454 ** | 0.031/0.0324 | 0.0353/0.0307 |
| cg24700316 | 6 | 90943562 | BACH2 |  | 0.5636/0.6452 ** | 0.8697/0.8556 | 0.918/0.9119 |
| cg05367967 | 6 | 106035490 |  |  | 0.651/0.7264 ** | 0.9423/0.9377 | 0.9569/0.9594 |
| cg02836135 | 6 | 108052093 | SCML4 |  | 0.3833/0.4704 ** | 0.6896/0.6789 | 0.9368/0.9311 |
| cg17117243 | 6 | 109341365 | SESN1 |  | 0.2421/0.3343 ** | 0.5398/0.564 | 0.7189/0.7065 |
| cg16192197 | 6 | 112301106 |  |  | 0.3343/0.4191 ** | 0.8912/0.8873 | 0.7236/0.7754 |
| cg21775668 | 6 | 131147216 | LOC285733 |  | 0.4737/0.5633 ** | 0.7982/0.7948 | 0.943/0.9324 |
| cg22855968 | 6 | 134673162 |  |  | 0.3037/0.396 ** | 0.5486/0.5772 | 0.8025/0.8171 |
| cg07474842 | 6 | 136915088 | MAP3K5 |  | 0.2696/0.2056 ** | 0.0656/0.0727 | 0.0294/0.0247 |
| cg15804973 | 6 | 137114513 | MAP3K5 |  | 0.4945/0.4268 ** | 0.3576/0.333 | 0.4259/0.3859 |
| cg23280720 | 6 | 139483193 | HECA |  | 0.1766/0.2491 ** | 0.2078/0.2249 | 0.695/0.7497 |
| cg16727231 | 6 | 139485336 | HECA |  | 0.353/0.439 ** | 0.8432/0.8345 | 0.8841/0.8712 |
| cg09853238 | 6 | 149532290 |  |  | 0.4104/0.3298 ** | 0.0459/0.0514 | 0.0416/0.0404 |
| cg12603453 | 6 | 151694679 | ZBTB2 |  | 0.2985/0.4217 ** | 0.83/0.7628 | 0.7352/0.7282 |
| cg07212702 | 6 | 157137791 | ARID1B |  | 0.6362/0.5565 ** | 0.627/0.594 | 0.4614/0.3785 |
| cg06012428 | 6 | 157477204 | ARID1B |  | 0.8452/0.7905 ** | 0.9381/0.9221 | 0.6648/0.6396 |
| cg08109681 | 6 | 166825084 | RPS6KA2 |  | 0.3544/0.4712 ** | 0.8674/0.8669 | 0.9626/0.9646 |
| cg17744997 | 6 | 167401611 |  |  | 0.3647/0.4568 ** | 0.8082/0.8041 | 0.6814/0.682 |
| cg02217713 | 7 | 643155 | PRKAR1B |  | 0.6663/0.5652 ** | 0.7786/0.709 * | 0.2891/0.2539 |
| cg22662844 | 7 | 2116648 | MAD1L1 |  | 0.7313/0.6482 ** | 0.9135/0.87 * | 0.4592/0.3902 |
| cg12030710 | 7 | 2444010 | CHST12 |  | 0.112/0.0778 ** | 0.0433/0.0396 | 0.0313/0.0272 |
| cg10152449 | 7 | 2444534 | CHST12 |  | 0.5049/0.3925 ** | 0.3286/0.2143 ** | 0.0753/0.0487 |
| cg21159568 | 7 | 2445331 | CHST12 |  | 0.4384/0.3667 * | 0.4717/0.3336 ** | 0.09/0.0827 |
| cg22788953 | 7 | 2679148 | TTYH3 |  | 0.8933/0.8962 | 0.8566/0.778 ** | 0.8255/0.8634 |
| cg24375364 | 7 | 2755000 | AMZ1 |  | 0.4579/0.5433 ** | 0.2462/0.2853 | 0.7924/0.8115 |
| cg13287553 | 7 | 4784419 | FOXK1 |  | 0.0945/0.1318 ** | 0.1444/0.1493 | 0.2339/0.2293 |
| cg07281318 | 7 | 6575968 | GRID2IP |  | 0.2968/0.25 ** | 0.0632/0.0669 | 0.0965/0.1051 |
| cg21699330 | 7 | 26193032 | NFE2L3 |  | 0.2665/0.1746 ** | 0.128/0.089 * | 0.0578/0.0461 |
| cg10536999 | 7 | 26193109 | NFE2L3 |  | 0.1104/0.0742 ** | 0.0465/0.0371 | 0.023/0.0196 |
| cg08822075 | 7 | 26193607 | NFE2L3 |  | 0.4487/0.3547 ** | 0.4441/0.3682 * | 0.3099/0.2614 |
| cg12510708 | 7 | 26193805 | NFE2L3 |  | 0.4745/0.3508 ** | 0.4511/0.403 | 0.0394/0.0362 |
| cg02010481 | 7 | 28218524 | JAZF1 |  | 0.3199/0.2509 ** | 0.4809/0.4442 | 0.0683/0.0611 |
| cg14772935 | 7 | 29187019 | CPVL |  | 0.483/0.3996 ** | 0.6428/0.6129 | 0.1778/0.1834 |
| cg11251470 | 7 | 30008931 | SCRN1 |  | 0.5493/0.6403 ** | 0.9484/0.9421 | 0.9765/0.978 |
| cg04858110 | 7 | 30009236 | SCRN1 |  | 0.4841/0.5646 ** | 0.9081/0.9011 | 0.9486/0.9461 |
| cg04065210 | 7 | 35074628 | DPY19L1 |  | 0.1506/0.1948 ** | 0.2954/0.3136 | 0.2881/0.316 |
| cg13800005 | 7 | 39629290 |  |  | 0.3547/0.2862 ** | 0.4834/0.4831 | 0.1061/0.1038 |
| cg26478599 | 7 | 41747322 | LOC285954 |  | 0.7722/0.6914 ** | 0.9156/0.8903 | 0.4986/0.4519 |
| cg03490567 | 7 | 43944817 | URGCP |  | 0.4688/0.5413 ** | 0.4571/0.5152 | 0.6289/0.6596 |
| cg18442362 | 7 | 44677772 | OGDH |  | 0.3066/0.2324 ** | 0.5798/0.5185 | 0.0444/0.0415 |
| cg13400493 | 7 | 44826786 |  |  | 0.7232/0.7203 | 0.1249/0.1885 ** | 0.9347/0.9284 |
| cg07826859 | 7 | 45020086 | MYO1G |  | 0.3807/0.3053 ** | 0.3939/0.3572 | 0.1481/0.1634 |
| cg12573289 | 7 | 45075791 | CCM2 |  | 0.2313/0.2955 ** | 0.5277/0.5412 | 0.5496/0.5907 |
| cg26720010 | 7 | 45147131 | SNORA5B; TBRG4 |  | 0.412/0.5183 ** | 0.9186/0.9114 | 0.9448/0.9379 |
| cg11494773 | 7 | 48128242 | UPP1 |  | 0.1288/0.099 ** | 0.0349/0.0344 | 0.0198/0.0184 |
| cg25404758 | 7 | 70139561 | AUTS2 |  | 0.523/0.6331 ** | 0.9338/0.9301 | 0.9201/0.9212 |
| cg00277397 | 7 | 71800412 | CALN1 |  | 0.8724/0.8114 ** | 0.8694/0.8255 * | 0.83/0.7859 |
| cg24300607 | 7 | 73699346 |  |  | 0.6326/0.5477 ** | 0.4325/0.4087 | 0.2397/0.2433 |
| cg06825878 | 7 | 75472540 |  |  | 0.3108/0.2328 ** | 0.614/0.5615 | 0.0529/0.0481 |
| cg00907204 | 7 | 92461971 | CDK6 |  | 0.0869/0.0675 ** | 0.0473/0.044 | 0.0371/0.0342 |
| cg21810604 | 7 | 99699701 | MCM7; AP4M1 |  | 0.0643/0.0512 ** | 0.0348/0.0337 | 0.0305/0.0285 |
| cg24616382 | 7 | 99767035 | GAL3ST4 |  | 0.4246/0.5228 ** | 0.6001/0.6093 | 0.9008/0.8923 |
| cg09507934 | 7 | 102072549 | ORAI2 |  | 0.154/0.2191 ** | 0.2351/0.249 | 0.4415/0.4818 |
| cg25652701 | 7 | 105348517 | ATXN7L1 |  | 0.0611/0.0954 ** | 0.2469/0.2471 | 0.0538/0.0533 |
| cg27284331 | 7 | 106297689 |  |  | 0.6418/0.5661 * | 0.276/0.1922 ** | 0.5141/0.4354 |
| cg06545367 | 7 | 110731527 | LRRN3; IMMP2L |  | 0.5296/0.6249 ** | 0.8978/0.8803 | 0.8874/0.8952 |
| cg24404329 | 7 | 115849899 | TES |  | 0.5715/0.6494 ** | 0.92/0.9236 | 0.8971/0.9011 |
| cg12669355 | 7 | 116514552 | CAPZA2 |  | 0.3284/0.4238 ** | 0.8177/0.8237 | 0.8464/0.8636 |
| cg16983084 | 7 | 127117859 |  |  | 0.4598/0.546 ** | 0.7931/0.793 | 0.8701/0.8683 |
| cg13914531 | 7 | 128579876 | IRF5 | IFN | 0.2876/0.2353 ** | 0.088/0.0878 | 0.0967/0.0965 |
| cg05904013 | 7 | 128579933 | IRF5 | IFN | 0.5691/0.4931 ** | 0.3803/0.3335 | 0.3842/0.3594 |
| cg04864179 | 7 | 128579964 | IRF5 | IFN | 0.6984/0.6275 ** | 0.4755/0.4335 | 0.5281/0.5092 |
| cg11961845 | 7 | 129008179 | AHCYL2 |  | 0.3419/0.2849 ** | 0.3341/0.3221 | 0.0917/0.1072 |
| cg22544881 | 7 | 130712346 | FLJ43663 |  | 0.4147/0.3234 ** | 0.4858/0.4564 | 0.0433/0.04 |
| cg03873220 | 7 | 139760375 | PARP12 |  | 0.712/0.6536 ** | 0.8201/0.763 | 0.9358/0.9311 |
| cg12013713 | 7 | 139760671 | PARP12 |  | 0.6877/0.5967 ** | 0.7709/0.7079 ** | 0.5938/0.5049 |
| cg05994974 | 7 | 139761087 | PARP12 |  | 0.5036/0.3371 ** | 0.4731/0.3463 ** | 0.2719/0.1315 ** |
| cg20039211 | 7 | 142548670 |  |  | 0.586/0.6545 ** | 0.6912/0.686 | 0.8327/0.8127 |
| cg01364581 | 7 | 150175800 | GIMAP8 |  | 0.5215/0.5934 ** | 0.7446/0.7253 | 0.8318/0.851 |
| cg16532400 | 7 | 150217056 | GIMAP7 |  | 0.3472/0.4612 ** | 0.9194/0.9082 | 0.9858/0.9896 |
| cg10777178 | 7 | 150264284 | GIMAP4 |  | 0.2817/0.3287 ** | 0.551/0.5437 | 0.5167/0.52 |
| cg13662290 | 7 | 150264311 | GIMAP4 |  | 0.1984/0.2521 ** | 0.5118/0.5226 | 0.4684/0.4988 |
| cg00323915 | 7 | 150264987 | GIMAP4 |  | 0.1942/0.2688 ** | 0.6589/0.6527 | 0.5826/0.637 |
| cg16908215 | 7 | 150440016 | GIMAP5 |  | 0.4384/0.5361 ** | 0.9204/0.9086 | 0.9733/0.9666 |
| cg13104880 | 7 | 155790512 |  |  | 0.3117/0.3846 ** | 0.793/0.79 | 0.8668/0.8659 |
| cg02704570 | 7 | 157647109 | PTPRN2 |  | 0.357/0.2891 ** | 0.1018/0.1024 | 0.0595/0.0601 |
| cg01462349 | 7 | 157664568 | PTPRN2 |  | 0.3259/0.4082 ** | 0.7265/0.7297 | 0.7833/0.7789 |
| cg14383815 | 8 | 6282955 | MCPH1 |  | 0.4492/0.4611 | 0.2209/0.2792 ** | 0.443/0.4699 |
| cg17090611 | 8 | 17017866 | ZDHHC2 |  | 0.7815/0.6981 ** | 0.9172/0.8907 | 0.3445/0.3003 |
| cg03651021 | 8 | 19317380 | CSGALNACT1 |  | 0.5221/0.6215 ** | 0.8183/0.8093 | 0.9582/0.949 |
| cg25764534 | 8 | 22485721 | BIN3 |  | 0.7408/0.6893 ** | 0.6945/0.6722 | 0.6404/0.6272 |
| cg09177577 | 8 | 22503577 | BIN3 |  | 0.6035/0.6976 ** | 0.9209/0.9161 | 0.9075/0.9063 |
| cg13580286 | 8 | 22925391 | TNFRSF10B |  | 0.1662/0.1351 ** | 0.0585/0.0592 | 0.0428/0.0382 |
| cg22022716 | 8 | 26276541 |  |  | 0.8964/0.8615 ** | 0.9024/0.888 | 0.8436/0.8026 |
| cg00420997 | 8 | 29607076 | C8orf75 |  | 0.1993/0.1551 ** | 0.0622/0.0692 | 0.0887/0.0849 |
| cg12931554 | 8 | 37119428 |  |  | 0.0862/0.1278 ** | 0.1593/0.1589 | 0.066/0.0668 |
| cg10705306 | 8 | 37337009 |  |  | 0.2819/0.2086 ** | 0.1625/0.1352 | 0.0439/0.0414 |
| cg27128883 | 8 | 37438843 |  |  | 0.717/0.7917 ** | 0.9441/0.9564 | 0.9775/0.9784 |
| cg08573701 | 8 | 53603035 | RB1CC1 |  | 0.451/0.5525 ** | 0.9626/0.9597 | 0.9811/0.9788 |
| cg15973818 | 8 | 53623995 | RB1CC1 |  | 0.5691/0.6562 ** | 0.9046/0.8922 | 0.9399/0.9363 |
| cg26720452 | 8 | 57472776 |  |  | 0.1797/0.1508 ** | 0.1794/0.1674 | 0.0552/0.0533 |
| cg14719959 | 8 | 61777711 | CHD7 |  | 0.4974/0.553 ** | 0.8732/0.864 | 0.8765/0.8696 |
| cg25011252 | 8 | 61777859 | CHD7 |  | 0.4056/0.4738 ** | 0.8596/0.8521 | 0.9295/0.9204 |
| cg14864167 | 8 | 66751182 | PDE7A |  | 0.1869/0.1561 | 0.4903/0.287 ** | 0.5439/0.1758 ** |
| cg21750887 | 8 | 68658237 | CPA6 |  | 0.4091/0.4674 ** | 0.6676/0.678 | 0.6568/0.6905 |
| cg26228558 | 8 | 71527496 |  |  | 0.1887/0.2394 ** | 0.8037/0.7508 | 0.4454/0.5124 |
| cg19925215 | 8 | 80964918 | TPD52 |  | 0.4473/0.5191 ** | 0.698/0.7134 | 0.7296/0.7438 |
| cg15723028 | 8 | 90776474 | RIPK2 |  | 0.5437/0.4242 ** | 0.6455/0.5854 | 0.0818/0.0896 |
| cg00219816 | 8 | 96280555 | C8orf37 |  | 0.7674/0.7071 ** | 0.8471/0.8249 | 0.4466/0.4259 |
| cg13675051 | 8 | 101315498 | RNF19A |  | 0.4515/0.3523 ** | 0.8901/0.8598 | 0.0746/0.0738 |
| cg01596292 | 8 | 101315505 | RNF19A |  | 0.4957/0.4171 ** | 0.8771/0.8533 | 0.3456/0.3128 |
| cg07594831 | 8 | 101315560 | RNF19A |  | 0.5915/0.4925 ** | 0.9478/0.9164 * | 0.236/0.2278 |
| cg16683073 | 8 | 117499390 |  |  | 0.5162/0.5987 ** | 0.8761/0.8661 | 0.932/0.917 |
| cg05127574 | 8 | 121714454 | SNTB1 |  | 0.5364/0.6467 ** | 0.5111/0.5255 | 0.9463/0.9437 |
| cg05753328 | 8 | 124171421 |  |  | 0.6379/0.5587 ** | 0.4532/0.4213 | 0.436/0.3996 |
| cg12002745 | 8 | 124179875 |  |  | 0.4514/0.3781 ** | 0.698/0.6656 | 0.0491/0.0493 |
| cg12616923 | 8 | 128228107 |  |  | 0.4222/0.3265 ** | 0.1146/0.1286 | 0.0566/0.0555 |
| cg16675872 | 8 | 130253618 |  |  | 0.3977/0.5023 ** | 0.9209/0.9232 | 0.983/0.9794 |
| cg11909467 | 8 | 132912348 |  |  | 0.4493/0.5501 ** | 0.8935/0.892 | 0.9492/0.9509 |
| cg10054641 | 8 | 133773093 | TMEM71 |  | 0.1967/0.25 ** | 0.7661/0.7294 | 0.4199/0.4452 |
| cg23313885 | 8 | 133773115 | TMEM71 |  | 0.2891/0.3596 ** | 0.6815/0.6549 | 0.5048/0.509 |
| cg17090901 | 8 | 133837475 | PHF20L1 |  | 0.5728/0.6555 ** | 0.8578/0.8407 | 0.8392/0.8513 |
| cg20229014 | 8 | 134529378 | ST3GAL1 |  | 0.0431/0.0567 ** | 0.0931/0.0879 | 0.0491/0.0488 |
| cg05037806 | 8 | 143407432 | TSNARE1 |  | 0.77/0.7163 ** | 0.8009/0.8111 | 0.6526/0.5783 |
| cg06517984 | 8 | 143407646 | TSNARE1 |  | 0.7953/0.7259 ** | 0.7909/0.8157 | 0.5783/0.5072 |
| cg02230964 | 8 | 143407817 | TSNARE1 |  | 0.7372/0.6701 ** | 0.7306/0.7509 | 0.4804/0.4129 |
| cg25875163 | 8 | 143763340 | PSCA |  | 0.8097/0.7664 ** | 0.8802/0.871 | 0.802/0.769 |
| cg06894628 | 8 | 143822543 | SLURP1 |  | 0.5168/0.566 ** | 0.516/0.539 | 0.6651/0.6845 |
| cg12110437 | 8 | 144098888 | LY6E; LOC100133669 |  | 0.3084/0.186 ** | 0.0699/0.0722 | 0.3642/0.1451 * |
| cg17052170 | 8 | 144099482 | LY6E; LOC100133669 |  | 0.6545/0.5483 ** | 0.6851/0.5476 ** | 0.5938/0.4439 |
| cg04678793 | 8 | 144099566 | LY6E; LOC100133669 |  | 0.0526/0.0411 ** | 0.0588/0.0429 ** | 0.042/0.0297 |
| cg11702942 | 8 | 144102584 | LY6E |  | 0.7791/0.6624 ** | 0.7861/0.6968 ** | 0.8008/0.6748 * |
| cg14392283 | 8 | 144103587 | LY6E |  | 0.8977/0.7314 ** | 0.842/0.6474 ** | 0.9613/0.9257 |
| cg03587597 | 8 | 144105055 |  |  | 0.8599/0.8024 ** | 0.8803/0.8442 * | 0.8786/0.8117 * |
| cg16400320 | 8 | 144105210 |  |  | 0.8222/0.7379 ** | 0.9118/0.8589 ** | 0.8484/0.7594 * |
| cg12906975 | 8 | 144105259 |  |  | 0.9266/0.844 ** | 0.9173/0.8416 ** | 0.9345/0.8534 * |
| cg07971089 | 8 | 144629702 |  |  | 0.2572/0.1784 ** | 0.2979/0.1983 * | 0.0255/0.0218 |
| cg16001422 | 8 | 145022842 | PLEC1 |  | 0.1547/0.1216 ** | 0.4257/0.3879 | 0.0693/0.0646 |
| cg21927363 | 9 | 15552206 | C9orf93 |  | 0.1058/0.0847 ** | 0.0603/0.0569 | 0.0427/0.0405 |
| cg03848588 | 9 | 32525008 | DDX58 | IFN | 0.8901/0.8235 ** | 0.9157/0.878 * | 0.9018/0.8446 * |
| cg13752545 | 9 | 38071235 |  |  | 0.5049/0.585 ** | 0.7389/0.7422 | 0.7847/0.7745 |
| cg21171339 | 9 | 79791169 | VPS13A |  | 0.4285/0.5013 ** | 0.5665/0.583 | 0.674/0.6856 |
| cg13918640 | 9 | 94351568 |  |  | 0.6598/0.5887 ** | 0.8058/0.7454 | 0.4433/0.4106 |
| cg11516606 | 9 | 100175029 | TDRD7 |  | 0.0655/0.0487 ** | 0.0434/0.0402 | 0.0205/0.0191 |
| cg11317199 | 9 | 100850391 | TRIM14 |  | 0.5968/0.6858 ** | 0.421/0.5732 ** | 0.588/0.7958 * |
| cg01765174 | 9 | 100880960 | TRIM14 |  | 0.4514/0.3903 ** | 0.6586/0.5836 | 0.5031/0.4179 * |
| cg13827209 | 9 | 101912842 | TGFBR1 |  | 0.0857/0.1321 ** | 0.2103/0.2495 * | 0.133/0.1733 |
| cg13633625 | 9 | 114706971 |  |  | 0.8456/0.7808 ** | 0.9058/0.8727 | 0.685/0.6328 |
| cg15551881 | 9 | 123688715 | TRAF1 |  | 0.3358/0.4358 ** | 0.5372/0.4987 | 0.7129/0.7577 |
| cg13696706 | 9 | 124396830 | DAB2IP |  | 0.2382/0.2765 ** | 0.1231/0.1354 | 0.4492/0.4706 |
| cg13473120 | 9 | 126776767 | LHX2 |  | 0.0265/0.0356 ** | 0.0399/0.041 | 0.033/0.0328 |
| cg13753351 | 9 | 127134207 | PSMB7 |  | 0.6685/0.5799 ** | 0.879/0.8563 | 0.418/0.3364 |
| cg14364797 | 9 | 132651576 | FNBP1 |  | 0.3484/0.4426 ** | 0.5229/0.5 | 0.8105/0.8052 |
| cg10531986 | 9 | 132652466 | FNBP1 |  | 0.5186/0.6055 ** | 0.8915/0.8819 | 0.9172/0.9134 |
| cg06901890 | 9 | 132803508 | FNBP1 |  | 0.0768/0.0979 ** | 0.1017/0.1073 | 0.1775/0.2046 |
| cg14289429 | 9 | 134139878 | FAM78A |  | 0.4129/0.4868 ** | 0.8798/0.8638 | 0.4946/0.5438 |
| cg14283140 | 9 | 135763667 | C9orf9 |  | 0.8817/0.9002 | 0.0896/0.1258 ** | 0.9435/0.9455 |
| cg13692739 | 9 | 139005232 |  |  | 0.1622/0.2072 ** | 0.4727/0.4777 | 0.403/0.4513 |
| cg15986644 | 10 | 516683 | DIP2C |  | 0.5877/0.663 ** | 0.9703/0.9611 | 0.9744/0.9742 |
| cg17115419 | 10 | 5593926 |  |  | 0.3166/0.3807 ** | 0.7699/0.7483 | 0.6357/0.6855 |
| cg09234252 | 10 | 8373659 |  |  | 0.358/0.481 ** | 0.884/0.8802 | 0.9084/0.9186 |
| cg03412153 | 10 | 13139217 |  |  | 0.3051/0.3934 ** | 0.7172/0.7217 | 0.8768/0.8738 |
| cg08791347 | 10 | 13831250 | FRMD4A |  | 0.2492/0.3494 ** | 0.5924/0.6062 | 0.449/0.4656 |
| cg15609734 | 10 | 17689470 | STAM |  | 0.5377/0.4249 ** | 0.212/0.2072 | 0.0646/0.0577 |
| cg14014799 | 10 | 22606053 | COMMD3 |  | 0.1719/0.117 ** | 0.0702/0.0734 | 0.0791/0.0739 |
| cg19378631 | 10 | 22606072 | COMMD3 |  | 0.1854/0.1243 ** | 0.0315/0.0336 | 0.0281/0.0236 |
| cg04691264 | 10 | 29697905 | LOC387647 |  | 0.6077/0.7176 ** | 0.8812/0.8816 | 0.9429/0.9369 |
| cg00572560 | 10 | 30093517 |  |  | 0.2279/0.273 ** | 0.4592/0.4712 | 0.4295/0.4409 |
| cg23648810 | 10 | 30337992 | KIAA1462 |  | 0.0975/0.1252 ** | 0.1202/0.1245 | 0.2064/0.2421 |
| cg12753529 | 10 | 51525504 |  |  | 0.518/0.5852 ** | 0.9329/0.9258 | 0.843/0.8577 |
| cg18522931 | 10 | 63776828 | ARID5B |  | 0.4707/0.3316 ** | 0.061/0.0716 | 0.0724/0.0628 |
| cg24417845 | 10 | 70816008 |  |  | 0.404/0.3358 ** | 0.5927/0.5616 | 0.0504/0.0537 |
| cg04858631 | 10 | 74035570 | DDIT4 |  | 0.2262/0.3056 ** | 0.1448/0.147 | 0.7115/0.7067 |
| cg08945443 | 10 | 75193254 | ZMYND17 |  | 0.3915/0.4998 ** | 0.821/0.8447 | 0.8782/0.8985 |
| cg21398111 | 10 | 75528810 | SEC24C |  | 0.5341/0.6365 ** | 0.6239/0.6234 | 0.9436/0.9429 |
| cg05021029 | 10 | 76947956 |  |  | 0.4904/0.5907 ** | 0.7617/0.752 | 0.9593/0.957 |
| cg08185255 | 10 | 80063568 |  |  | 0.5526/0.6102 ** | 0.5342/0.5492 | 0.8688/0.8613 |
| cg11940526 | 10 | 80311367 |  |  | 0.4962/0.5796 ** | 0.1244/0.1416 | 0.9638/0.9626 |
| cg25526001 | 10 | 85939451 | C10orf99 |  | 0.3913/0.4783 ** | 0.2299/0.2484 | 0.8422/0.8272 |
| cg16341836 | 10 | 90641389 | STAMBPL1 |  | 0.4572/0.3795 ** | 0.4307/0.3772 | 0.4554/0.418 |
| cg23264429 | 10 | 90642003 | STAMBPL1 |  | 0.5293/0.3954 ** | 0.5363/0.4791 | 0.5372/0.4474 |
| cg14785527 | 10 | 90656978 | STAMBPL1 |  | 0.2858/0.2285 ** | 0.3402/0.3446 | 0.0728/0.0835 |
| cg15774510 | 10 | 90749966 | ACTA2; FAS |  | 0.2085/0.1505 ** | 0.0465/0.0421 | 0.0236/0.0227 |
| cg16257983 | 10 | 90750218 | ACTA2; FAS |  | 0.0721/0.0594 ** | 0.043/0.0431 | 0.0496/0.0453 |
| cg02314339 | 10 | 91020653 |  |  | 0.7778/0.715 ** | 0.8537/0.8165 * | 0.6387/0.5667 |
| cg27478224 | 10 | 91061115 | IFIT2 | IFN | 0.0786/0.0568 ** | 0.0486/0.0454 | 0.0528/0.0484 |
| cg06188083 | 10 | 91093005 | IFIT3 | IFN | 0.3073/0.1714 ** | 0.2701/0.1289 ** | 0.4207/0.1225 ** |
| cg02370832 | 10 | 91093681 | IFIT3 | IFN | 0.383/0.2793 ** | 0.4521/0.2753 ** | 0.5359/0.2824 * |
| cg26974214 | 10 | 91151885 | IFIT1 | IFN | 0.1266/0.0867 ** | 0.1121/0.082 ** | 0.0716/0.058 |
| cg05552874 | 10 | 91153143 | IFIT1 | IFN | 0.6583/0.3874 ** | 0.6731/0.3878 ** | 0.4025/0.1165 ** |
| cg06376949 | 10 | 91173811 | IFIT5 | IFN | 0.4149/0.2749 ** | 0.3683/0.2812 ** | 0.2159/0.138 * |
| cg13172359 | 10 | 91175366 | IFIT5 | IFN | 0.3495/0.1842 ** | 0.1258/0.1151 | 0.2072/0.1331 |
| cg08284263 | 10 | 92958627 |  |  | 0.3367/0.2555 ** | 0.0847/0.0932 | 0.0589/0.0502 |
| cg01391548 | 10 | 97068696 |  |  | 0.5276/0.4806 | 0.6088/0.5291 ** | 0.3575/0.3116 |
| cg03190891 | 10 | 97201172 | SORBS1 |  | 0.2033/0.2431 ** | 0.2285/0.2447 | 0.4198/0.4529 |
| cg13471990 | 10 | 97515222 | ENTPD1 |  | 0.4828/0.3869 ** | 0.4542/0.447 | 0.03/0.0281 |
| cg10381771 | 10 | 97515398 | ENTPD1 |  | 0.5633/0.4595 ** | 0.6463/0.6386 | 0.0451/0.0426 |
| cg15428620 | 10 | 102792835 | SFXN3 |  | 0.3253/0.2765 ** | 0.5033/0.4784 | 0.2496/0.2418 |
| cg26605164 | 10 | 102821565 | KAZALD1 |  | 0.5778/0.4883 ** | 0.2529/0.2242 | 0.0806/0.0704 |
| cg23691894 | 10 | 111765904 | ADD3 |  | 0.6534/0.7437 ** | 0.9662/0.953 | 0.9618/0.9725 |
| cg03290131 | 10 | 112263831 | DUSP5 |  | 0.2869/0.188 ** | 0.0523/0.0463 | 0.0242/0.0224 |
| cg12649038 | 10 | 116282534 | ABLIM1 |  | 0.4042/0.5067 ** | 0.6528/0.6988 | 0.7208/0.7631 |
| cg23786209 | 10 | 116285554 | ABLIM1 |  | 0.1629/0.2232 ** | 0.1452/0.1856 | 0.4192/0.4324 |
| cg01185921 | 10 | 116444463 | ABLIM1 |  | 0.681/0.6249 ** | 0.6777/0.6316 * | 0.5902/0.5414 |
| cg00291478 | 10 | 121301041 | RGS10 |  | 0.2597/0.3314 ** | 0.6626/0.6702 | 0.6344/0.6618 |
| cg05617307 | 10 | 121413182 | BAG3 |  | 0.5012/0.6217 ** | 0.9179/0.8899 | 0.9163/0.914 |
| cg10462187 | 10 | 124067402 | BTBD16 |  | 0.6439/0.6461 | 0.0714/0.1162 ** | 0.779/0.7946 |
| cg19863426 | 10 | 124138853 | PLEKHA1 |  | 0.4352/0.5405 ** | 0.8469/0.8707 | 0.9987/0.9896 |
| cg02556345 | 10 | 124181965 | PLEKHA1 |  | 0.2471/0.3201 ** | 0.1171/0.1777 * | 0.569/0.5767 |
| cg07858728 | 10 | 124319791 | DMBT1 |  | 0.1895/0.1304 ** | 0.058/0.0598 | 0.0611/0.0585 |
| cg04370247 | 10 | 126308552 | FAM53B |  | 0.5367/0.4599 ** | 0.0453/0.0593 | 0.3232/0.3792 |
| cg24916358 | 10 | 126315761 | FAM53B |  | 0.0894/0.131 ** | 0.0416/0.0495 | 0.3135/0.3782 |
| cg26517376 | 10 | 126428818 | FAM53B |  | 0.3029/0.3745 ** | 0.612/0.6122 | 0.7273/0.724 |
| cg04323925 | 10 | 133742464 |  |  | 0.3698/0.4265 ** | 0.1814/0.2381 | 0.6131/0.6676 |
| cg05432003 | 11 | 312518 | IFITM1 | IFN | 0.3569/0.2951 * | 0.7462/0.5894 ** | 0.7409/0.6467 |
| cg01886988 | 11 | 312560 | IFITM1 | IFN | 0.1474/0.126 * | 0.3957/0.2749 ** | 0.4019/0.3041 * |
| cg27032101 | 11 | 312841 | IFITM1 | IFN | 0.5242/0.4748 | 0.6928/0.5611 ** | 0.8045/0.7414 |
| cg04582010 | 11 | 313120 | IFITM1 | IFN | 0.2297/0.177 * | 0.6119/0.401 ** | 0.6339/0.4715 ** |
| cg09026253 | 11 | 313267 | IFITM1 | IFN | 0.3463/0.3069 | 0.7139/0.4917 ** | 0.8609/0.7286 * |
| cg11694510 | 11 | 313354 | IFITM1 | IFN | 0.4129/0.3809 * | 0.6241/0.4754 ** | 0.8033/0.6828 * |
| cg10552523 | 11 | 313478 | IFITM1 | IFN | 0.1729/0.1287 ** | 0.0605/0.0453 * | 0.525/0.3445 ** |
| cg20566897 | 11 | 313527 | IFITM1 | IFN | 0.2595/0.2081 * | 0.1497/0.0937 ** | 0.6031/0.439 ** |
| cg01971407 | 11 | 313624 | IFITM1 | IFN | 0.2923/0.2385 ** | 0.5257/0.3612 ** | 0.556/0.3857 ** |
| cg23570810 | 11 | 315102 | IFITM1 | IFN | 0.2711/0.1905 ** | 0.746/0.427 ** | 0.8223/0.4353 ** |
| cg21686213 | 11 | 315118 | IFITM1 | IFN | 0.2883/0.275 | 0.8208/0.5372 ** | 0.8475/0.6962 * |
| cg03038262 | 11 | 315262 | IFITM1 | IFN | 0.1896/0.1427 * | 0.6092/0.3158 ** | 0.7699/0.4012 ** |
| cg12047941 | 11 | 315908 |  |  | 0.5185/0.477 | 0.9047/0.7976 ** | 0.7994/0.6996 |
| cg20045320 | 11 | 319555 |  |  | 0.4163/0.3185 * | 0.6541/0.514 ** | 0.563/0.3327 * |
| cg17990365 | 11 | 319718 | IFITM3 | IFN | 0.29/0.2396 * | 0.7326/0.5721 ** | 0.7003/0.4728 ** |
| cg19974879 | 11 | 611692 | PHRF1 |  | 0.9344/0.912 ** | 0.9245/0.8959 * | 0.9354/0.9204 |
| cg11791770 | 11 | 611791 | PHRF1 |  | 0.8763/0.8402 ** | 0.8954/0.8597 * | 0.871/0.8292 |
| cg05309505 | 11 | 612837 | IRF7 | IFN | 0.9657/0.9514 ** | 0.9714/0.9522 * | 0.9555/0.9343 * |
| cg08926253 | 11 | 614761 | IRF7 | IFN | 0.5807/0.4193 ** | 0.6811/0.5414 ** | 0.3958/0.1816 ** |
| cg22016995 | 11 | 614787 | IRF7 | IFN | 0.9371/0.8352 ** | 0.9321/0.8753 * | 0.8823/0.7616 |
| cg11524400 | 11 | 1778524 | CTSD; HCCA2 |  | 0.3384/0.4192 ** | 0.856/0.8632 | 0.9159/0.9071 |
| cg12461141 | 11 | 5710654 | TRIM22 |  | 0.4515/0.3471 ** | 0.4143/0.3583 * | 0.2706/0.2007 |
| cg26724018 | 11 | 5716255 | TRIM22 |  | 0.2138/0.1543 ** | 0.4303/0.3187 ** | 0.2747/0.198 * |
| cg08726522 | 11 | 8739587 | ST5 |  | 0.5151/0.4409 ** | 0.6209/0.6071 | 0.1728/0.1619 |
| cg26707052 | 11 | 13250995 |  |  | 0.8347/0.8453 | 0.1512/0.199 ** | 0.8738/0.8679 |
| cg15846482 | 11 | 18610557 | UEVLD |  | 0.326/0.2551 ** | 0.1057/0.1023 | 0.0525/0.0525 |
| cg23371436 | 11 | 20111534 | NAV2 |  | 0.3796/0.4498 ** | 0.1744/0.21 | 0.6304/0.6499 |
| cg23845009 | 11 | 34323678 | ABTB2 |  | 0.7047/0.6001 ** | 0.897/0.8643 * | 0.3819/0.3323 |
| cg21108085 | 11 | 44591098 | CD82 |  | 0.2897/0.2291 ** | 0.071/0.0689 | 0.0316/0.032 |
| cg25514148 | 11 | 46294375 |  |  | 0.4802/0.5153 ** | 0.699/0.6918 | 0.6737/0.6812 |
| cg12389611 | 11 | 46351906 |  |  | 0.4257/0.5048 ** | 0.8628/0.8506 | 0.6185/0.6245 |
| cg18908017 | 11 | 46353622 | DGKZ |  | 0.6017/0.656 ** | 0.7854/0.7774 | 0.7752/0.7721 |
| cg12027899 | 11 | 58385947 | ZFP91; ZFP91-CNTF |  | 0.6563/0.7106 ** | 0.8472/0.8503 | 0.9268/0.9355 |
| cg00271311 | 11 | 58389290 | CNTF; ZFP91-CNTF |  | 0.2979/0.3795 ** | 0.6665/0.6058 * | 0.7243/0.7163 |
| cg14328641 | 11 | 59822727 | MS4A3 |  | 0.5628/0.4903 ** | 0.691/0.6559 | 0.2922/0.2828 |
| cg03055440 | 11 | 59950405 | MS4A6A |  | 0.7789/0.6904 ** | 0.9201/0.9024 | 0.4504/0.4375 |
| cg05778528 | 11 | 60679546 |  |  | 0.643/0.7215 ** | 0.9165/0.9062 | 0.9583/0.9574 |
| cg11465943 | 11 | 60833378 |  |  | 0.4393/0.5134 ** | 0.8537/0.8447 | 0.8979/0.9074 |
| cg02661764 | 11 | 60833531 |  |  | 0.3239/0.4228 ** | 0.8786/0.8644 | 0.8485/0.8544 |
| cg24674703 | 11 | 60869960 | CD5 |  | 0.1669/0.242 ** | 0.1927/0.2108 | 0.5355/0.582 |
| cg00299736 | 11 | 60869969 | CD5 |  | 0.0877/0.1216 ** | 0.1034/0.119 | 0.2339/0.2706 |
| cg26904017 | 11 | 63634673 | MARK2 |  | 0.3545/0.4198 ** | 0.7307/0.6889 | 0.6555/0.6653 |
| cg11034978 | 11 | 64837010 |  |  | 0.212/0.167 ** | 0.116/0.1155 | 0.0319/0.0327 |
| cg24127061 | 11 | 65839402 | PACS1 |  | 0.3412/0.4261 ** | 0.5655/0.5852 | 0.5901/0.6347 |
| cg17161520 | 11 | 67174843 | TBC1D10C |  | 0.2625/0.3521 ** | 0.0597/0.09 | 0.8566/0.8771 |
| cg20234060 | 11 | 67183546 | ATPGD1 |  | 0.7084/0.7467 ** | 0.7842/0.7936 | 0.8313/0.8447 |
| cg06396265 | 11 | 67761514 | UNC93B1 |  | 0.1716/0.2486 ** | 0.0766/0.0899 | 0.303/0.3605 * |
| cg23796243 | 11 | 67978654 | SUV420H1 |  | 0.285/0.3663 ** | 0.6651/0.6565 | 0.752/0.7714 |
| cg08759026 | 11 | 69061454 | MYEOV |  | 0.3523/0.2961 ** | 0.3845/0.3409 | 0.1046/0.1017 |
| cg24344787 | 11 | 71418204 |  |  | 0.716/0.7473 ** | 0.825/0.8264 | 0.8305/0.8396 |
| cg13505393 | 11 | 76377572 | LRRC32 |  | 0.3487/0.2928 ** | 0.1218/0.1225 | 0.0508/0.0463 |
| cg01475325 | 11 | 76498701 | TSKU |  | 0.517/0.5882 ** | 0.6583/0.6726 | 0.8742/0.8753 |
| cg25625514 | 11 | 76535564 |  |  | 0.5013/0.4405 ** | 0.6237/0.5498 * | 0.6465/0.543 * |
| cg02385173 | 11 | 76571534 | ACER3 |  | 0.5965/0.6792 ** | 0.9058/0.9126 | 0.9082/0.9207 |
| cg07530172 | 11 | 95431373 |  |  | 0.4839/0.4046 ** | 0.1714/0.1752 | 0.224/0.229 |
| cg24149237 | 11 | 117687628 |  |  | 0.4558/0.3794 ** | 0.1624/0.2004 | 0.056/0.0636 |
| cg22335223 | 11 | 117698911 | FXYD2 |  | 0.3635/0.4623 ** | 0.3732/0.3973 | 0.7534/0.7722 |
| cg25155064 | 11 | 118100782 | MPZL3 |  | 0.3293/0.4505 ** | 0.8662/0.8528 | 0.9258/0.9347 |
| cg04537602 | 11 | 118763859 | CXCR5 |  | 0.3047/0.4074 ** | 0.4623/0.5249 * | 0.7437/0.7705 |
| cg13298528 | 11 | 118763863 | CXCR5 |  | 0.3232/0.432 ** | 0.4758/0.5553 * | 0.8135/0.8333 |
| cg19791714 | 11 | 118763901 | CXCR5 |  | 0.2303/0.3016 ** | 0.3189/0.3665 | 0.6417/0.6586 |
| cg15350899 | 11 | 118781763 | BCL9L |  | 0.4327/0.5212 ** | 0.8296/0.814 | 0.8796/0.8774 |
| cg20337103 | 11 | 118781778 | BCL9L |  | 0.2616/0.3431 ** | 0.7093/0.6989 | 0.7237/0.7032 |
| cg20029201 | 11 | 118781813 | BCL9L |  | 0.292/0.3985 ** | 0.7604/0.7427 | 0.864/0.8584 |
| cg02341556 | 11 | 118781978 | BCL9L |  | 0.4729/0.5423 ** | 0.7306/0.7145 | 0.6931/0.7108 |
| cg12997404 | 11 | 118977474 | C2CD2L |  | 0.1675/0.1311 ** | 0.0792/0.0765 | 0.0415/0.0383 |
| cg24223075 | 11 | 119137279 | CBL |  | 0.2988/0.388 ** | 0.8281/0.8196 | 0.8579/0.8665 |
| cg09684429 | 11 | 124768015 | ROBO4 |  | 0.4248/0.3574 ** | 0.2464/0.2388 | 0.12/0.1166 |
| cg03798942 | 11 | 128566958 | FLI1 |  | 0.2257/0.2982 ** | 0.7715/0.754 | 0.6192/0.5988 |
| cg14631438 | 11 | 128732986 | KCNJ1 |  | 0.8553/0.8651 | 0.1254/0.1742 ** | 0.9314/0.9262 |
| cg25354657 | 11 | 129991445 | APLP2 |  | 0.3674/0.4609 ** | 0.2867/0.3058 | 0.825/0.839 |
| cg00517080 | 11 | 134098583 | VPS26B |  | 0.6486/0.73 ** | 0.0383/0.0568 | 0.9732/0.9708 |
| cg24211304 | 12 | 4596426 |  |  | 0.7385/0.8063 ** | 0.9403/0.9329 | 0.9755/0.9705 |
| cg26271001 | 12 | 5313518 |  |  | 0.5556/0.6264 ** | 0.1809/0.2152 | 0.8004/0.8177 |
| cg23019886 | 12 | 6277045 |  |  | 0.2804/0.2176 ** | 0.0791/0.0852 | 0.0405/0.0379 |
| cg08418872 | 12 | 6442954 | TNFRSF1A |  | 0.6642/0.7519 ** | 0.9375/0.9303 | 0.9351/0.9293 |
| cg23752651 | 12 | 6442966 | TNFRSF1A |  | 0.7694/0.8351 ** | 0.9308/0.9224 | 0.898/0.8935 |
| cg22036538 | 12 | 6554051 | LOC678655; CD27 |  | 0.1489/0.2025 ** | 0.0475/0.0606 | 0.4943/0.5419 |
| cg00252813 | 12 | 6642229 | GAPDH |  | 0.2558/0.2021 ** | 0.161/0.1401 | 0.0349/0.035 |
| cg02519286 | 12 | 6642354 | GAPDH |  | 0.2808/0.2278 ** | 0.1555/0.1451 | 0.0789/0.0724 |
| cg09080114 | 12 | 6983111 | SPSB2 |  | 0.1588/0.1297 ** | 0.0954/0.0891 | 0.0493/0.0488 |
| cg15368872 | 12 | 10525233 | KLRK1 |  | 0.4576/0.3893 ** | 0.614/0.5996 | 0.1012/0.1108 |
| cg21747310 | 12 | 11709115 |  |  | 0.3582/0.4565 ** | 0.9065/0.8869 | 0.8927/0.8752 |
| cg00630991 | 12 | 12252249 | BCL2L14 |  | 0.6162/0.6763 ** | 0.6402/0.6538 | 0.7255/0.7492 |
| cg22221831 | 12 | 15039397 | MGP |  | 0.8118/0.7354 * | 0.8036/0.7173 ** | 0.7195/0.6072 |
| cg13302154 | 12 | 15039432 | MGP |  | 0.6445/0.5538 ** | 0.5681/0.5014 * | 0.3731/0.3101 |
| cg03499161 | 12 | 22254996 |  |  | 0.2709/0.2412 ** | 0.1494/0.1569 | 0.1533/0.1522 |
| cg07684519 | 12 | 29303184 |  |  | 0.2694/0.188 ** | 0.1296/0.1258 | 0.0689/0.0632 |
| cg08543028 | 12 | 31902503 |  |  | 0.4419/0.3479 ** | 0.6206/0.5684 | 0.0386/0.0301 |
| cg01088404 | 12 | 48214523 | HDAC7 |  | 0.3429/0.2712 ** | 0.1587/0.146 | 0.0404/0.0363 |
| cg06821460 | 12 | 49332783 | ARF3 |  | 0.6797/0.615 * | 0.1139/0.1625 ** | 0.2542/0.3034 |
| cg18399183 | 12 | 51318138 | METTL7A |  | 0.2187/0.1784 ** | 0.0589/0.061 | 0.0521/0.0496 |
| cg21253043 | 12 | 51783393 | GALNT6 |  | 0.5253/0.6165 ** | 0.833/0.8382 | 0.9155/0.9203 |
| cg22193385 | 12 | 52638005 | KRT7 |  | 0.2153/0.1583 ** | 0.2133/0.2102 | 0.0276/0.0261 |
| cg22460123 | 12 | 52638294 | KRT7 |  | 0.3238/0.2791 ** | 0.175/0.1555 | 0.1318/0.127 |
| cg24402372 | 12 | 52997366 |  |  | 0.4226/0.5264 ** | 0.9054/0.8872 | 0.9337/0.9216 |
| cg02699834 | 12 | 53039430 | KRT2 |  | 0.1149/0.1501 ** | 0.245/0.2687 | 0.2423/0.2747 |
| cg02377704 | 12 | 53075359 | KRT1 |  | 0.2122/0.2965 ** | 0.5844/0.6071 | 0.5583/0.6013 |
| cg03348792 | 12 | 53075482 | KRT1 |  | 0.1625/0.1971 ** | 0.2869/0.3001 | 0.3032/0.3122 |
| cg11606261 | 12 | 53775336 | SP1 |  | 0.4156/0.3348 ** | 0.91/0.8637 | 0.0297/0.0293 |
| cg08445469 | 12 | 53970794 | ATF7 |  | 0.5694/0.5159 ** | 0.6936/0.6684 | 0.3062/0.3011 |
| cg02710015 | 12 | 55362424 | KIAA0748 |  | 0.2572/0.3534 ** | 0.7039/0.7043 | 0.4759/0.4655 |
| cg24414325 | 12 | 56414442 | IKZF4 |  | 0.5134/0.4049 ** | 0.6833/0.6807 | 0.1555/0.1519 |
| cg00026033 | 12 | 56414490 | IKZF4 |  | 0.4807/0.4094 ** | 0.4813/0.481 | 0.0959/0.1027 |
| cg20054248 | 12 | 56414508 | IKZF4 |  | 0.2574/0.2068 ** | 0.2145/0.2069 | 0.037/0.0364 |
| cg01565774 | 12 | 56414533 | IKZF4 |  | 0.2149/0.1664 ** | 0.2082/0.2062 | 0.0487/0.0502 |
| cg01395351 | 12 | 57421088 |  |  | 0.2669/0.3167 ** | 0.4244/0.4509 | 0.4472/0.4652 |
| cg08163918 | 12 | 57628654 | SHMT2 |  | 0.8671/0.8489 | 0.2413/0.3322 ** | 0.9732/0.9728 |
| cg06015525 | 12 | 57872123 | ARHGAP9 |  | 0.2356/0.2979 ** | 0.3455/0.3551 | 0.4464/0.4573 |
| cg15016701 | 12 | 63211683 | PPM1H |  | 0.1003/0.1227 ** | 0.1421/0.1599 | 0.1795/0.21 |
| cg27292835 | 12 | 65063890 | RASSF3 |  | 0.0835/0.1091 ** | 0.1604/0.1586 | 0.2249/0.2298 |
| cg16011250 | 12 | 68024459 |  |  | 0.3776/0.4624 ** | 0.6068/0.6168 | 0.7469/0.7166 |
| cg24448421 | 12 | 68737061 |  |  | 0.1572/0.2021 ** | 0.0541/0.0663 | 0.3706/0.3985 |
| cg15085883 | 12 | 68848994 |  |  | 0.5722/0.4637 ** | 0.0825/0.0948 | 0.4043/0.3487 |
| cg02266731 | 12 | 69357333 | CPM |  | 0.4036/0.3172 ** | 0.6656/0.5989 | 0.0355/0.0312 |
| cg25886621 | 12 | 93130251 | PLEKHG7 |  | 0.4987/0.5909 ** | 0.9122/0.9127 | 0.9643/0.9635 |
| cg23436584 | 12 | 98833359 |  |  | 0.5757/0.5227 ** | 0.6144/0.5765 * | 0.5325/0.4717 |
| cg16871561 | 12 | 98986887 | SLC25A3 |  | 0.324/0.4269 ** | 0.8891/0.8726 | 0.7976/0.7912 |
| cg02927448 | 12 | 100532162 | UHRF1BP1L |  | 0.4814/0.4155 * | 0.0708/0.1052 ** | 0.052/0.0548 |
| cg02908942 | 12 | 104849615 | CHST11 |  | 0.4079/0.4803 ** | 0.2741/0.2833 | 0.8005/0.7753 |
| cg16618104 | 12 | 104853100 | CHST11 |  | 0.313/0.2428 ** | 0.0503/0.054 | 0.0609/0.0544 |
| cg06647068 | 12 | 104853274 | CHST11 |  | 0.5169/0.4249 ** | 0.2295/0.2093 | 0.1967/0.1585 |
| cg25684349 | 12 | 107725084 | BTBD11 |  | 0.2032/0.2499 ** | 0.0739/0.0789 | 0.4645/0.4568 |
| cg00159243 | 12 | 109023799 | SELPLG |  | 0.5687/0.5309 * | 0.8862/0.8402 ** | 0.4235/0.3798 |
| cg17635080 | 12 | 109030115 |  |  | 0.3621/0.4592 ** | 0.0495/0.0612 | 0.704/0.7111 |
| cg22742001 | 12 | 110435418 | GIT2 |  | 0.6587/0.5559 * | 0.0922/0.1299 ** | 0.0246/0.0223 |
| cg25668626 | 12 | 113343603 | OAS1 | IFN | 0.878/0.8077 ** | 0.7992/0.7409 | 0.9048/0.8741 |
| cg19789466 | 12 | 113344923 | OAS1 | IFN | 0.0774/0.0537 ** | 0.0635/0.0498 ** | 0.0632/0.0468 ** |
| cg04951822 | 12 | 113345598 | OAS1 | IFN | 0.247/0.1939 ** | 0.2657/0.2306 | 0.2295/0.2126 |
| cg22260958 | 12 | 113375880 | OAS3 | IFN | 0.7689/0.6989 ** | 0.747/0.6674 * | 0.7569/0.6853 |
| cg25800166 | 12 | 113375896 | OAS3 | IFN | 0.5795/0.4634 ** | 0.6453/0.5697 * | 0.5506/0.4301 * |
| cg10421247 | 12 | 120524653 | CCDC64 |  | 0.3597/0.2969 ** | 0.1713/0.1574 | 0.0508/0.0556 |
| cg06800840 | 12 | 121476549 | OASL | IFN | 0.0307/0.0273 | 0.0625/0.042 ** | 0.0559/0.0349 * |
| cg16460342 | 12 | 121662577 | P2RX4 |  | 0.1216/0.1693 ** | 0.3827/0.4107 | 0.1753/0.179 |
| cg02499214 | 12 | 122230490 | RHOF |  | 0.0495/0.0674 ** | 0.0496/0.0575 | 0.1397/0.1549 |
| cg19515108 | 12 | 123599873 |  |  | 0.5295/0.5852 ** | 0.4815/0.5074 | 0.7581/0.801 |
| cg06152215 | 12 | 124422259 | CCDC92 |  | 0.063/0.0839 ** | 0.1288/0.1288 | 0.1216/0.1485 |
| cg01230386 | 12 | 133383619 | GOLGA3 |  | 0.3839/0.5071 ** | 0.906/0.8874 | 0.9669/0.9734 |
| cg22149555 | 12 | 133410880 |  |  | 0.248/0.3296 ** | 0.6497/0.6792 | 0.7421/0.782 |
| cg15275758 | 12 | 133412815 |  |  | 0.4192/0.4918 ** | 0.6875/0.7089 | 0.7769/0.8241 |
| cg16726039 | 12 | 133413162 |  |  | 0.5838/0.6775 ** | 0.8019/0.8145 | 0.8483/0.8753 |
| cg09120938 | 12 | 133424655 | CHFR |  | 0.7873/0.8435 ** | 0.9584/0.9568 | 0.9518/0.9538 |
| cg17524886 | 12 | 133424709 | CHFR |  | 0.6614/0.7308 ** | 0.8962/0.895 | 0.9313/0.9271 |
| cg18332814 | 13 | 24247649 | TNFRSF19 |  | 0.7625/0.8288 ** | 0.904/0.9087 | 0.9672/0.96 |
| cg07756788 | 13 | 30532829 |  |  | 0.5495/0.6549 ** | 0.8724/0.8777 | 0.9417/0.9303 |
| cg14150115 | 13 | 30963379 |  |  | 0.343/0.4447 ** | 0.9336/0.9148 | 0.9004/0.8946 |
| cg25936902 | 13 | 30982971 |  |  | 0.5131/0.4359 ** | 0.7104/0.6612 | 0.0923/0.0925 |
| cg01373189 | 13 | 33002820 | N4BP2L1 |  | 0.4002/0.344 ** | 0.195/0.1813 | 0.1167/0.121 |
| cg25365958 | 13 | 42615991 |  |  | 0.4151/0.3172 ** | 0.1402/0.1466 | 0.095/0.0989 |
| cg03763873 | 13 | 43565901 | EPSTI1 |  | 0.0969/0.049 * | 0.0635/0.0439 ** | 0.0622/0.0428 |
| cg18634760 | 13 | 46679242 | CPB2 |  | 0.7295/0.6257 ** | 0.9223/0.8879 * | 0.1324/0.1104 |
| cg07880943 | 13 | 46744500 | LCP1 |  | 0.3907/0.4623 ** | 0.6246/0.6238 | 0.8705/0.8708 |
| cg25989526 | 13 | 49147573 |  |  | 0.5392/0.447 ** | 0.2326/0.256 | 0.0351/0.0286 |
| cg02043329 | 13 | 49740771 | FNDC3A |  | 0.7756/0.7 ** | 0.907/0.8811 | 0.5215/0.4929 |
| cg18412721 | 13 | 51421269 |  |  | 0.276/0.342 ** | 0.0688/0.0869 | 0.5737/0.6107 |
| cg26776551 | 13 | 51944507 | INTS6 |  | 0.622/0.5187 ** | 0.8851/0.8353 | 0.2001/0.1823 |
| cg12229775 | 13 | 74315385 | KLF12 |  | 0.4259/0.5232 ** | 0.9051/0.8966 | 0.9142/0.908 |
| cg17799287 | 13 | 92001764 | MIR19A; MIR18A; MIR17HG; MIR17 |  | 0.1754/0.128 ** | 0.0567/0.0494 | 0.041/0.0358 |
| cg23665802 | 13 | 92002338 | MIR92A1; MIR19A; MIR18A; MIR19B1; MIR17HG; MIR17; MIR20A |  | 0.4281/0.3484 ** | 0.263/0.2372 | 0.2293/0.2026 |
| cg10304534 | 13 | 95844361 | ABCC4 |  | 0.7566/0.7058 ** | 0.5088/0.4902 | 0.6049/0.5745 |
| cg10057295 | 13 | 99230004 | STK24 |  | 0.4468/0.3666 ** | 0.1429/0.1263 * | 0.0681/0.0589 |
| cg14659511 | 13 | 99668433 | DOCK9 |  | 0.4021/0.4864 ** | 0.8711/0.8634 | 0.913/0.9077 |
| cg16131748 | 13 | 99959606 | UBAC2; GPR183 |  | 0.0455/0.0633 ** | 0.0745/0.0876 | 0.0656/0.0645 |
| cg18560638 | 13 | 100008200 | MIR623; UBAC2 |  | 0.3741/0.4609 ** | 0.7647/0.7561 | 0.8529/0.8483 |
| cg06071604 | 13 | 100069110 |  |  | 0.3079/0.3688 ** | 0.7646/0.7583 | 0.892/0.8865 |
| cg15980707 | 13 | 113348722 | ATP11A |  | 0.6168/0.6813 ** | 0.8524/0.8505 | 0.8546/0.8477 |
| cg12701674 | 13 | 114908876 |  |  | 0.5234/0.5953 ** | 0.4839/0.5073 | 0.7664/0.7658 |
| cg07218880 | 13 | 115046279 | UPF3A |  | 0.2969/0.4043 ** | 0.3369/0.3411 | 0.8495/0.8733 |
| cg25268718 | 14 | 24604711 | PSME1 |  | 0.5934/0.5401 ** | 0.3726/0.353 | 0.5597/0.544 |
| cg07917901 | 14 | 35870184 |  |  | 0.6009/0.4795 ** | 0.8027/0.734 | 0.046/0.0369 |
| cg21052932 | 14 | 51342320 | ABHD12B |  | 0.1696/0.2511 ** | 0.0554/0.0745 | 0.747/0.7686 |
| cg06633081 | 14 | 55120781 | SAMD4A |  | 0.6721/0.6306 ** | 0.7029/0.6535 * | 0.6493/0.5984 |
| cg00678668 | 14 | 55221389 | SAMD4A |  | 0.5601/0.6327 ** | 0.9065/0.8938 | 0.961/0.962 |
| cg10507281 | 14 | 55230528 | SAMD4A |  | 0.8537/0.8989 ** | 0.978/0.9725 | 0.9981/0.9945 |
| cg18470780 | 14 | 59064969 |  |  | 0.4891/0.598 ** | 0.9262/0.918 | 0.959/0.9605 |
| cg02078710 | 14 | 59066094 |  |  | 0.2985/0.3812 ** | 0.5099/0.5129 | 0.8613/0.8641 |
| cg00739471 | 14 | 69415588 | ACTN1 |  | 0.469/0.5774 ** | 0.2465/0.2736 | 0.9324/0.9802 |
| cg26280976 | 14 | 69660603 |  |  | 0.6459/0.7297 ** | 0.9702/0.9697 | 0.9752/0.9756 |
| cg08280368 | 14 | 71110536 | TTC9 |  | 0.3776/0.5024 ** | 0.774/0.7934 | 0.9615/0.9562 |
| cg26217402 | 14 | 74238381 | C14orf43 |  | 0.4115/0.4883 ** | 0.1594/0.1921 | 0.9037/0.9161 |
| cg13027206 | 14 | 91866325 | CCDC88C |  | 0.1167/0.1494 ** | 0.0841/0.0942 | 0.252/0.2643 |
| cg19903805 | 14 | 92333771 | TC2N |  | 0.2059/0.2762 ** | 0.0946/0.0971 | 0.7035/0.7152 |
| cg03447547 | 14 | 94577039 | IFI27 | IFN | 0.8253/0.7465 ** | 0.8252/0.7964 | 0.8454/0.7861 |
| cg10778971 | 14 | 94577101 | IFI27 | IFN | 0.8444/0.7088 ** | 0.8622/0.797 * | 0.8501/0.7154 * |
| cg08036899 | 14 | 94577218 | IFI27 | IFN | 0.898/0.8651 ** | 0.9007/0.8724 * | 0.9274/0.9106 |
| cg24130561 | 14 | 95621734 | DICER1 |  | 0.5188/0.5988 ** | 0.9313/0.9294 | 0.9577/0.9494 |
| cg16062483 | 14 | 98444417 | C14orf64 |  | 0.3289/0.4246 ** | 0.8909/0.8839 | 0.8535/0.8584 |
| cg16278496 | 14 | 98444476 | C14orf64 |  | 0.2706/0.35 ** | 0.8634/0.8501 | 0.7941/0.7978 |
| cg08217526 | 14 | 98445245 | C14orf64 |  | 0.1945/0.2454 ** | 0.4684/0.4647 | 0.442/0.4493 |
| cg10178917 | 14 | 99665210 | BCL11B |  | 0.479/0.5432 ** | 0.8119/0.8121 | 0.8783/0.8651 |
| cg02963266 | 14 | 99681710 | BCL11B |  | 0.3416/0.4474 ** | 0.8527/0.8429 | 0.8662/0.8775 |
| cg23479730 | 14 | 99681757 | BCL11B |  | 0.5329/0.6235 ** | 0.9146/0.9114 | 0.9176/0.9139 |
| cg04166500 | 14 | 100571607 | EVL |  | 0.4442/0.5161 ** | 0.8627/0.857 | 0.9117/0.91 |
| cg16378015 | 14 | 100611694 |  |  | 0.3894/0.4524 ** | 0.7981/0.7742 | 0.6781/0.6978 |
| cg10167235 | 14 | 100807646 | WARS |  | 0.3785/0.3112 ** | 0.1813/0.1831 | 0.0493/0.0606 |
| cg21057323 | 14 | 103412980 | CDC42BPB |  | 0.1167/0.1514 ** | 0.2631/0.2725 | 0.4576/0.4708 |
| cg04987734 | 14 | 103415873 | CDC42BPB |  | 0.1866/0.2417 ** | 0.0527/0.0665 | 0.2653/0.3637 |
| cg02003183 | 14 | 103415882 | CDC42BPB |  | 0.0828/0.1306 ** | 0.0387/0.0459 | 0.1287/0.2001 |
| cg02256455 | 14 | 104165475 | KLC1; XRCC3 |  | 0.5882/0.6364 ** | 0.3293/0.3613 | 0.7598/0.7971 |
| cg00955451 | 15 | 29213640 | APBA2 |  | 0.5486/0.6174 ** | 0.8612/0.8567 | 0.9184/0.9177 |
| cg10768063 | 15 | 29213736 | APBA2 |  | 0.4854/0.565 ** | 0.5329/0.5333 | 0.7367/0.7423 |
| cg19847577 | 15 | 29213748 | APBA2 |  | 0.2236/0.2771 ** | 0.2994/0.2935 | 0.4189/0.4004 |
| cg12044210 | 15 | 29213858 | APBA2 |  | 0.6619/0.7385 ** | 0.8562/0.8467 | 0.9579/0.959 |
| cg21917349 | 15 | 29213860 | APBA2 |  | 0.4611/0.5547 ** | 0.8119/0.7952 | 0.9535/0.9457 |
| cg21702011 | 15 | 31599090 |  |  | 0.4107/0.4382 ** | 0.4881/0.4883 | 0.4962/0.4931 |
| cg27155939 | 15 | 40347163 |  |  | 0.2308/0.2793 ** | 0.0613/0.0804 * | 0.4557/0.4555 |
| cg11693709 | 15 | 40542019 | PAK6 |  | 0.6937/0.6154 ** | 0.7617/0.6882 * | 0.6524/0.5599 |
| cg12655112 | 15 | 42261154 | EHD4 |  | 0.6248/0.52 ** | 0.8353/0.7618 * | 0.112/0.131 |
| cg18696027 | 15 | 45002597 | B2M |  | 0.2094/0.1517 ** | 0.0411/0.0405 | 0.0463/0.0425 |
| cg06192883 | 15 | 52554171 | MYO5C |  | 0.1307/0.1732 ** | 0.2387/0.2733 | 0.313/0.3505 |
| cg11098259 | 15 | 58430391 | AQP9 |  | 0.5987/0.4968 ** | 0.8507/0.7891 | 0.0371/0.0291 |
| cg04192168 | 15 | 64806741 | ZNF609 |  | 0.4194/0.5277 ** | 0.9167/0.9167 | 0.9223/0.927 |
| cg08462055 | 15 | 64944023 | ZNF609 |  | 0.2163/0.2957 ** | 0.0563/0.0754 * | 0.6509/0.6886 |
| cg11200462 | 15 | 66786368 | SNAPC5 |  | 0.778/0.7007 ** | 0.9316/0.9131 | 0.4657/0.432 |
| cg17232357 | 15 | 67012832 | SMAD6 |  | 0.4627/0.3879 ** | 0.5216/0.4588 * | 0.4515/0.3566 * |
| cg05438378 | 15 | 67383736 | SMAD3 |  | 0.2892/0.2211 ** | 0.4003/0.3829 | 0.0604/0.054 |
| cg20055861 | 15 | 68055293 | MAP2K5 |  | 0.2773/0.3595 ** | 0.0492/0.0639 | 0.9111/0.9138 |
| cg06836102 | 15 | 70744534 |  |  | 0.4255/0.4499 | 0.5903/0.4984 ** | 0.8803/0.8506 |
| cg01229658 | 15 | 70924395 |  |  | 0.6755/0.5929 ** | 0.8227/0.8117 | 0.2698/0.2484 |
| cg15188939 | 15 | 72809154 | ARIH1 |  | 0.5926/0.6644 ** | 0.0958/0.1178 | 0.9367/0.9442 |
| cg02329430 | 15 | 73921385 | NPTN |  | 0.4282/0.4938 ** | 0.7209/0.7067 | 0.5888/0.5551 |
| cg10577241 | 15 | 77456283 | SGK269 |  | 0.3698/0.4615 ** | 0.9074/0.8888 | 0.9364/0.943 |
| cg23387863 | 15 | 77472416 | SGK269 |  | 0.7388/0.6857 ** | 0.8249/0.758 ** | 0.8079/0.7268 * |
| cg02489956 | 15 | 81282510 | MESDC2 |  | 0.0799/0.066 ** | 0.0483/0.05 | 0.0405/0.0384 |
| cg23536830 | 15 | 91162876 | CRTC3 |  | 0.6908/0.7597 ** | 0.9139/0.9043 | 0.9529/0.9523 |
| cg19348484 | 15 | 91413236 | FURIN |  | 0.2714/0.2043 ** | 0.4778/0.4693 | 0.0402/0.0356 |
| cg14588003 | 15 | 93361889 |  |  | 0.4947/0.5896 ** | 0.9144/0.9082 | 0.963/0.9605 |
| cg24317086 | 15 | 93423542 |  |  | 0.4447/0.5493 ** | 0.8972/0.8802 | 0.9736/0.9693 |
| cg22700246 | 15 | 99978986 |  |  | 0.2502/0.3204 ** | 0.4457/0.4066 | 0.6639/0.6727 |
| cg23911637 | 15 | 99979013 |  |  | 0.4116/0.4867 ** | 0.5121/0.4734 | 0.6646/0.701 |
| cg06272045 | 15 | 99979059 |  |  | 0.2637/0.3447 ** | 0.4463/0.4093 | 0.6565/0.6915 |
| cg01042641 | 16 | 1575979 | IFT140 |  | 0.3331/0.4139 ** | 0.4801/0.4961 | 0.756/0.7679 |
| cg01994902 | 16 | 1576069 | IFT140 |  | 0.2653/0.3141 ** | 0.346/0.3555 | 0.5396/0.5459 |
| cg27316811 | 16 | 1576146 | IFT140 |  | 0.6394/0.7687 ** | 0.9302/0.9288 | 0.9546/0.9548 |
| cg10471113 | 16 | 1587842 | TMEM204; IFT140 |  | 0.3469/0.4356 ** | 0.6202/0.6404 | 0.755/0.7924 |
| cg00305585 | 16 | 1610898 | IFT140 |  | 0.4264/0.5253 ** | 0.7966/0.7934 | 0.8048/0.8032 |
| cg06965409 | 16 | 1611973 | IFT140 |  | 0.8571/0.903 ** | 0.9305/0.9234 | 0.9268/0.9248 |
| cg04787728 | 16 | 3598600 | NLRC3 |  | 0.6662/0.6479 | 0.1219/0.1839 ** | 0.6736/0.6998 |
| cg02435083 | 16 | 8943436 |  |  | 0.594/0.4865 ** | 0.901/0.8548 | 0.194/0.1656 |
| cg07312240 | 16 | 11406995 |  |  | 0.8516/0.8096 ** | 0.9/0.8705 * | 0.9467/0.9174 |
| cg09417209 | 16 | 11715746 |  |  | 0.0906/0.1241 ** | 0.2711/0.2629 | 0.0871/0.0938 |
| cg01366941 | 16 | 12172962 | SNX29 |  | 0.3801/0.5096 ** | 0.8787/0.8626 | 0.8221/0.8238 |
| cg27550441 | 16 | 14530550 | PARN |  | 0.4034/0.4808 ** | 0.5889/0.6003 | 0.8356/0.8473 |
| cg08961793 | 16 | 28628118 | SULT1A1 |  | 0.4983/0.4273 ** | 0.5742/0.5588 | 0.1152/0.1073 |
| cg06453916 | 16 | 29690524 | QPRT |  | 0.3785/0.3192 ** | 0.1642/0.1501 | 0.1317/0.1277 |
| cg07046436 | 16 | 30663252 | PRR14 |  | 0.1404/0.1166 ** | 0.0924/0.0837 | 0.0583/0.0546 |
| cg10421029 | 16 | 30936028 | NCRNA00095; FBXL19 |  | 0.3409/0.2873 ** | 0.2047/0.1806 | 0.1156/0.1036 |
| cg10045909 | 16 | 31075842 | ZNF668 |  | 0.1411/0.19 ** | 0.3074/0.3498 | 0.3561/0.3852 |
| cg11884546 | 16 | 31366377 | ITGAX |  | 0.104/0.0773 ** | 0.0853/0.0795 | 0.0242/0.0238 |
| cg04742550 | 16 | 31366429 | ITGAX |  | 0.2922/0.2237 ** | 0.3162/0.2991 | 0.0161/0.0165 |
| cg16750777 | 16 | 50583441 | NKD1 |  | 0.6248/0.682 ** | 0.753/0.7579 | 0.8068/0.8181 |
| cg08554257 | 16 | 50730737 | NOD2 |  | 0.2956/0.2297 * | 0.3297/0.276 ** | 0.0457/0.0457 |
| cg26954174 | 16 | 50730813 | NOD2 |  | 0.2914/0.2222 ** | 0.6586/0.5713 * | 0.0398/0.0376 |
| cg04172533 | 16 | 50743027 | NOD2 |  | 0.3121/0.2522 ** | 0.3487/0.3113 | 0.0736/0.0684 |
| cg01981760 | 16 | 53737576 | RPGRIP1L; FTO |  | 0.1334/0.1127 ** | 0.0991/0.0925 | 0.0887/0.0794 |
| cg07839457 | 16 | 57023022 | NLRC5 | IFN | 0.2919/0.1809 ** | 0.579/0.3292 ** | 0.5975/0.218 ** |
| cg16411857 | 16 | 57023191 | NLRC5 | IFN | 0.1092/0.0839 ** | 0.2583/0.1456 ** | 0.2893/0.1376 ** |
| cg08958168 | 16 | 68001415 | SLC12A4 |  | 0.4371/0.4983 ** | 0.4156/0.4428 | 0.6556/0.6916 |
| cg02959006 | 16 | 68033589 | DPEP2 |  | 0.3222/0.3599 ** | 0.5105/0.5142 | 0.4222/0.4526 |
| cg10922280 | 16 | 68034227 | DPEP2 |  | 0.2413/0.299 ** | 0.0926/0.1294 * | 0.4201/0.4578 |
| cg10313337 | 16 | 68823690 | CDH1 |  | 0.8041/0.737 ** | 0.9145/0.8771 * | 0.6276/0.5348 |
| cg04703221 | 16 | 69967063 | WWP2; MIR140 |  | 0.6261/0.5226 ** | 0.601/0.5792 | 0.1048/0.0804 |
| cg00259097 | 16 | 70770604 | VAC14 |  | 0.5873/0.6667 ** | 0.8544/0.8534 | 0.9003/0.9004 |
| cg08329113 | 16 | 70771142 | VAC14 |  | 0.5595/0.6342 ** | 0.796/0.81 | 0.874/0.8993 |
| cg08045301 | 16 | 71887487 | ATXN1L |  | 0.3504/0.4176 ** | 0.0779/0.1149 * | 0.7079/0.7295 |
| cg07846061 | 16 | 74732434 | MLKL |  | 0.4457/0.3532 ** | 0.5829/0.5317 | 0.1205/0.1359 |
| cg26676129 | 16 | 84552874 |  |  | 0.6212/0.7436 ** | 0.9411/0.9337 | 0.9548/0.9578 |
| cg10121058 | 16 | 84553063 |  |  | 0.5581/0.6753 ** | 0.8754/0.8614 | 0.967/0.9571 |
| cg26912105 | 16 | 84553585 |  |  | 0.5286/0.6293 ** | 0.8553/0.8374 | 0.9194/0.9157 |
| cg15286847 | 16 | 84690433 | KLHL36 |  | 0.3455/0.4349 ** | 0.1651/0.2068 | 0.8608/0.8617 |
| cg04431002 | 16 | 84766438 | USP10 |  | 0.86/0.7992 ** | 0.8696/0.8439 | 0.7827/0.7079 |
| cg16727774 | 16 | 87958281 | CA5A |  | 0.8322/0.8478 | 0.1279/0.2152 ** | 0.9043/0.9041 |
| cg08255481 | 16 | 88103035 | BANP |  | 0.4937/0.5533 ** | 0.0615/0.0811 | 0.8163/0.8543 |
| cg03776194 | 16 | 88770966 | RNF166 |  | 0.2827/0.3617 ** | 0.6416/0.6332 | 0.6958/0.6879 |
| cg08843248 | 16 | 89009929 | CBFA2T3 |  | 0.408/0.4569 ** | 0.5597/0.5724 | 0.7231/0.7389 |
| cg26600461 | 16 | 89101757 |  |  | 0.4089/0.5016 ** | 0.7981/0.8026 | 0.8899/0.8839 |
| cg03172657 | 16 | 89163625 | ACSF3 |  | 0.1903/0.2849 ** | 0.7811/0.7758 | 0.727/0.8077 |
| cg02691035 | 16 | 89163800 | ACSF3 |  | 0.3166/0.3762 ** | 0.7443/0.7341 | 0.4636/0.5306 |
| cg06159562 | 16 | 89424793 | ANKRD11 |  | 0.309/0.4068 ** | 0.1908/0.217 | 0.7081/0.7326 |
| cg06755448 | 16 | 89791093 | ZNF276 |  | 0.6481/0.7372 ** | 0.9473/0.934 | 0.9715/0.9665 |
| cg03809021 | 16 | 89831123 | FANCA |  | 0.5974/0.5061 ** | 0.3009/0.2861 | 0.0546/0.0549 |
| cg09104284 | 16 | 90050102 | AFG3L1 |  | 0.834/0.8414 | 0.856/0.7826 ** | 0.9608/0.961 |
| cg18404811 | 17 | 403288 |  |  | 0.3081/0.3698 ** | 0.5712/0.622 | 0.5504/0.5878 |
| cg15380836 | 17 | 1553341 | RILP |  | 0.1532/0.1247 ** | 0.1764/0.1707 | 0.0461/0.0454 |
| cg06568880 | 17 | 2166583 | SMG6 |  | 0.6464/0.7176 ** | 0.9142/0.9038 | 0.9563/0.9532 |
| cg12077963 | 17 | 4079306 | ANKFY1 |  | 0.3816/0.4797 ** | 0.9136/0.9025 | 0.9259/0.9274 |
| cg13221924 | 17 | 6495080 | KIAA0753 |  | 0.7202/0.6405 ** | 0.9175/0.8892 | 0.375/0.3398 |
| cg23571857 | 17 | 6658898 | XAF1 | IFN | 0.6142/0.5312 ** | 0.6485/0.5818 * | 0.4545/0.4314 |
| cg09251764 | 17 | 6659070 | XAF1 | IFN | 0.1928/0.1417 ** | 0.2447/0.1912 ** | 0.1001/0.0865 |
| cg06085204 | 17 | 6659164 | XAF1 | IFN | 0.1017/0.0577 ** | 0.0646/0.0432 ** | 0.0406/0.0306 |
| cg14018648 | 17 | 7083015 | ASGR1 |  | 0.6067/0.5252 ** | 0.7514/0.7319 | 0.1888/0.1865 |
| cg03613649 | 17 | 7341191 | FGF11 |  | 0.7998/0.7648 ** | 0.8239/0.8017 | 0.7818/0.7642 |
| cg12699156 | 17 | 7517016 | SHBG; FXR2 |  | 0.1763/0.221 ** | 0.4855/0.4775 | 0.4559/0.4728 |
| cg00688810 | 17 | 7517138 | SHBG; FXR2 |  | 0.4455/0.5208 ** | 0.719/0.7184 | 0.9036/0.9119 |
| cg01046511 | 17 | 7742971 | KDM6B |  | 0.847/0.7999 ** | 0.9278/0.8989 | 0.6714/0.6399 |
| cg04658021 | 17 | 8056967 | PER1 |  | 0.3024/0.2332 ** | 0.5978/0.5615 | 0.0685/0.0638 |
| cg08461692 | 17 | 8481454 | MYH10 |  | 0.1953/0.2493 ** | 0.3452/0.343 | 0.4566/0.5034 |
| cg21554670 | 17 | 9967417 | GAS7 |  | 0.5819/0.5006 ** | 0.6609/0.629 | 0.1159/0.1076 |
| cg08036492 | 17 | 13976536 | COX10 |  | 0.7064/0.6015 ** | 0.9395/0.9188 | 0.251/0.1956 |
| cg12370935 | 17 | 16976475 | MPRIP |  | 0.3877/0.4542 ** | 0.8129/0.7983 | 0.9136/0.9159 |
| cg24185397 | 17 | 25659609 |  |  | 0.208/0.1707 ** | 0.1971/0.1921 | 0.0477/0.0387 |
| cg05105919 | 17 | 25958673 | LGALS9 |  | 0.0909/0.0744 ** | 0.0959/0.087 | 0.0771/0.0722 |
| cg03909504 | 17 | 25959847 | LGALS9 |  | 0.3506/0.3033 ** | 0.2864/0.2872 | 0.2741/0.2658 |
| cg19001909 | 17 | 26205940 | C17orf108 |  | 0.524/0.6236 ** | 0.974/0.9695 | 0.9705/0.9735 |
| cg01792117 | 17 | 27088227 | C17orf63 |  | 0.8215/0.7747 ** | 0.9053/0.8915 | 0.7144/0.6836 |
| cg19048010 | 17 | 28084996 | SSH2 |  | 0.3637/0.4655 ** | 0.1484/0.1695 | 0.8383/0.8342 |
| cg16563370 | 17 | 33775952 | SLFN13 |  | 0.5615/0.4662 ** | 0.1707/0.1416 | 0.1102/0.0826 |
| cg06551997 | 17 | 33815451 | SLFN12L |  | 0.1193/0.0839 ** | 0.0458/0.0421 | 0.0267/0.0246 |
| cg03526142 | 17 | 33864734 | SLFN12L |  | 0.2279/0.2711 | 0.266/0.1221 ** | 0.8419/0.7568 |
| cg07660627 | 17 | 35481970 | ACACA |  | 0.5887/0.6718 ** | 0.7905/0.8078 | 0.7303/0.817 |
| cg06684503 | 17 | 36873584 | MLLT6 |  | 0.572/0.6477 ** | 0.9011/0.8908 | 0.8747/0.8602 |
| cg18984002 | 17 | 36876514 | MLLT6 |  | 0.7653/0.8134 ** | 0.9351/0.9353 | 0.9368/0.9408 |
| cg23901967 | 17 | 36890321 | CISD3; PCGF2 |  | 0.3847/0.4577 ** | 0.5806/0.6015 | 0.8002/0.793 |
| cg10430963 | 17 | 37124558 | FBXO47 |  | 0.6589/0.7494 ** | 0.8278/0.8197 | 0.8969/0.8975 |
| cg03293732 | 17 | 38017814 | IKZF3 |  | 0.644/0.7439 ** | 0.5067/0.4929 | 0.973/0.9838 |
| cg00442282 | 17 | 38471064 | RARA |  | 0.1442/0.1184 ** | 0.0962/0.0927 | 0.0426/0.0403 |
| cg11094248 | 17 | 38494580 | RARA |  | 0.1161/0.0892 ** | 0.2614/0.2346 | 0.0401/0.0367 |
| cg17980404 | 17 | 38601676 | IGFBP4 |  | 0.4503/0.3595 ** | 0.5175/0.4799 | 0.0447/0.0439 |
| cg25330422 | 17 | 40467382 | STAT3 | IFN | 0.2283/0.2958 * | 0.2596/0.3998 ** | 0.6883/0.7277 |
| cg08090640 | 17 | 41159289 | IFI35 | IFN | 0.4713/0.4195 * | 0.5977/0.5149 ** | 0.6038/0.5309 * |
| cg14039779 | 17 | 41857714 | C17orf105; DUSP3 |  | 0.6319/0.5528 ** | 0.6891/0.6717 | 0.3448/0.3186 |
| cg00260201 | 17 | 46029581 | PRR15L |  | 0.3443/0.2689 ** | 0.1636/0.1792 | 0.0777/0.0818 |
| cg27050612 | 17 | 46133198 | NFE2L1 |  | 0.3898/0.3355 ** | 0.2786/0.2377 | 0.2149/0.2086 |
| cg05487507 | 17 | 46671861 | HOXB5; LOC404266 |  | 0.7765/0.721 ** | 0.808/0.7678 * | 0.8297/0.7892 |
| cg17839611 | 17 | 47286802 | GNGT2; ABI3 |  | 0.439/0.5173 ** | 0.7114/0.7261 | 0.8495/0.8575 |
| cg02518338 | 17 | 47929557 |  |  | 0.7509/0.8021 ** | 0.9428/0.9432 | 0.9303/0.9336 |
| cg05418719 | 17 | 54676595 |  |  | 0.3041/0.3956 ** | 0.7202/0.7367 | 0.6354/0.6727 |
| cg12379452 | 17 | 54987827 | TRIM25 |  | 0.8477/0.7571 ** | 0.7999/0.7017 * | 0.8334/0.7499 |
| cg05347965 | 17 | 55663256 | MSI2 |  | 0.4976/0.5766 ** | 0.9035/0.8877 * | 0.8582/0.8608 |
| cg02275530 | 17 | 59328313 | BCAS3 |  | 0.7276/0.6421 ** | 0.3447/0.347 | 0.4847/0.4661 |
| cg09121543 | 17 | 61774794 | LIMD2 |  | 0.2974/0.3584 ** | 0.2108/0.2051 | 0.5729/0.6458 |
| cg25061701 | 17 | 62608856 | SMURF2 |  | 0.6572/0.7349 ** | 0.943/0.9398 | 0.9784/0.9828 |
| cg26921093 | 17 | 63534688 | AXIN2 |  | 0.4278/0.5411 ** | 0.9282/0.9231 | 0.9707/0.9686 |
| cg06559756 | 17 | 65464297 | PITPNC1 |  | 0.4177/0.509 ** | 0.1077/0.128 | 0.8089/0.8221 |
| cg01993847 | 17 | 72244690 | TTYH2 |  | 0.75/0.8054 ** | 0.8208/0.8041 | 0.9658/0.965 |
| cg21015805 | 17 | 73080225 |  |  | 0.4086/0.4822 ** | 0.7514/0.7318 | 0.8083/0.8103 |
| cg08434692 | 17 | 73086066 | SLC16A5 |  | 0.6774/0.7628 ** | 0.9011/0.8947 | 0.9817/0.9877 |
| cg17581104 | 17 | 74131752 |  |  | 0.5763/0.6413 ** | 0.7774/0.7703 | 0.8798/0.8757 |
| cg26550194 | 17 | 74639928 | ST6GALNAC1 |  | 0.1037/0.1393 ** | 0.5078/0.5058 | 0.3086/0.3561 |
| cg10611580 | 17 | 75319942 | SEPT9 |  | 0.066/0.0839 ** | 0.0319/0.0334 | 0.0956/0.0959 |
| cg04661929 | 17 | 75320035 | SEPT9 |  | 0.2519/0.312 ** | 0.0783/0.0952 | 0.6758/0.668 |
| cg00871371 | 17 | 75371476 | SEPT9 |  | 0.2282/0.3015 ** | 0.1497/0.1751 | 0.4418/0.5022 |
| cg14183922 | 17 | 75371532 | SEPT9 |  | 0.2856/0.3532 ** | 0.1879/0.1841 | 0.5341/0.5545 |
| cg03152187 | 17 | 75417165 | SEPT9 |  | 0.0711/0.1006 ** | 0.1436/0.1345 | 0.0695/0.0761 |
| cg17112975 | 17 | 75431407 | SEPT9 |  | 0.2926/0.3532 ** | 0.6173/0.6367 | 0.5106/0.5322 |
| cg20557159 | 17 | 75445261 | SEPT9 |  | 0.671/0.7481 ** | 0.8848/0.8886 | 0.927/0.9291 |
| cg07324245 | 17 | 75445905 | SEPT9 |  | 0.4499/0.5424 ** | 0.5785/0.6368 * | 0.9033/0.9062 |
| cg00324097 | 17 | 75446549 | SEPT9 |  | 0.1137/0.1557 ** | 0.1066/0.1244 | 0.4421/0.4871 |
| cg06513247 | 17 | 75446661 | SEPT9 |  | 0.1539/0.2062 ** | 0.2196/0.2378 | 0.5439/0.5845 |
| cg14843920 | 17 | 75451932 | SEPT9 |  | 0.3064/0.4009 ** | 0.7924/0.7878 | 0.9107/0.8954 |
| cg14011789 | 17 | 75452044 | SEPT9 |  | 0.461/0.5654 ** | 0.8259/0.8321 | 0.9057/0.9013 |
| cg24136318 | 17 | 75454130 | SEPT9 |  | 0.3308/0.3837 ** | 0.3037/0.3178 | 0.4456/0.4886 |
| cg02482730 | 17 | 75473577 | SEPT9 |  | 0.7189/0.7827 ** | 0.9702/0.9716 | 0.8544/0.8869 |
| cg20923885 | 17 | 75473610 | SEPT9 |  | 0.3294/0.4011 ** | 0.8507/0.8428 | 0.4611/0.5174 |
| cg18278424 | 17 | 75473667 | SEPT9 |  | 0.25/0.2948 ** | 0.5615/0.5588 | 0.3705/0.4033 |
| cg11868461 | 17 | 75830800 |  |  | 0.2082/0.1562 ** | 0.1053/0.1025 | 0.0357/0.0328 |
| cg19950606 | 17 | 76121276 | TMC6 |  | 0.4512/0.5202 ** | 0.5709/0.588 | 0.4942/0.5272 |
| cg07313882 | 17 | 76121348 | TMC6 |  | 0.1957/0.2405 ** | 0.418/0.4263 | 0.3355/0.3671 |
| cg26003388 | 17 | 76129533 | TMC8; TMC6 |  | 0.6704/0.7455 ** | 0.9513/0.9444 | 0.9781/0.9786 |
| cg22833809 | 17 | 76129984 | TMC8; TMC6 |  | 0.3143/0.3687 ** | 0.1742/0.1917 | 0.5862/0.6006 |
| cg01791634 | 17 | 76130139 | TMC8 |  | 0.2234/0.295 ** | 0.0887/0.1034 | 0.7307/0.7701 |
| cg22713958 | 17 | 76976245 | LGALS3BP |  | 0.5817/0.5511 | 0.6897/0.6208 ** | 0.8258/0.7425 * |
| cg25178683 | 17 | 76976267 | LGALS3BP |  | 0.372/0.3264 * | 0.5005/0.3992 ** | 0.6036/0.454 * |
| cg11105610 | 17 | 76976352 | LGALS3BP |  | 0.6185/0.6217 | 0.7341/0.6185 ** | 0.9299/0.9029 |
| cg17836612 | 17 | 76976357 | LGALS3BP |  | 0.4759/0.4743 | 0.6446/0.5576 ** | 0.8262/0.7309 * |
| cg07121312 | 17 | 77970193 | TBC1D16 |  | 0.1262/0.1683 ** | 0.4743/0.47 | 0.0927/0.092 |
| cg11622162 | 17 | 78237543 | RNF213 |  | 0.0945/0.0831 * | 0.1266/0.0992 ** | 0.1612/0.1197 ** |
| cg16541275 | 17 | 78821754 | RPTOR |  | 0.3996/0.4451 ** | 0.7428/0.7263 | 0.8141/0.8014 |
| cg16124975 | 17 | 79938941 | ASPSCR1 |  | 0.0333/0.0429 ** | 0.0468/0.0449 | 0.0428/0.0437 |
| cg07013698 | 17 | 80174780 |  |  | 0.4231/0.3487 ** | 0.227/0.2301 | 0.0552/0.0504 |
| cg22175624 | 17 | 80829261 | TBCD |  | 0.557/0.6182 ** | 0.1716/0.1952 * | 0.8381/0.8376 |
| cg12182124 | 18 | 21451563 | LAMA3 |  | 0.1244/0.1565 ** | 0.0854/0.0855 | 0.2645/0.2493 |
| cg20937934 | 18 | 21452788 | LAMA3 |  | 0.6301/0.7041 ** | 0.9651/0.954 | 0.9795/0.9789 |
| cg13270625 | 18 | 21452819 | LAMA3 |  | 0.4768/0.5526 ** | 0.8909/0.8736 | 0.9294/0.9241 |
| cg03634729 | 18 | 21452829 | LAMA3 |  | 0.373/0.4418 ** | 0.7839/0.7622 | 0.9338/0.9305 |
| cg01152726 | 18 | 21452844 | LAMA3 |  | 0.5839/0.6782 ** | 0.9615/0.9509 | 0.9996/0.9939 |
| cg26485825 | 18 | 21452895 | LAMA3 |  | 0.4301/0.5376 ** | 0.8859/0.8625 | 0.9691/0.9651 |
| cg06603074 | 18 | 60192893 | ZCCHC2 |  | 0.3009/0.258 * | 0.3702/0.2843 * | 0.5035/0.3734 ** |
| cg16762684 | 18 | 74820493 | MBP |  | 0.0977/0.0817 * | 0.1473/0.1146 ** | 0.0247/0.0244 |
| cg14663914 | 19 | 827739 | AZU1 |  | 0.5/0.4306 ** | 0.6766/0.6597 | 0.0379/0.0338 |
| cg02324006 | 19 | 1080034 | HMHA1 |  | 0.5704/0.6524 ** | 0.8919/0.8797 | 0.9326/0.9357 |
| cg23866916 | 19 | 1155738 | SBNO2 |  | 0.7229/0.6597 ** | 0.8279/0.8022 | 0.5263/0.4889 |
| cg27073066 | 19 | 2169160 | DOT1L |  | 0.3599/0.2803 ** | 0.1082/0.1028 | 0.0318/0.0305 |
| cg03882382 | 19 | 4540065 | LRG1 |  | 0.2998/0.2435 ** | 0.3649/0.3559 | 0.0705/0.0695 |
| cg17714703 | 19 | 4912221 | UHRF1 |  | 0.0764/0.1206 ** | 0.1014/0.1087 | 0.2909/0.3728 |
| cg12110801 | 19 | 5992284 |  |  | 0.6581/0.5645 ** | 0.5958/0.6134 | 0.2517/0.2371 |
| cg24876035 | 19 | 7682769 | KIAA1543 |  | 0.956/0.9338 ** | 0.9249/0.8908 | 0.8852/0.8223 |
| cg02319986 | 19 | 8568712 | PRAM1 |  | 0.7164/0.6475 ** | 0.891/0.8469 * | 0.5767/0.5302 |
| cg22642495 | 19 | 10197856 | C19orf66 |  | 0.0914/0.0673 ** | 0.1931/0.1304 ** | 0.1677/0.1061 * |
| cg13419792 | 19 | 10197996 | C19orf66 |  | 0.8954/0.8513 ** | 0.8411/0.8076 | 0.8809/0.8447 * |
| cg03722295 | 19 | 10519375 |  |  | 0.1675/0.2185 ** | 0.3741/0.402 | 0.4263/0.5034 |
| cg15647311 | 19 | 12897519 |  |  | 0.2939/0.249 ** | 0.0542/0.0568 | 0.0412/0.0395 |
| cg25737313 | 19 | 12899557 |  |  | 0.2417/0.1825 ** | 0.0389/0.0396 | 0.032/0.0322 |
| cg04334723 | 19 | 13054427 | CALR |  | 0.5882/0.5191 ** | 0.6847/0.6631 | 0.274/0.2395 |
| cg07839313 | 19 | 17514600 | BST2 | IFN | 0.5056/0.4346 ** | 0.516/0.4425 ** | 0.3488/0.2648 |
| cg12090003 | 19 | 17516282 | BST2 | IFN | 0.0644/0.0416 ** | 0.0247/0.0249 | 0.0246/0.0217 |
| cg16363586 | 19 | 17516329 | BST2 | IFN | 0.2421/0.1624 ** | 0.0894/0.071 ** | 0.0561/0.0428 |
| cg11558551 | 19 | 17516442 | BST2 | IFN | 0.0419/0.0297 ** | 0.0202/0.0213 | 0.0187/0.0171 |
| cg01254505 | 19 | 17516470 | BST2 | IFN | 0.0587/0.0405 ** | 0.0259/0.0255 | 0.0258/0.0241 |
| cg01329005 | 19 | 17516712 | BST2 | IFN | 0.1312/0.0785 ** | 0.0459/0.0399 * | 0.0361/0.0277 |
| cg09993699 | 19 | 17517008 | BST2 | IFN | 0.1359/0.0876 ** | 0.0707/0.0561 * | 0.043/0.0364 |
| cg20092122 | 19 | 17517221 | BST2 | IFN | 0.0975/0.0569 ** | 0.033/0.0308 | 0.0308/0.0269 |
| cg27384695 | 19 | 19271486 | LOC729991-MEF2B; MEF2B |  | 0.174/0.2209 ** | 0.0405/0.0507 | 0.2169/0.2518 |
| cg12103219 | 19 | 30165308 | PLEKHF1 |  | 0.8432/0.908 ** | 0.9719/0.9692 | 0.98/0.9787 |
| cg19977428 | 19 | 35819985 | CD22 |  | 0.28/0.2221 ** | 0.0465/0.0504 | 0.0347/0.0328 |
| cg25289028 | 19 | 36428519 | LRFN3 |  | 0.4198/0.5411 ** | 0.8429/0.8411 | 0.961/0.9694 |
| cg04790874 | 19 | 42381950 | CD79A |  | 0.4405/0.3565 ** | 0.0387/0.0473 | 0.1773/0.1705 |
| cg17769442 | 19 | 45578863 | ZNF296 |  | 0.1285/0.1014 ** | 0.0483/0.0492 | 0.051/0.0474 |
| cg00763834 | 19 | 45927335 | ERCC1 |  | 0.056/0.0475 ** | 0.0426/0.0417 | 0.0307/0.0288 |
| cg22810489 | 19 | 45927585 | ERCC1 |  | 0.3302/0.2773 ** | 0.316/0.2808 | 0.0913/0.0759 |
| cg05492306 | 19 | 45927594 | ERCC1 |  | 0.3919/0.3232 ** | 0.357/0.3151 | 0.0712/0.0609 |
| cg05286653 | 19 | 46800602 | HIF3A |  | 0.3015/0.2535 ** | 0.3081/0.2625 * | 0.2348/0.2027 |
| cg25607249 | 19 | 47288039 | SLC1A5 |  | 0.2424/0.1835 ** | 0.2515/0.2042 * | 0.0471/0.0427 |
| cg21766592 | 19 | 47288066 | SLC1A5 |  | 0.2045/0.1484 ** | 0.1994/0.1565 * | 0.0554/0.043 |
| cg12165685 | 19 | 47288109 | SLC1A5 |  | 0.1231/0.0959 ** | 0.1664/0.1331 | 0.0409/0.0395 |
| cg11645155 | 19 | 47288114 | SLC1A5 |  | 0.0689/0.0525 ** | 0.0754/0.0687 | 0.0281/0.0262 |
| cg01406381 | 19 | 47288263 | SLC1A5 |  | 0.1935/0.1401 ** | 0.1517/0.1227 | 0.0194/0.018 |
| cg23314866 | 19 | 48016020 | NAPA |  | 0.3372/0.3062 * | 0.4441/0.4107 ** | 0.1734/0.1411 |
| cg05262463 | 19 | 50056081 |  |  | 0.3246/0.4481 ** | 0.8547/0.849 | 0.8327/0.8698 |
| cg18734095 | 19 | 50062005 | NOSIP |  | 0.2576/0.3521 ** | 0.8034/0.7915 | 0.7287/0.7274 |
| cg22820233 | 19 | 55385581 | FCAR |  | 0.674/0.5586 ** | 0.8994/0.872 | 0.0765/0.068 |
| cg09592958 | 20 | 207161 | DEFB129 |  | 0.4291/0.5367 ** | 0.8366/0.8269 | 0.9036/0.9041 |
| cg20066792 | 20 | 4664695 |  |  | 0.3978/0.4952 ** | 0.8278/0.8662 | 0.7118/0.7171 |
| cg01450566 | 20 | 9484340 |  |  | 0.5865/0.5262 ** | 0.6752/0.6511 | 0.2736/0.2937 |
| cg22052056 | 20 | 31351813 | DNMT3B |  | 0.7551/0.6711 ** | 0.6621/0.6312 | 0.5059/0.4608 |
| cg26347170 | 20 | 32441424 | CHMP4B |  | 0.4102/0.5183 ** | 0.9212/0.9182 | 0.9491/0.9519 |
| cg05971521 | 20 | 35584019 |  |  | 0.3158/0.3813 ** | 0.517/0.4912 | 0.6069/0.579 |
| cg00732815 | 20 | 44636981 | MMP9 |  | 0.1482/0.1137 ** | 0.0854/0.0813 | 0.0604/0.0553 |
| cg06122230 | 20 | 47887219 | ZNFX1 |  | 0.7508/0.6626 ** | 0.9481/0.9278 | 0.3421/0.2905 |
| cg02828104 | 20 | 48770800 | TMEM189; TMEM189-UBE2V1 |  | 0.359/0.4259 ** | 0.5602/0.5785 | 0.617/0.6335 |
| cg12831034 | 20 | 57582971 | CTSZ |  | 0.1993/0.1609 ** | 0.0338/0.0364 | 0.0291/0.0285 |
| cg15262954 | 20 | 62198872 | PRIC285 |  | 0.123/0.1758 * | 0.0308/0.0666 ** | 0.0241/0.029 |
| cg06064964 | 20 | 62199181 | PRIC285 |  | 0.0883/0.1376 ** | 0.0326/0.0369 | 0.0321/0.0324 |
| cg09844573 | 20 | 62199190 | PRIC285 |  | 0.1216/0.173 ** | 0.046/0.0509 | 0.0443/0.0445 |
| cg01190666 | 20 | 62204908 | PRIC285 |  | 0.6041/0.456 ** | 0.4994/0.3762 ** | 0.4727/0.3089 ** |
| cg06295590 | 20 | 62205981 | PRIC285 |  | 0.2001/0.1653 ** | 0.1329/0.1204 | 0.1239/0.1019 |
| cg03227037 | 20 | 62212228 |  |  | 0.3167/0.4073 ** | 0.3455/0.4018 * | 0.3051/0.4096 * |
| cg11284582 | 20 | 62270540 |  |  | 0.2071/0.2569 ** | 0.0849/0.1099 | 0.4388/0.4472 |
| cg14977069 | 20 | 62367698 | LIME1 |  | 0.186/0.2572 ** | 0.105/0.1199 | 0.5379/0.6209 |
| cg06653796 | 20 | 62367805 | LIME1 |  | 0.0865/0.1318 ** | 0.0329/0.041 | 0.2906/0.3563 |
| cg00446123 | 20 | 62367888 | LIME1 |  | 0.2091/0.3022 ** | 0.053/0.0717 | 0.7255/0.7921 |
| cg13277040 | 20 | 62716332 | OPRL1; C20orf201 |  | 0.4723/0.4422 ** | 0.4424/0.434 | 0.3142/0.3154 |
| cg21291385 | 21 | 35448215 | SLC5A3; MRPS6 |  | 0.1642/0.2207 ** | 0.7006/0.6473 | 0.4322/0.4176 |
| cg15683970 | 21 | 35747081 | FAM165B |  | 0.4197/0.3508 ** | 0.5758/0.5615 | 0.0646/0.0657 |
| cg21258596 | 21 | 38338458 | HLCS |  | 0.1382/0.1121 ** | 0.0641/0.067 | 0.0727/0.0676 |
| cg19788186 | 21 | 39669558 | KCNJ15 |  | 0.5326/0.6342 ** | 0.1504/0.1718 | 0.8924/0.8966 |
| cg16785077 | 21 | 42791867 | MX1 | IFN | 0.7702/0.6702 ** | 0.8529/0.8062 * | 0.5967/0.4892 |
| cg13507964 | 21 | 42792703 | MX1 | IFN | 0.0482/0.0341 ** | 0.0324/0.0299 | 0.0325/0.0301 |
| cg22862003 | 21 | 42797588 | MX1 | IFN | 0.6159/0.3444 ** | 0.5932/0.3248 ** | 0.5506/0.171 ** |
| cg26312951 | 21 | 42797847 | MX1 | IFN | 0.2116/0.1138 ** | 0.2438/0.1187 ** | 0.2632/0.048 ** |
| cg12359279 | 21 | 42797953 | MX1 | IFN | 0.0232/0.0194 * | 0.0276/0.0243 | 0.0352/0.0246 ** |
| cg08924203 | 21 | 42798747 | MX1 | IFN | 0.1026/0.0534 ** | 0.1071/0.0631 ** | 0.1125/0.0606 * |
| cg21549285 | 21 | 42799141 | MX1 | IFN | 0.5598/0.21 ** | 0.6305/0.2417 ** | 0.6554/0.0764 ** |
| cg01881899 | 21 | 43652704 | ABCG1 |  | 0.0885/0.1171 ** | 0.0383/0.0389 | 0.2579/0.235 |
| cg23732182 | 21 | 44898090 | C21orf84 |  | 0.6416/0.5781 ** | 0.6602/0.6421 | 0.4878/0.4566 |
| cg16334524 | 21 | 44898123 | C21orf84 |  | 0.6545/0.588 ** | 0.6438/0.6119 | 0.5262/0.4939 |
| cg21913632 | 21 | 46505130 | ADARB1 |  | 0.32/0.4035 ** | 0.291/0.328 | 0.7988/0.7881 |
| cg22517527 | 21 | 48056967 | PRMT2 |  | 0.3686/0.4859 ** | 0.9239/0.9149 | 0.9638/0.957 |
| cg10685559 | 22 | 18633123 | USP18 | IFN | 0.0427/0.0322 ** | 0.0458/0.0315 ** | 0.044/0.0332 |
| cg14293575 | 22 | 18635460 | USP18 | IFN | 0.4823/0.2401 ** | 0.582/0.251 ** | 0.7075/0.3913 * |
| cg26354221 | 22 | 24822802 | ADORA2A |  | 0.7859/0.8439 ** | 0.9367/0.9371 | 0.9518/0.9514 |
| cg26001125 | 22 | 24823050 | ADORA2A |  | 0.4724/0.5462 ** | 0.3796/0.3862 | 0.8942/0.8953 |
| cg15499799 | 22 | 24823110 | ADORA2A |  | 0.604/0.6753 ** | 0.7467/0.754 | 0.9239/0.9235 |
| cg04250930 | 22 | 24823141 | ADORA2A |  | 0.6857/0.7455 ** | 0.4067/0.4378 | 0.8786/0.8716 |
| cg04990420 | 22 | 24824362 | ADORA2A |  | 0.2283/0.2961 ** | 0.4738/0.4707 | 0.6069/0.6318 |
| cg05543864 | 22 | 24979755 | GGT1 |  | 0.7086/0.6645 ** | 0.7268/0.6871 ** | 0.7007/0.6302 * |
| cg22764925 | 22 | 24979964 | GGT1 |  | 0.67/0.5708 ** | 0.6849/0.5859 ** | 0.685/0.5048 ** |
| cg00539347 | 22 | 30592296 |  |  | 0.2675/0.196 ** | 0.0864/0.0827 | 0.0166/0.0173 |
| cg11564601 | 22 | 30592435 |  |  | 0.3834/0.2892 ** | 0.3633/0.3395 | 0.085/0.0766 |
| cg25739715 | 22 | 30663881 | OSM |  | 0.0307/0.0303 | 0.2258/0.1802 ** | 0.0411/0.0376 |
| cg23072383 | 22 | 31031044 | SLC35E4 |  | 0.2153/0.1624 ** | 0.0386/0.0368 | 0.0276/0.0285 |
| cg23825480 | 22 | 31336785 | MORC2 |  | 0.2052/0.3007 ** | 0.8377/0.8431 | 0.8111/0.8291 |
| cg08612539 | 22 | 37257124 | NCF4 |  | 0.2024/0.1536 ** | 0.0314/0.0332 | 0.0312/0.0316 |
| cg21345826 | 22 | 39353650 | APOBEC3A |  | 0.7028/0.6202 ** | 0.8472/0.8568 | 0.4964/0.4529 |
| cg03318904 | 22 | 39801522 | MAP3K7IP1 |  | 0.5134/0.5955 ** | 0.9103/0.897 | 0.8596/0.8775 |
| cg26815454 | 22 | 40296767 | GRAP2 |  | 0.1336/0.1798 ** | 0.3797/0.3792 | 0.4426/0.4646 |
| cg04136484 | 22 | 40589892 | TNRC6B |  | 0.3346/0.3726 ** | 0.6315/0.6234 | 0.5241/0.5252 |
| cg04630823 | 22 | 45575276 | NUP50 |  | 0.1868/0.2417 ** | 0.7174/0.6941 | 0.4984/0.5064 |
| cg01234420 | 22 | 46453808 | LOC150381 |  | 0.6702/0.5665 ** | 0.4672/0.4093 * | 0.416/0.3353 |
| cg22982767 | 22 | 46454012 | LOC150381 |  | 0.0887/0.0697 ** | 0.0318/0.0306 | 0.0282/0.0235 |
| cg03834031 | 22 | 46465717 |  |  | 0.3063/0.2435 ** | 0.2487/0.201 * | 0.0633/0.0533 |
| cg00218103 | 22 | 46465977 |  |  | 0.127/0.093 ** | 0.0609/0.0546 | 0.0387/0.0367 |
| cg09137533 | 22 | 46469091 |  |  | 0.4893/0.4064 ** | 0.4323/0.3826 | 0.3931/0.339 |
| cg03672272 | 22 | 46470191 |  |  | 0.7367/0.6769 ** | 0.7145/0.6941 | 0.6215/0.5774 |
| cg21560830 | 22 | 49879782 |  |  | 0.8312/0.7836 ** | 0.8937/0.8708 | 0.7664/0.6813 |
| cg06865642 | 22 | 50174028 | LOC90834; BRD1 |  | 0.7447/0.6641 ** | 0.9375/0.8886 * | 0.5753/0.5452 |
| cg13092901 | 22 | 50965373 | TYMP; SCO2 |  | 0.2021/0.1348 ** | 0.1139/0.0798 ** | 0.0554/0.0534 |
| cg10416593 | 22 | 50966123 | TYMP; SCO2 |  | 0.6716/0.5673 ** | 0.7665/0.7311 | 0.2827/0.2594 |
| cg23221113 | 22 | 50970943 | ODF3B |  | 0.1115/0.0632 ** | 0.0482/0.0348 ** | 0.0266/0.0247 |
| cg11224765 | 22 | 50971109 | ODF3B |  | 0.3229/0.2043 ** | 0.2053/0.1349 ** | 0.0943/0.0665 |
| cg20098015 | 22 | 50971140 | ODF3B |  | 0.4261/0.2891 ** | 0.4295/0.2755 ** | 0.2475/0.1079 * |
| cg16644494 | 22 | 50971601 | ODF3B |  | 0.8037/0.7344 ** | 0.8652/0.8304 | 0.6351/0.4929 * |
| cg05523603 | 22 | 50973101 |  |  | 0.6443/0.4879 ** | 0.6724/0.5829 | 0.4502/0.2526 * |
| cg02247863 | 22 | 50983415 |  |  | 0.7784/0.7098 ** | 0.8282/0.7901 * | 0.7439/0.6518 * |
| cg18770635 | 22 | 50984368 |  |  | 0.75/0.6503 ** | 0.8388/0.7958 * | 0.6756/0.5066 * |
| cg07596065 | 22 | 50984393 |  |  | 0.6402/0.5506 ** | 0.7426/0.7173 | 0.5429/0.4378 * |
| cg20494841 | 22 | 50984597 |  |  | 0.7411/0.6668 ** | 0.7874/0.7639 | 0.6799/0.5796 |
| cg07833467 | 22 | 50986511 | KLHDC7B |  | 0.4816/0.3563 ** | 0.244/0.1873 | 0.4588/0.2998 |
| cg18533225 | 22 | 50986813 | KLHDC7B |  | 0.4315/0.3551 ** | 0.3658/0.2986 * | 0.442/0.3009 * |
| cg11829870 | 22 | 50988451 | KLHDC7B |  | 0.7418/0.6513 ** | 0.8194/0.791 | 0.6448/0.5402 |

Table of CpGs with p-values <1 x 10^-8^ in T-cells, B-cells or monocytes. The columns for each cell type indicate mean methylation proportion after correction for all covariates in controls/SLE patients. Highly significant effects (p < 1 x 10^-8^) have double asterisks. Mildly significant (FDR<1%) have single asterisks.
