# Supplementary material for: Genome-Wide DNA Methylation Analysis of Systemic Lupus Erythematosus Reveals Persistent Hypomethylation of Interferon Genes and Compositional Changes to CD4+ T-cell Populations
Source: PLoS Genet. 2013 Aug 8;9(8):e1003678. doi: 10.1371/journal.pgen.1003678 (PMC3738443; doi:10.1371/journal.pgen.1003678)
Supplement: Table S4 — List of CpGs near 42 genes with aberrant methylation in more than one cell type. Table of CpGs near genes with at least one CpG at 1×10^−8^ in one cell type and at least one CpG at FDR<1% in at least one other cell-type. The columns for each cell type indicate mean methylation proportion after correction for all covariates in controls/SLE patients. Highly significant effects (p<1×10^−8^) have double asterisks. Moderately significant (FDR<1%) have single asterisks. (DOCX) [file pgen.1003678.s008.docx]

**Table S4. List of CpGs Near 42 Genes with Aberrant Methylation in More than One Cell Type.**

| **CpG** | **Chr** | **Pos** | **Genes** | **IFN** | **CD4** | **CD19** | **CD14** |
| --- | --- | --- | --- | --- | --- | --- | --- |
| cg20062691 | 1 | 949392 | ISG15 | IFN | 0.6564/0.5262 ** | 0.744/0.6047 ** | 0.8137/0.6689 * |
| cg03811829 | 1 | 949449 | ISG15 | IFN | 0.6993/0.6041 * | 0.811/0.7301 ** | 0.8257/0.7156 * |
| cg11211792 | 1 | 949634 | ISG15 | IFN | 0.6879/0.6065 * | 0.8019/0.7234 ** | 0.8332/0.7625 |
| cg04788999 | 1 | 949850 | ISG15 | IFN | 0.5952/0.4748 ** | 0.6705/0.5732 ** | 0.6981/0.5785 * |
| cg16526047 | 1 | 949893 | ISG15 | IFN | 0.4901/0.3854 ** | 0.5689/0.4595 ** | 0.637/0.4974 * |
| cg09363892 | 1 | 954619 | AGRN |  | 0.1352/0.1531 * | 0.1638/0.2269 ** | 0.1448/0.1997 * |
| cg03185752 | 1 | 954631 | AGRN |  | 0.096/0.1066 | 0.0966/0.1291 ** | 0.0919/0.1236 |
| cg17801765 | 1 | 958274 | AGRN |  | 0.6909/0.7461 * | 0.6972/0.7534 * | 0.7154/0.8037 * |
| cg13100600 | 1 | 958351 | AGRN |  | 0.4301/0.4968 * | 0.4086/0.4762 * | 0.4785/0.5917 * |
| cg07787977 | 1 | 962651 | AGRN |  | 0.6502/0.6919 * | 0.5973/0.6561 | 0.7233/0.8003 |
| cg12140144 | 1 | 2984275 | FLJ42875; PRDM16 |  | 0.0583/0.0854 * | 0.1071/0.1413 | 0.0688/0.1069 |
| cg20236995 | 1 | 3071963 | PRDM16 |  | 0.2945/0.2728 * | 0.3153/0.309 | 0.2402/0.2235 |
| cg09321238 | 1 | 3072001 | PRDM16 |  | 0.2541/0.2337 * | 0.2776/0.2646 | 0.1888/0.1845 |
| cg01104489 | 1 | 3072235 | PRDM16 |  | 0.4818/0.4491 * | 0.4298/0.4316 | 0.409/0.3935 |
| cg24460544 | 1 | 3085446 | PRDM16 |  | 0.2032/0.2288 * | 0.1247/0.1415 | 0.3625/0.3786 |
| cg26435254 | 1 | 3087999 | PRDM16 |  | 0.1934/0.1721 * | 0.1391/0.1382 | 0.0431/0.0407 |
| cg20699701 | 1 | 3089762 | PRDM16 |  | 0.3688/0.3406 * | 0.3508/0.3505 | 0.1349/0.16 |
| cg17445936 | 1 | 3090345 | PRDM16 |  | 0.2473/0.2793 | 0.2339/0.2967 * | 0.139/0.2415 |
| cg07902789 | 1 | 3098629 | PRDM16 |  | 0.1741/0.1476 * | 0.2238/0.2285 | 0.0667/0.0663 |
| cg13393782 | 1 | 3099804 | PRDM16 |  | 0.473/0.4389 * | 0.3846/0.3798 | 0.3517/0.3514 |
| cg22860172 | 1 | 3099917 | PRDM16 |  | 0.7794/0.7528 * | 0.8154/0.8029 | 0.668/0.6672 |
| cg23689722 | 1 | 3100956 | PRDM16 |  | 0.5894/0.5367 * | 0.4266/0.4488 | 0.2806/0.3101 |
| cg10493186 | 1 | 3134756 | PRDM16 |  | 0.4422/0.4487 | 0.4119/0.4472 | 0.1545/0.2597 * |
| cg15605124 | 1 | 3230250 | PRDM16 |  | 0.7854/0.7463 ** | 0.8365/0.8166 | 0.756/0.729 |
| cg16394551 | 1 | 3230357 | PRDM16 |  | 0.2644/0.2108 * | 0.2545/0.224 | 0.1782/0.1397 |
| cg15491247 | 1 | 3230424 | PRDM16 |  | 0.3712/0.301 * | 0.3445/0.2755 * | 0.1298/0.1 |
| cg21848084 | 1 | 3264381 | PRDM16 |  | 0.3631/0.3963 * | 0.2804/0.353 ** | 0.3122/0.3796 |
| cg23050300 | 1 | 3281321 | PRDM16 |  | 0.1604/0.1872 * | 0.1288/0.1546 | 0.2209/0.247 |
| cg01261194 | 1 | 3340124 | PRDM16 |  | 0.4677/0.4295 * | 0.3811/0.3751 | 0.2997/0.3103 |
| cg03748986 | 1 | 3340187 | PRDM16 |  | 0.3553/0.3272 * | 0.3425/0.3386 | 0.3184/0.3176 |
| cg13304609 | 1 | 79085162 | IFI44L | IFN | 0.8625/0.6636 ** | 0.8779/0.6748 ** | 0.721/0.3671 ** |
| cg06872964 | 1 | 79085250 | IFI44L | IFN | 0.462/0.2585 ** | 0.5915/0.3136 ** | 0.4975/0.1554 ** |
| cg03607951 | 1 | 79085586 | IFI44L | IFN | 0.5923/0.3264 ** | 0.6037/0.3107 ** | 0.3572/0.1 * |
| cg17980508 | 1 | 79085713 | IFI44L | IFN | 0.5238/0.3053 ** | 0.4951/0.316 ** | 0.2256/0.1069 * |
| cg00855901 | 1 | 79085765 | IFI44L | IFN | 0.3132/0.1541 ** | 0.2848/0.1544 ** | 0.0441/0.0316 |
| cg05696877 | 1 | 79088769 | IFI44L | IFN | 0.5582/0.3247 ** | 0.7181/0.3958 ** | 0.6878/0.2886 ** |
| cg00458211 | 1 | 79111320 | IFI44L | IFN | 0.8243/0.76 * | 0.8373/0.8108 | 0.8132/0.6774 * |
| cg07107453 | 1 | 79114976 | IFI44 | IFN | 0.431/0.3787 * | 0.5447/0.5055 | 0.4298/0.3423 |
| cg01079652 | 1 | 79118191 | IFI44 | IFN | 0.7437/0.5309 ** | 0.8273/0.6453 ** | 0.7095/0.3486 ** |
| cg05768419 | 1 | 174417967 | GPR52; RABGAP1L |  | 0.8533/0.8152 * | 0.825/0.8043 | 0.7954/0.7855 |
| cg02482603 | 1 | 174843754 | RABGAP1L |  | 0.3844/0.3041 ** | 0.2167/0.1803 * | 0.2602/0.2078 |
| cg05702218 | 1 | 174843909 | RABGAP1L |  | 0.3423/0.2691 ** | 0.2076/0.1754 | 0.1925/0.1706 |
| cg04858586 | 1 | 174843971 | RABGAP1L |  | 0.1841/0.1603 * | 0.1118/0.1049 | 0.0844/0.0827 |
| cg13130398 | 1 | 174844397 | RABGAP1L |  | 0.8388/0.7189 ** | 0.7722/0.6008 ** | 0.853/0.6473 ** |
| cg07285983 | 1 | 174844490 | RABGAP1L |  | 0.7489/0.6105 ** | 0.7092/0.4618 ** | 0.8035/0.5472 ** |
| cg00300216 | 2 | 6992700 | CMPK2 |  | 0.7112/0.7565 * | 0.7663/0.8004 | 0.9288/0.9282 |
| cg01028142 | 2 | 7004578 | CMPK2 |  | 0.7915/0.5247 ** | 0.8164/0.5701 ** | 0.7155/0.3683 ** |
| cg24935042 | 2 | 7006627 | CMPK2 |  | 0.0649/0.0355 ** | 0.0443/0.0337 * | 0.0285/0.0222 |
| cg23213327 | 2 | 7016509 | RSAD2 |  | 0.6145/0.5015 * | 0.3183/0.2127 ** | 0.1211/0.0861 |
| cg15346781 | 2 | 7017571 | RSAD2 |  | 0.2693/0.2109 ** | 0.2999/0.2344 * | 0.1976/0.1482 |
| cg10959651 | 2 | 7018020 | RSAD2 |  | 0.1878/0.114 ** | 0.3728/0.2302 ** | 0.2625/0.1151 * |
| cg10549986 | 2 | 7018153 | RSAD2 |  | 0.1694/0.0773 ** | 0.4787/0.2439 ** | 0.1551/0.0377 |
| cg24523650 | 2 | 37381088 | EIF2AK2 | IFN | 0.5323/0.4428 ** | 0.6237/0.5952 | 0.5284/0.4417 |
| cg17326313 | 2 | 37383568 | EIF2AK2 | IFN | 0.1693/0.0737 ** | 0.0929/0.0585 ** | 0.0355/0.0341 |
| cg16795804 | 2 | 37384523 | EIF2AK2 | IFN | 0.3261/0.2502 ** | 0.2429/0.2114 | 0.2382/0.1976 |
| cg14126601 | 2 | 37384708 | EIF2AK2 | IFN | 0.552/0.4173 ** | 0.2895/0.1881 ** | 0.4918/0.4091 * |
| cg05691004 | 2 | 201170426 | SPATS2L |  | 0.857/0.8214 * | 0.8585/0.8376 | 0.8951/0.8663 |
| cg03997390 | 2 | 201170790 | SPATS2L |  | 0.0993/0.0675 * | 0.0473/0.0457 | 0.0394/0.0354 |
| cg18609578 | 2 | 201170806 | SPATS2L |  | 0.2061/0.1414 * | 0.0664/0.0578 | 0.0573/0.0544 |
| cg08153883 | 2 | 201170809 | SPATS2L |  | 0.2516/0.1874 ** | 0.0907/0.0851 | 0.0577/0.0603 |
| cg08866865 | 2 | 201173897 | SPATS2L |  | 0.9295/0.8966 * | 0.9256/0.9016 | 0.9709/0.9715 |
| cg14106933 | 2 | 201194453 | SPATS2L |  | 0.6146/0.6927 ** | 0.466/0.4887 | 0.8632/0.8688 |
| cg06226703 | 2 | 201242945 | SPATS2L |  | 0.8739/0.7912 ** | 0.8403/0.7799 * | 0.919/0.9091 |
| cg13144059 | 2 | 201245077 | SPATS2L |  | 0.855/0.7915 * | 0.8179/0.7212 * | 0.9568/0.951 |
| cg03035167 | 2 | 201336269 | SPATS2L |  | 0.5635/0.6029 | 0.4265/0.4119 | 0.8766/0.9275 * |
| cg22930808 | 3 | 122281881 | DTX3L; PARP9 |  | 0.6826/0.3521 ** | 0.71/0.3511 ** | 0.5913/0.1628 ** |
| cg08122652 | 3 | 122281939 | DTX3L; PARP9 |  | 0.6809/0.3991 ** | 0.6242/0.3325 ** | 0.7143/0.3031 ** |
| cg00959259 | 3 | 122281975 | DTX3L; PARP9 |  | 0.5046/0.2325 ** | 0.3983/0.1833 ** | 0.4932/0.1619 ** |
| cg24087515 | 3 | 122284215 | DTX3L; PARP9 |  | 0.5821/0.5303 * | 0.5403/0.4748 * | 0.6295/0.567 * |
| cg00633969 | 3 | 122284550 | DTX3L; PARP9 |  | 0.9713/0.9498 * | 0.9576/0.93 * | 0.945/0.9179 |
| cg25932713 | 3 | 122284563 | DTX3L; PARP9 |  | 0.9603/0.9479 | 0.9769/0.9509 * | 0.9613/0.9521 |
| cg14098385 | 3 | 122285010 | DTX3L; PARP9 |  | 0.9125/0.8934 * | 0.8999/0.8688 | 0.9417/0.9259 |
| cg02519879 | 3 | 122286677 | DTX3L |  | 0.5959/0.5932 | 0.8483/0.8156 * | 0.9231/0.9 |
| cg19241468 | 3 | 122399344 | PARP14 |  | 0.0601/0.0467 * | 0.0486/0.0459 | 0.0573/0.0459 |
| cg01948202 | 3 | 122400474 | PARP14 |  | 0.2674/0.1452 ** | 0.1914/0.0915 ** | 0.2144/0.0826 ** |
| cg01721555 | 3 | 122401300 | PARP14 |  | 0.8606/0.7749 ** | 0.5517/0.3954 ** | 0.8956/0.8188 * |
| cg14750551 | 3 | 122401343 | PARP14 |  | 0.9079/0.7801 ** | 0.5038/0.3602 ** | 0.874/0.7343 * |
| cg18686270 | 3 | 146258875 | PLSCR1 |  | 0.8097/0.6877 ** | 0.6747/0.4638 ** | 0.9206/0.8479 |
| cg06981309 | 3 | 146260954 | PLSCR1 |  | 0.5292/0.2724 ** | 0.5524/0.2467 ** | 0.3738/0.1068 ** |
| cg20586531 | 3 | 146263147 | PLSCR1 |  | 0.8841/0.855 * | 0.8487/0.8386 | 0.8433/0.7902 |
| cg07809027 | 4 | 15007205 | CPEB2 |  | 0.6352/0.5309 ** | 0.4857/0.4048 * | 0.5535/0.4483 * |
| cg00598235 | 4 | 17580680 | LAP3 |  | 0.7477/0.6677 ** | 0.6998/0.6149 * | 0.8177/0.7286 * |
| cg08750951 | 4 | 89378894 | HERC5 |  | 0.0474/0.0236 * | 0.0585/0.0274 ** | 0.0216/0.0171 |
| cg02215171 | 4 | 89379156 | HERC5 |  | 0.4054/0.2926 ** | 0.4097/0.3049 ** | 0.2159/0.149 * |
| cg22300794 | 4 | 89427070 | HERC5 |  | 0.9074/0.9334 * | 0.914/0.9282 | 0.973/0.983 |
| cg04395703 | 4 | 99982762 | METAP1 |  | 0.6745/0.7142 * | 0.9345/0.9012 | 0.9562/0.9583 |
| cg05883128 | 4 | 169239131 | DDX60 |  | 0.2529/0.1368 ** | 0.4618/0.2283 ** | 0.4945/0.1943 ** |
| cg07016276 | 6 | 29692009 | HLA-F | IFN | 0.0127/0.01 | 0.0327/0.021 * | 0.0223/0.0141 |
| cg20927242 | 6 | 29692011 | HLA-F | IFN | 0.0381/0.0285 | 0.0778/0.0526 * | 0.0735/0.057 |
| cg09296453 | 6 | 29692035 | HLA-F | IFN | 0.1135/0.0935 | 0.2123/0.1359 ** | 0.1664/0.108 |
| cg12588917 | 6 | 29692082 | HLA-F | IFN | 0.3727/0.3472 | 0.5192/0.4449 ** | 0.5117/0.4246 * |
| cg23892836 | 6 | 29692085 | HLA-F | IFN | 0.3968/0.3798 | 0.5674/0.4859 ** | 0.6663/0.5684 * |
| cg24351901 | 6 | 29692092 | HLA-F | IFN | 0.1982/0.18 | 0.3447/0.289 ** | 0.3597/0.2866 * |
| cg15331332 | 6 | 29692111 | HLA-F | IFN | 0.3512/0.3406 | 0.5315/0.4507 * | 0.6839/0.5552 ** |
| cg00504902 | 6 | 29692183 | HLA-F | IFN | 0.5579/0.5255 * | 0.6003/0.565 | 0.6558/0.6135 |
| cg11617938 | 6 | 29692281 | HLA-F | IFN | 0.607/0.592 | 0.7683/0.7219 | 0.9131/0.8392 ** |
| cg11587584 | 6 | 29692372 | HLA-F | IFN | 0.594/0.6019 | 0.8081/0.7611 | 0.9284/0.8949 * |
| cg10601943 | 6 | 29692824 | HLA-F | IFN | 0.4717/0.4714 | 0.5931/0.5433 | 0.8551/0.7998 * |
| cg03154077 | 6 | 31323284 | HLA-B | IFN | 0.5409/0.5327 | 0.6587/0.6207 | 0.8069/0.7447 * |
| cg11187245 | 6 | 31323397 | HLA-B | IFN | 0.6108/0.6076 | 0.5587/0.5079 | 0.8534/0.7247 * |
| cg25954539 | 6 | 31323677 | HLA-B | IFN | 0.1325/0.1125 | 0.268/0.1867 ** | 0.319/0.1889 |
| cg17159161 | 6 | 31323760 | HLA-B | IFN | 0.0296/0.0258 | 0.0647/0.0454 ** | 0.0333/0.0251 |
| cg03500977 | 6 | 31325314 | HLA-B | IFN | 0.1507/0.1371 * | 0.1379/0.1217 | 0.1126/0.1048 |
| cg20408074 | 6 | 31325817 | HLA-B | IFN | 0.2371/0.2426 | 0.4281/0.3366 * | 0.4322/0.337 |
| cg25843003 | 6 | 31431312 | HCP5 |  | 0.0967/0.0785 * | 0.1706/0.1199 ** | 0.2105/0.106 * |
| cg00218406 | 6 | 31431407 | HCP5 |  | 0.2284/0.1932 * | 0.3433/0.2337 ** | 0.508/0.3009 * |
| cg18808777 | 6 | 31431503 | HCP5 |  | 0.2222/0.1935 | 0.389/0.2556 ** | 0.5182/0.3251 * |
| cg21684411 | 6 | 31431573 | HCP5 |  | 0.063/0.0525 * | 0.0461/0.0367 * | 0.0923/0.0475 * |
| cg23232773 | 6 | 31431902 | HCP5 |  | 0.3563/0.3591 | 0.5321/0.4619 | 0.8579/0.6984 * |
| cg01082299 | 6 | 31431969 | HCP5 |  | 0.2638/0.2871 | 0.4959/0.3881 * | 0.6973/0.539 * |
| cg14635654 | 6 | 32154447 | PBX2 |  | 0.6978/0.7089 | 0.686/0.7187 ** | 0.7572/0.7815 |
| cg18809947 | 6 | 32154454 | PBX2 |  | 0.7969/0.7988 | 0.7076/0.7394 * | 0.8982/0.9075 |
| cg00869530 | 6 | 32155124 | PBX2 |  | 0.5252/0.548 * | 0.5636/0.6036 * | 0.614/0.6618 * |
| cg04363228 | 6 | 32155141 | PBX2 |  | 0.4455/0.4854 * | 0.5128/0.5747 * | 0.5067/0.5668 |
| cg03280235 | 6 | 32158953 | PBX2; GPSM3 |  | 0.4089/0.4587 * | 0.595/0.5859 | 0.8137/0.8095 |
| cg16609995 | 6 | 32159036 | PBX2; GPSM3 |  | 0.2043/0.2585 * | 0.5389/0.5467 | 0.6397/0.64 |
| cg20397692 | 6 | 32802598 | TAP2 |  | 0.8706/0.8329 * | 0.9669/0.9603 | 0.9909/0.9872 |
| cg03438552 | 6 | 32805548 | TAP2 |  | 0.5031/0.459 * | 0.5532/0.4982 | 0.5473/0.4712 * |
| cg22940798 | 6 | 32805554 | TAP2 |  | 0.3409/0.2833 ** | 0.4533/0.3796 * | 0.3877/0.3207 * |
| cg08998192 | 6 | 32805570 | TAP2 |  | 0.3905/0.3323 ** | 0.4475/0.3838 * | 0.446/0.3662 * |
| cg13563634 | 6 | 32805684 | TAP2 |  | 0.5105/0.476 * | 0.4678/0.4153 | 0.614/0.5585 |
| cg26685246 | 6 | 32805692 | TAP2 |  | 0.3699/0.3243 * | 0.3833/0.3248 | 0.4379/0.3698 |
| cg23560159 | 6 | 32805748 | TAP2 |  | 0.1325/0.1139 * | 0.1047/0.0921 | 0.1105/0.0992 |
| cg10088320 | 6 | 32805759 | TAP2 |  | 0.1663/0.141 * | 0.1219/0.1029 * | 0.105/0.093 |
| cg22230640 | 6 | 32805807 | TAP2 |  | 0.0549/0.0465 * | 0.0448/0.0387 | 0.0435/0.0368 |
| cg03807983 | 6 | 32817662 | TAP1 |  | 0.9292/0.9086 * | 0.9788/0.9717 | 0.9279/0.9178 |
| cg10550816 | 6 | 32818452 | TAP1 |  | 0.6833/0.6325 * | 0.872/0.8361 | 0.7075/0.7164 |
| cg06473288 | 6 | 32820102 | TAP1 |  | 0.5558/0.5573 | 0.7092/0.6382 | 0.9852/0.95 * |
| cg10666909 | 6 | 32820249 | TAP1 |  | 0.4519/0.4688 | 0.6348/0.5507 | 0.8799/0.828 * |
| cg08818207 | 6 | 32820355 | TAP1 |  | 0.222/0.1974 | 0.4135/0.2874 ** | 0.5448/0.359 ** |
| cg04773990 | 6 | 32820410 | TAP1 |  | 0.0256/0.0238 | 0.0389/0.0304 * | 0.0308/0.0173 * |
| cg24154161 | 6 | 32820421 | TAP1 |  | 0.0233/0.021 | 0.049/0.0364 ** | 0.0449/0.0271 * |
| cg13403689 | 6 | 32820641 | TAP1; PSMB9 |  | 0.0642/0.0592 * | 0.0708/0.0599 ** | 0.0998/0.0768 ** |
| cg15993458 | 6 | 32820691 | TAP1; PSMB9 |  | 0.0214/0.0195 | 0.0335/0.027 * | 0.0241/0.0172 |
| cg02567488 | 6 | 32822565 | TAP1; PSMB9 |  | 0.0321/0.03 | 0.0453/0.0322 ** | 0.0403/0.0289 |
| cg07156249 | 6 | 32822911 | TAP1; PSMB9 |  | 0.2125/0.1576 * | 0.257/0.1488 * | 0.18/0.09 |
| cg03465320 | 6 | 32823078 | TAP1; PSMB9 |  | 0.4096/0.372 | 0.4371/0.353 * | 0.5725/0.4857 |
| cg16853860 | 6 | 32823116 | TAP1; PSMB9 |  | 0.5147/0.4477 * | 0.4371/0.301 * | 0.7183/0.6219 |
| cg20962292 | 6 | 32826254 | PSMB9 |  | 0.8724/0.8426 * | 0.5764/0.5482 | 0.7985/0.7634 |
| cg09322555 | 6 | 32826281 | PSMB9 |  | 0.6839/0.6025 * | 0.4751/0.4425 | 0.2915/0.2427 |
| cg14284211 | 6 | 35570224 | FKBP5 |  | 0.1691/0.1535 | 0.685/0.5879 * | 0.0305/0.0283 |
| cg14642437 | 6 | 35652521 | FKBP5 |  | 0.8606/0.8173 * | 0.938/0.9183 | 0.8045/0.7808 |
| cg03546163 | 6 | 35654363 | FKBP5 |  | 0.2869/0.2056 * | 0.6387/0.5218 ** | 0.429/0.2296 * |
| cg20813374 | 6 | 35657180 | FKBP5 |  | 0.4965/0.4441 ** | 0.3888/0.3386 | 0.4997/0.4298 * |
| cg00130530 | 6 | 35657202 | FKBP5 |  | 0.684/0.6467 * | 0.5527/0.5127 | 0.7349/0.6856 |
| cg03591753 | 6 | 35659141 | FKBP5 |  | 0.6104/0.5351 * | 0.799/0.7709 | 0.3834/0.3655 |
| cg23416081 | 6 | 35693573 | FKBP5 |  | 0.2671/0.1859 * | 0.3894/0.3685 | 0.0344/0.0321 |
| cg00052684 | 6 | 35694245 | FKBP5 |  | 0.2102/0.1589 ** | 0.8396/0.8002 | 0.0921/0.0842 |
| cg25114611 | 6 | 35696870 | LOC285847; FKBP5 |  | 0.3282/0.2713 ** | 0.2312/0.2156 | 0.1661/0.1392 |
| cg19226017 | 6 | 35697185 | LOC285847; FKBP5 |  | 0.7768/0.7301 * | 0.8595/0.8457 | 0.7259/0.6813 |
| cg03873220 | 7 | 139760375 | PARP12 |  | 0.712/0.6536 ** | 0.8201/0.763 | 0.9358/0.9311 |
| cg12013713 | 7 | 139760671 | PARP12 |  | 0.6877/0.5967 ** | 0.7709/0.7079 ** | 0.5938/0.5049 |
| cg05994974 | 7 | 139761087 | PARP12 |  | 0.5036/0.3371 ** | 0.4731/0.3463 ** | 0.2719/0.1315 ** |
| cg08961450 | 8 | 66699054 | PDE7A |  | 0.6196/0.6981 * | 0.8259/0.7981 | 0.9033/0.9087 |
| cg13375690 | 8 | 66706202 | PDE7A |  | 0.5404/0.5982 * | 0.7168/0.7202 | 0.832/0.837 |
| cg06222774 | 8 | 66743912 | PDE7A |  | 0.4174/0.5019 * | 0.6282/0.5811 | 0.8631/0.7944 |
| cg14864167 | 8 | 66751182 | PDE7A |  | 0.1869/0.1561 | 0.4903/0.287 ** | 0.5439/0.1758 ** |
| cg12110437 | 8 | 144098888 | LY6E; LOC100133669 |  | 0.3084/0.186 ** | 0.0699/0.0722 | 0.3642/0.1451 * |
| cg17052170 | 8 | 144099482 | LY6E; LOC100133669 |  | 0.6545/0.5483 ** | 0.6851/0.5476 ** | 0.5938/0.4439 |
| cg04678793 | 8 | 144099566 | LY6E; LOC100133669 |  | 0.0526/0.0411 ** | 0.0588/0.0429 ** | 0.042/0.0297 |
| cg12143168 | 8 | 144099627 | LY6E; LOC100133669 |  | 0.0379/0.033 | 0.0276/0.0181 * | 0.0207/0.0196 |
| cg11702942 | 8 | 144102584 | LY6E |  | 0.7791/0.6624 ** | 0.7861/0.6968 ** | 0.8008/0.6748 * |
| cg14392283 | 8 | 144103587 | LY6E |  | 0.8977/0.7314 ** | 0.842/0.6474 ** | 0.9613/0.9257 |
| cg03848588 | 9 | 32525008 | DDX58 | IFN | 0.8901/0.8235 ** | 0.9157/0.878 * | 0.9018/0.8446 * |
| cg14286514 | 9 | 32525315 | DDX58 | IFN | 0.6017/0.524 * | 0.6522/0.5973 | 0.6603/0.584 |
| cg11317199 | 9 | 100850391 | TRIM14 |  | 0.5968/0.6858 ** | 0.421/0.5732 ** | 0.588/0.7958 * |
| cg01765174 | 9 | 100880960 | TRIM14 |  | 0.4514/0.3903 ** | 0.6586/0.5836 | 0.5031/0.4179 * |
| cg01618660 | 9 | 100882376 | TRIM14 |  | 0.7639/0.707 * | 0.9349/0.914 * | 0.4639/0.4651 |
| cg27027427 | 10 | 91091912 | IFIT3 | IFN | 0.1204/0.1074 | 0.151/0.107 * | 0.1531/0.1118 |
| cg06188083 | 10 | 91093005 | IFIT3 | IFN | 0.3073/0.1714 ** | 0.2701/0.1289 ** | 0.4207/0.1225 ** |
| cg02370832 | 10 | 91093681 | IFIT3 | IFN | 0.383/0.2793 ** | 0.4521/0.2753 ** | 0.5359/0.2824 * |
| cg26974214 | 10 | 91151885 | IFIT1 | IFN | 0.1266/0.0867 ** | 0.1121/0.082 ** | 0.0716/0.058 |
| cg15019617 | 10 | 91152447 | IFIT1 | IFN | 0.0286/0.0232 * | 0.0296/0.0284 | 0.0279/0.0267 |
| cg05552874 | 10 | 91153143 | IFIT1 | IFN | 0.6583/0.3874 ** | 0.6731/0.3878 ** | 0.4025/0.1165 ** |
| cg06376949 | 10 | 91173811 | IFIT5 | IFN | 0.4149/0.2749 ** | 0.3683/0.2812 ** | 0.2159/0.138 * |
| cg01355957 | 10 | 91175165 | IFIT5 | IFN | 0.0331/0.0235 * | 0.0293/0.0231 | 0.0403/0.0294 |
| cg13172359 | 10 | 91175366 | IFIT5 | IFN | 0.3495/0.1842 ** | 0.1258/0.1151 | 0.2072/0.1331 |
| cg05432003 | 11 | 312518 | IFITM1 | IFN | 0.3569/0.2951 * | 0.7462/0.5894 ** | 0.7409/0.6467 |
| cg01886988 | 11 | 312560 | IFITM1 | IFN | 0.1474/0.126 * | 0.3957/0.2749 ** | 0.4019/0.3041 * |
| cg27032101 | 11 | 312841 | IFITM1 | IFN | 0.5242/0.4748 | 0.6928/0.5611 ** | 0.8045/0.7414 |
| cg04582010 | 11 | 313120 | IFITM1 | IFN | 0.2297/0.177 * | 0.6119/0.401 ** | 0.6339/0.4715 ** |
| cg09026253 | 11 | 313267 | IFITM1 | IFN | 0.3463/0.3069 | 0.7139/0.4917 ** | 0.8609/0.7286 * |
| cg11694510 | 11 | 313354 | IFITM1 | IFN | 0.4129/0.3809 * | 0.6241/0.4754 ** | 0.8033/0.6828 * |
| cg10552523 | 11 | 313478 | IFITM1 | IFN | 0.1729/0.1287 ** | 0.0605/0.0453 * | 0.525/0.3445 ** |
| cg20566897 | 11 | 313527 | IFITM1 | IFN | 0.2595/0.2081 * | 0.1497/0.0937 ** | 0.6031/0.439 ** |
| cg01971407 | 11 | 313624 | IFITM1 | IFN | 0.2923/0.2385 ** | 0.5257/0.3612 ** | 0.556/0.3857 ** |
| cg14967066 | 11 | 314341 | IFITM1 | IFN | 0.0572/0.0532 | 0.0698/0.0581 * | 0.0976/0.0753 * |
| cg08275025 | 11 | 314493 | IFITM1 | IFN | 0.0762/0.0667 * | 0.0935/0.0791 * | 0.125/0.0886 * |
| cg23570810 | 11 | 315102 | IFITM1 | IFN | 0.2711/0.1905 ** | 0.746/0.427 ** | 0.8223/0.4353 ** |
| cg21686213 | 11 | 315118 | IFITM1 | IFN | 0.2883/0.275 | 0.8208/0.5372 ** | 0.8475/0.6962 * |
| cg03038262 | 11 | 315262 | IFITM1 | IFN | 0.1896/0.1427 * | 0.6092/0.3158 ** | 0.7699/0.4012 ** |
| cg17990365 | 11 | 319718 | IFITM3 | IFN | 0.29/0.2396 * | 0.7326/0.5721 ** | 0.7003/0.4728 ** |
| cg24897127 | 11 | 320439 | IFITM3 | IFN | 0.0891/0.0725 * | 0.0786/0.0683 | 0.0473/0.0427 |
| cg15218509 | 11 | 320836 | IFITM3 | IFN | 0.3887/0.3104 * | 0.2172/0.1972 | 0.025/0.0205 |
| cg06046490 | 11 | 320940 | IFITM3 | IFN | 0.2215/0.1656 * | 0.0819/0.0747 | 0.028/0.0254 |
| cg20691577 | 11 | 577203 | PHRF1; LOC143666 |  | 0.0286/0.0252 * | 0.0319/0.0333 | 0.035/0.0332 |
| cg16016431 | 11 | 611390 | PHRF1 |  | 0.8525/0.8214 * | 0.8647/0.8201 | 0.9049/0.8601 * |
| cg19974879 | 11 | 611692 | PHRF1 |  | 0.9344/0.912 ** | 0.9245/0.8959 * | 0.9354/0.9204 |
| cg11791770 | 11 | 611791 | PHRF1 |  | 0.8763/0.8402 ** | 0.8954/0.8597 * | 0.871/0.8292 |
| cg03755158 | 11 | 612680 | IRF7 | IFN | 0.9044/0.8793 * | 0.9388/0.9269 | 0.9097/0.8646 |
| cg27271532 | 11 | 612762 | IRF7 | IFN | 0.7845/0.7507 * | 0.8497/0.8345 | 0.761/0.7205 |
| cg05309505 | 11 | 612837 | IRF7 | IFN | 0.9657/0.9514 ** | 0.9714/0.9522 * | 0.9555/0.9343 * |
| cg20989454 | 11 | 613478 | IRF7 | IFN | 0.8068/0.7921 | 0.8445/0.8171 | 0.8758/0.8202 * |
| cg16486109 | 11 | 613632 | IRF7 | IFN | 0.5306/0.4942 | 0.6791/0.6242 | 0.8334/0.7126 * |
| cg17114584 | 11 | 613792 | IRF7 | IFN | 0.1994/0.1801 | 0.5456/0.4834 | 0.5521/0.3536 * |
| cg08926253 | 11 | 614761 | IRF7 | IFN | 0.5807/0.4193 ** | 0.6811/0.5414 ** | 0.3958/0.1816 ** |
| cg22016995 | 11 | 614787 | IRF7 | IFN | 0.9371/0.8352 ** | 0.9321/0.8753 * | 0.8823/0.7616 |
| cg13406746 | 11 | 615583 | IRF7 | IFN | 0.1073/0.0946 * | 0.1132/0.1012 | 0.1576/0.1304 |
| cg12461141 | 11 | 5710654 | TRIM22 |  | 0.4515/0.3471 ** | 0.4143/0.3583 * | 0.2706/0.2007 |
| cg26724018 | 11 | 5716255 | TRIM22 |  | 0.2138/0.1543 ** | 0.4303/0.3187 ** | 0.2747/0.198 * |
| cg09151598 | 12 | 113343409 | OAS1 | IFN | 0.7577/0.7097 * | 0.6586/0.5785 * | 0.6878/0.6812 |
| cg17445535 | 12 | 113343413 | OAS1 | IFN | 0.8095/0.7647 * | 0.7264/0.6832 | 0.8164/0.7924 |
| cg25668626 | 12 | 113343603 | OAS1 | IFN | 0.878/0.8077 ** | 0.7992/0.7409 | 0.9048/0.8741 |
| cg19789466 | 12 | 113344923 | OAS1 | IFN | 0.0774/0.0537 ** | 0.0635/0.0498 ** | 0.0632/0.0468 ** |
| cg04951822 | 12 | 113345598 | OAS1 | IFN | 0.247/0.1939 ** | 0.2657/0.2306 | 0.2295/0.2126 |
| cg22260958 | 12 | 113375880 | OAS3 | IFN | 0.7689/0.6989 ** | 0.747/0.6674 * | 0.7569/0.6853 |
| cg25800166 | 12 | 113375896 | OAS3 | IFN | 0.5795/0.4634 ** | 0.6453/0.5697 * | 0.5506/0.4301 * |
| cg23893332 | 14 | 94576048 | IFI27 | IFN | 0.6843/0.6154 * | 0.7536/0.7646 | 0.4379/0.3756 |
| cg03447547 | 14 | 94577039 | IFI27 | IFN | 0.8253/0.7465 ** | 0.8252/0.7964 | 0.8454/0.7861 |
| cg10778971 | 14 | 94577101 | IFI27 | IFN | 0.8444/0.7088 ** | 0.8622/0.797 * | 0.8501/0.7154 * |
| cg08036899 | 14 | 94577218 | IFI27 | IFN | 0.898/0.8651 ** | 0.9007/0.8724 * | 0.9274/0.9106 |
| cg20161089 | 14 | 94577514 | IFI27 | IFN | 0.8862/0.8549 * | 0.9085/0.888 | 0.9326/0.923 |
| cg02013741 | 15 | 67006698 | SMAD6 |  | 0.5047/0.4548 * | 0.0423/0.0501 | 0.0605/0.0596 |
| cg17232357 | 15 | 67012832 | SMAD6 |  | 0.4627/0.3879 ** | 0.5216/0.4588 * | 0.4515/0.3566 * |
| cg16889659 | 15 | 67043450 | SMAD6 |  | 0.7558/0.8073 * | 0.9229/0.9196 | 0.9755/0.9708 |
| cg18778196 | 15 | 67054877 | SMAD6 |  | 0.2801/0.2413 * | 0.1004/0.1038 | 0.0934/0.0965 |
| cg10577241 | 15 | 77456283 | SGK269 |  | 0.3698/0.4615 ** | 0.9074/0.8888 | 0.9364/0.943 |
| cg23387863 | 15 | 77472416 | SGK269 |  | 0.7388/0.6857 ** | 0.8249/0.758 ** | 0.8079/0.7268 * |
| cg08130265 | 15 | 77519170 | SGK269; C15orf5 |  | 0.6641/0.6033 * | 0.825/0.8074 | 0.2697/0.2785 |
| cg08159663 | 16 | 57022486 | NLRC5 | IFN | 0.4839/0.4237 * | 0.6975/0.5879 * | 0.713/0.585 * |
| cg07839457 | 16 | 57023022 | NLRC5 | IFN | 0.2919/0.1809 ** | 0.579/0.3292 ** | 0.5975/0.218 ** |
| cg16411857 | 16 | 57023191 | NLRC5 | IFN | 0.1092/0.0839 ** | 0.2583/0.1456 ** | 0.2893/0.1376 ** |
| cg02452732 | 17 | 41158611 | IFI35 | IFN | 0.4568/0.4216 * | 0.4401/0.4273 | 0.5461/0.5023 |
| cg08090640 | 17 | 41159289 | IFI35 | IFN | 0.4713/0.4195 * | 0.5977/0.5149 ** | 0.6038/0.5309 * |
| cg06720017 | 17 | 76967629 | LGALS3BP |  | 0.136/0.1296 | 0.1211/0.1478 * | 0.0445/0.0476 |
| cg18749404 | 17 | 76975944 | LGALS3BP |  | 0.6511/0.6115 | 0.8444/0.7875 * | 0.8877/0.7965 * |
| cg14870271 | 17 | 76976010 | LGALS3BP |  | 0.1754/0.1533 * | 0.1985/0.1796 | 0.3067/0.201 * |
| cg04927537 | 17 | 76976091 | LGALS3BP |  | 0.2252/0.1713 * | 0.2948/0.2275 * | 0.4618/0.2922 * |
| cg22713958 | 17 | 76976245 | LGALS3BP |  | 0.5817/0.5511 | 0.6897/0.6208 ** | 0.8258/0.7425 * |
| cg25178683 | 17 | 76976267 | LGALS3BP |  | 0.372/0.3264 * | 0.5005/0.3992 ** | 0.6036/0.454 * |
| cg11105610 | 17 | 76976352 | LGALS3BP |  | 0.6185/0.6217 | 0.7341/0.6185 ** | 0.9299/0.9029 |
| cg17836612 | 17 | 76976357 | LGALS3BP |  | 0.4759/0.4743 | 0.6446/0.5576 ** | 0.8262/0.7309 * |
| cg11622162 | 17 | 78237543 | RNF213 |  | 0.0945/0.0831 * | 0.1266/0.0992 ** | 0.1612/0.1197 ** |
| cg06603074 | 18 | 60192893 | ZCCHC2 |  | 0.3009/0.258 * | 0.3702/0.2843 * | 0.5035/0.3734 ** |
| cg22642495 | 19 | 10197856 | C19orf66 |  | 0.0914/0.0673 ** | 0.1931/0.1304 ** | 0.1677/0.1061 * |
| cg13419792 | 19 | 10197996 | C19orf66 |  | 0.8954/0.8513 ** | 0.8411/0.8076 | 0.8809/0.8447 * |
| cg22282590 | 19 | 17514117 | BST2 | IFN | 0.9164/0.8846 * | 0.9323/0.917 | 0.9102/0.861 * |
| cg07839313 | 19 | 17514600 | BST2 | IFN | 0.5056/0.4346 ** | 0.516/0.4425 ** | 0.3488/0.2648 |
| cg12090003 | 19 | 17516282 | BST2 | IFN | 0.0644/0.0416 ** | 0.0247/0.0249 | 0.0246/0.0217 |
| cg16363586 | 19 | 17516329 | BST2 | IFN | 0.2421/0.1624 ** | 0.0894/0.071 ** | 0.0561/0.0428 |
| cg11558551 | 19 | 17516442 | BST2 | IFN | 0.0419/0.0297 ** | 0.0202/0.0213 | 0.0187/0.0171 |
| cg01254505 | 19 | 17516470 | BST2 | IFN | 0.0587/0.0405 ** | 0.0259/0.0255 | 0.0258/0.0241 |
| cg01329005 | 19 | 17516712 | BST2 | IFN | 0.1312/0.0785 ** | 0.0459/0.0399 * | 0.0361/0.0277 |
| cg09993699 | 19 | 17517008 | BST2 | IFN | 0.1359/0.0876 ** | 0.0707/0.0561 * | 0.043/0.0364 |
| cg20092122 | 19 | 17517221 | BST2 | IFN | 0.0975/0.0569 ** | 0.033/0.0308 | 0.0308/0.0269 |
| cg15262954 | 20 | 62198872 | PRIC285 |  | 0.123/0.1758 * | 0.0308/0.0666 ** | 0.0241/0.029 |
| cg17593958 | 20 | 62199034 | PRIC285 |  | 0.1783/0.2245 * | 0.1309/0.1442 | 0.1129/0.1184 |
| cg11779113 | 20 | 62199156 | PRIC285 |  | 0.0622/0.0881 * | 0.0311/0.0336 | 0.0271/0.0263 |
| cg06064964 | 20 | 62199181 | PRIC285 |  | 0.0883/0.1376 ** | 0.0326/0.0369 | 0.0321/0.0324 |
| cg09844573 | 20 | 62199190 | PRIC285 |  | 0.1216/0.173 ** | 0.046/0.0509 | 0.0443/0.0445 |
| cg17864091 | 20 | 62200091 | PRIC285 |  | 0.4908/0.526 * | 0.4259/0.4688 | 0.4578/0.4663 |
| cg04300115 | 20 | 62200199 | PRIC285 |  | 0.5951/0.6458 * | 0.5681/0.6211 | 0.7564/0.8161 |
| cg06474274 | 20 | 62204482 | PRIC285 |  | 0.8545/0.828 * | 0.801/0.7886 | 0.8685/0.8411 |
| cg01190666 | 20 | 62204908 | PRIC285 |  | 0.6041/0.456 ** | 0.4994/0.3762 ** | 0.4727/0.3089 ** |
| cg07649744 | 20 | 62205876 | PRIC285 |  | 0.0551/0.0499 * | 0.0443/0.0434 | 0.0425/0.0431 |
| cg15402619 | 20 | 62205886 | PRIC285 |  | 0.068/0.0603 * | 0.0565/0.0521 | 0.0487/0.0479 |
| cg06295590 | 20 | 62205981 | PRIC285 |  | 0.2001/0.1653 ** | 0.1329/0.1204 | 0.1239/0.1019 |
| cg16785077 | 21 | 42791867 | MX1 | IFN | 0.7702/0.6702 ** | 0.8529/0.8062 * | 0.5967/0.4892 |
| cg16733866 | 21 | 42792609 | MX1 | IFN | 0.1632/0.1367 * | 0.1636/0.1525 | 0.1212/0.1194 |
| cg13507964 | 21 | 42792703 | MX1 | IFN | 0.0482/0.0341 ** | 0.0324/0.0299 | 0.0325/0.0301 |
| cg22862003 | 21 | 42797588 | MX1 | IFN | 0.6159/0.3444 ** | 0.5932/0.3248 ** | 0.5506/0.171 ** |
| cg26312951 | 21 | 42797847 | MX1 | IFN | 0.2116/0.1138 ** | 0.2438/0.1187 ** | 0.2632/0.048 ** |
| cg12359279 | 21 | 42797953 | MX1 | IFN | 0.0232/0.0194 * | 0.0276/0.0243 | 0.0352/0.0246 ** |
| cg08924203 | 21 | 42798747 | MX1 | IFN | 0.1026/0.0534 ** | 0.1071/0.0631 ** | 0.1125/0.0606 * |
| cg21549285 | 21 | 42799141 | MX1 | IFN | 0.5598/0.21 ** | 0.6305/0.2417 ** | 0.6554/0.0764 ** |
| cg16751754 | 21 | 42801063 | MX1 | IFN | 0.9345/0.9127 * | 0.927/0.9128 | 0.9949/0.9884 |
| cg27281093 | 22 | 18632404 | USP18 | IFN | 0.1964/0.162 * | 0.228/0.1988 | 0.2926/0.2493 |
| cg18699242 | 22 | 18632429 | USP18 | IFN | 0.4031/0.3367 * | 0.405/0.3566 | 0.44/0.3932 |
| cg01963623 | 22 | 18632433 | USP18 | IFN | 0.3932/0.3356 * | 0.3978/0.3525 | 0.4098/0.3741 |
| cg10685559 | 22 | 18633123 | USP18 | IFN | 0.0427/0.0322 ** | 0.0458/0.0315 ** | 0.044/0.0332 |
| cg14293575 | 22 | 18635460 | USP18 | IFN | 0.4823/0.2401 ** | 0.582/0.251 ** | 0.7075/0.3913 * |
| cg21881327 | 22 | 24979569 | GGT1 |  | 0.8697/0.8528 * | 0.835/0.8232 | 0.9126/0.8925 |
| cg05543864 | 22 | 24979755 | GGT1 |  | 0.7086/0.6645 ** | 0.7268/0.6871 ** | 0.7007/0.6302 * |
| cg22764925 | 22 | 24979964 | GGT1 |  | 0.67/0.5708 ** | 0.6849/0.5859 ** | 0.685/0.5048 ** |
| cg22123459 | 22 | 25003499 | GGT1 |  | 0.288/0.2583 * | 0.3382/0.3288 | 0.1505/0.1597 |
| cg05577434 | 22 | 25003527 | GGT1 |  | 0.1893/0.1642 * | 0.2103/0.1993 | 0.121/0.1202 |
| cg23363526 | 22 | 25003552 | GGT1 |  | 0.3369/0.3057 * | 0.4324/0.4171 | 0.2469/0.2468 |
| cg24713529 | 22 | 50965126 | TYMP; SCO2 |  | 0.0264/0.0208 * | 0.0271/0.0247 | 0.0191/0.0181 |
| cg13092901 | 22 | 50965373 | TYMP; SCO2 |  | 0.2021/0.1348 ** | 0.1139/0.0798 ** | 0.0554/0.0534 |
| cg02776313 | 22 | 50965782 | TYMP; SCO2 |  | 0.7202/0.6767 * | 0.7173/0.7015 | 0.7505/0.674 * |
| cg10416593 | 22 | 50966123 | TYMP; SCO2 |  | 0.6716/0.5673 ** | 0.7665/0.7311 | 0.2827/0.2594 |
| cg06345736 | 22 | 50966936 | TYMP |  | 0.8601/0.8399 * | 0.8848/0.8651 * | 0.9136/0.8925 |
| cg19354746 | 22 | 50969673 | ODF3B; TYMP |  | 0.2036/0.1911 | 0.2063/0.189 * | 0.166/0.1627 |
| cg16367976 | 22 | 50969678 | ODF3B; TYMP |  | 0.0512/0.0364 * | 0.0572/0.0349 * | 0.0242/0.0182 |
| cg23221113 | 22 | 50970943 | ODF3B |  | 0.1115/0.0632 ** | 0.0482/0.0348 ** | 0.0266/0.0247 |
| cg11224765 | 22 | 50971109 | ODF3B |  | 0.3229/0.2043 ** | 0.2053/0.1349 ** | 0.0943/0.0665 |
| cg20098015 | 22 | 50971140 | ODF3B |  | 0.4261/0.2891 ** | 0.4295/0.2755 ** | 0.2475/0.1079 * |
| cg16644494 | 22 | 50971601 | ODF3B |  | 0.8037/0.7344 ** | 0.8652/0.8304 | 0.6351/0.4929 * |
| cg22910549 | 22 | 50985117 | KLHDC7B |  | 0.84/0.7948 * | 0.8351/0.8153 | 0.7644/0.6998 |
| cg07833467 | 22 | 50986511 | KLHDC7B |  | 0.4816/0.3563 ** | 0.244/0.1873 | 0.4588/0.2998 |
| cg18533225 | 22 | 50986813 | KLHDC7B |  | 0.4315/0.3551 ** | 0.3658/0.2986 * | 0.442/0.3009 * |
| cg11829870 | 22 | 50988451 | KLHDC7B |  | 0.7418/0.6513 ** | 0.8194/0.791 | 0.6448/0.5402 |

Table of CpGs near genes with at least one CpG at 1 x 10^-8^ in one cell type and at least one CpG at FDR<1% in at least one other cell-type. The columns for each cell type indicate mean methylation proportion after correction for all covariates in controls/SLE patients. Highly significant effects (p < 1 x 10^-8^) have double asterisks. Moderately significant (FDR<1%) have single asterisks.
