# Supplementary material for: Genome-Wide DNA Methylation Analysis of Systemic Lupus Erythematosus Reveals Persistent Hypomethylation of Interferon Genes and Compositional Changes to CD4+ T-cell Populations
Source: PLoS Genet. 2013 Aug 8;9(8):e1003678. doi: 10.1371/journal.pgen.1003678 (PMC3738443; doi:10.1371/journal.pgen.1003678)
Supplement: Table S5 — List of cell type-specific genes in T-cells and B-cells. Listed are those genes with a highly significant (p<1×10^−8^) SLE-associated methylation difference that is unique to one cell-type. (DOCX) [file pgen.1003678.s009.docx]

**Table S5. List of Cell Type-Specific Genes in T-cells and B-cells.**

| **CD19 Unique Genes** |  |  |  |  |  |
| --- | --- | --- | --- | --- | --- |
| **CpG** | **Chr** | **Pos** | **Genes** | **IFN** | **CD19** |
| cg12393275 | 2 | 79312224 | REG1B |  | 0.4149/0.5503 |
| cg11714752 | 2 | 96926568 | TMEM127 |  | 0.085/0.1075 |
| cg13468041 | 4 | 74902951 | CXCL3 |  | 0.087/0.1178 |
| cg14283140 | 9 | 135763667 | C9orf9 |  | 0.0896/0.1258 |
| cg10462187 | 10 | 124067402 | BTBD16 |  | 0.0714/0.1162 |
| cg09104284 | 16 | 90050102 | AFG3L1 |  | 0.856/0.7826 |
| cg25739715 | 22 | 30663881 | OSM |  | 0.2258/0.1802 |
|  |  |  |  |  |  |
|  |  |  |  |  |  |
| **CD4 Unique Genes** |  |  |  |  |  |
| **CpG** | **Chr** | **Pos** | **Genes** | **IFN** | **CD4** |
| cg08840010 | 1 | 8000314 | TNFRSF9 |  | 0.6102/0.5177 |
| cg20305595 | 1 | 9293833 | H6PD |  | 0.5452/0.4917 |
| cg06829969 | 1 | 10460205 | PGD |  | 0.7758/0.7129 |
| cg21826784 | 1 | 11795937 | AGTRAP |  | 0.5183/0.4536 |
| cg25467652 | 1 | 11795976 | AGTRAP |  | 0.3304/0.2768 |
| cg03593358 | 1 | 12039985 | MFN2 |  | 0.1683/0.1297 |
| cg11217193 | 1 | 12538341 | VPS13D |  | 0.6152/0.6727 |
| cg19620994 | 1 | 12774904 | AADACL3 |  | 0.7426/0.6705 |
| cg09119494 | 1 | 23851471 | E2F2 |  | 0.063/0.0522 |
| cg05649922 | 1 | 27029290 | ARID1A |  | 0.1317/0.1658 |
| cg17820878 | 1 | 27440463 | SLC9A1 |  | 0.6882/0.7546 |
| cg13300580 | 1 | 27440539 | SLC9A1 |  | 0.2927/0.3444 |
| cg25130381 | 1 | 27440721 | SLC9A1 |  | 0.4617/0.521 |
| cg09725874 | 1 | 27480106 | SLC9A1 |  | 0.4995/0.5556 |
| cg03018771 | 1 | 27940619 | FGR |  | 0.3872/0.4932 |
| cg15482893 | 1 | 32837667 | BSDC1 |  | 0.1606/0.1995 |
| cg17971578 | 1 | 36852463 | STK40 |  | 0.3536/0.2909 |
| cg04430911 | 1 | 36914349 | OSCP1 |  | 0.6971/0.6147 |
| cg14285050 | 1 | 40778294 | COL9A2 |  | 0.2166/0.1763 |
| cg01763916 | 1 | 40849202 | SMAP2 |  | 0.6392/0.5396 |
| cg12666727 | 1 | 42128487 | HIVEP3 |  | 0.2869/0.2179 |
| cg24254842 | 1 | 42193353 | HIVEP3 |  | 0.2625/0.2007 |
| cg12590902 | 1 | 44771985 | ERI3 |  | 0.2366/0.2897 |
| cg11448683 | 1 | 45274099 | TCTEX1D4; BTBD19 |  | 0.2317/0.182 |
| cg06619077 | 1 | 47656003 | PDZK1IP1 |  | 0.6848/0.6271 |
| cg11399254 | 1 | 47694517 | TAL1 |  | 0.6333/0.5683 |
| cg24741609 | 1 | 54122060 | GLIS1 |  | 0.1578/0.1918 |
| cg19379103 | 1 | 54751576 | SSBP3 |  | 0.6459/0.7012 |
| cg17142950 | 1 | 84766782 | SAMD13 |  | 0.3986/0.3406 |
| cg01890417 | 1 | 91488275 | ZNF644 |  | 0.545/0.6604 |
| cg03260624 | 1 | 91970722 | CDC7 |  | 0.8219/0.7626 |
| cg07243548 | 1 | 108231160 | VAV3 |  | 0.6977/0.6257 |
| cg03725309 | 1 | 109757585 | SARS |  | 0.3126/0.2678 |
| cg09516523 | 1 | 111769133 | CHI3L2 |  | 0.4586/0.5505 |
| cg02590572 | 1 | 111769481 | CHI3L2 |  | 0.4887/0.5732 |
| cg14414943 | 1 | 111770718 | CHI3L2 |  | 0.5942/0.6736 |
| cg08529825 | 1 | 114489550 | HIPK1 |  | 0.3969/0.4667 |
| cg00356916 | 1 | 116256618 | CASQ2 |  | 0.6097/0.7002 |
| cg11120551 | 1 | 146713996 | CHD1L |  | 0.6226/0.5469 |
| cg21262032 | 1 | 154437693 | IL6R |  | 0.3262/0.3976 |
| cg26189283 | 1 | 155109378 | RAG1AP1 |  | 0.365/0.2998 |
| cg21877565 | 1 | 156461550 | MEF2D |  | 0.4227/0.3315 |
| cg16374333 | 1 | 157103641 | ETV3 |  | 0.3366/0.4614 |
| cg25597580 | 1 | 157964529 | KIRREL |  | 0.5782/0.6596 |
| cg11525252 | 1 | 158041312 | KIRREL |  | 0.51/0.6185 |
| cg15593510 | 1 | 158369112 | OR10T2 |  | 0.4303/0.5255 |
| cg18865207 | 1 | 160765919 | LY9 |  | 0.0777/0.103 |
| cg05138397 | 1 | 160768549 | LY9 |  | 0.5904/0.6583 |
| cg07106927 | 1 | 160769081 | LY9 |  | 0.6069/0.6957 |
| cg17013990 | 1 | 161091682 | DEDD |  | 0.5427/0.4403 |
| cg00160981 | 1 | 161691911 | FCRLB |  | 0.2642/0.2088 |
| cg09554443 | 1 | 167487762 | CD247 |  | 0.1525/0.2179 |
| cg03471346 | 1 | 179112077 | ABL2 |  | 0.418/0.5256 |
| cg08272268 | 1 | 200380059 | ZNF281 |  | 0.6861/0.6261 |
| cg01798157 | 1 | 203276595 | BTG2 |  | 0.0957/0.1461 |
| cg20867633 | 1 | 204183116 | GOLT1A |  | 0.0757/0.0591 |
| cg19638572 | 1 | 206733139 | RASSF5 |  | 0.1426/0.1999 |
| cg18234296 | 1 | 210407896 | C1orf133; SERTAD4 |  | 0.4884/0.558 |
| cg07107916 | 1 | 212457424 | PPP2R5A |  | 0.3287/0.4309 |
| cg15009294 | 1 | 214813712 | CENPF |  | 0.4602/0.5825 |
| cg17855595 | 1 | 249148077 | ZNF692 |  | 0.3878/0.4585 |
| cg04618171 | 2 | 10470465 | HPCAL1 |  | 0.6084/0.7129 |
| cg00141688 | 2 | 10517352 | HPCAL1 |  | 0.7416/0.8102 |
| cg06200244 | 2 | 12862337 | TRIB2 |  | 0.3261/0.4151 |
| cg08569786 | 2 | 23616768 | KLHL29 |  | 0.3337/0.4069 |
| cg00050692 | 2 | 25524877 | DNMT3A |  | 0.3941/0.3212 |
| cg08364093 | 2 | 28114164 | LOC100302650; RBKS; BRE |  | 0.1706/0.139 |
| cg15078838 | 2 | 28114349 | LOC100302650; RBKS; BRE |  | 0.374/0.2928 |
| cg00841141 | 2 | 37416819 | SULT6B1 |  | 0.7971/0.7412 |
| cg15657390 | 2 | 38155843 | FAM82A1 |  | 0.4945/0.5647 |
| cg09999348 | 2 | 46398848 | PRKCE |  | 0.6064/0.6824 |
| cg00883689 | 2 | 54802904 | SPTBN1 |  | 0.4521/0.3735 |
| cg17253709 | 2 | 62442007 | B3GNT2 |  | 0.4862/0.412 |
| cg25521400 | 2 | 62445279 | B3GNT2 |  | 0.6011/0.4848 |
| cg02956499 | 2 | 74280825 | TET3 |  | 0.5393/0.6198 |
| cg17512187 | 2 | 85082845 | C2orf89 |  | 0.237/0.2914 |
| cg16469046 | 2 | 99062999 | INPP4A |  | 0.3156/0.4406 |
| cg11555067 | 2 | 99081350 | INPP4A |  | 0.2715/0.336 |
| cg20923498 | 2 | 99096920 | INPP4A |  | 0.3973/0.4767 |
| cg25015733 | 2 | 99342986 | MGAT4A |  | 0.6152/0.7161 |
| cg23527387 | 2 | 100056660 | REV1 |  | 0.8719/0.8164 |
| cg10523140 | 2 | 101768941 | TBC1D8 |  | 0.2856/0.244 |
| cg01124420 | 2 | 109605518 | EDAR |  | 0.2365/0.2998 |
| cg02543462 | 2 | 113885116 | IL1RN |  | 0.3683/0.2899 |
| cg03989987 | 2 | 113885277 | IL1RN |  | 0.4059/0.3211 |
| cg25265126 | 2 | 113891507 | IL1RN |  | 0.5451/0.5971 |
| cg16682903 | 2 | 158694670 | ACVR1 |  | 0.7088/0.6309 |
| cg19412109 | 2 | 220082807 | ABCB6 |  | 0.2384/0.2042 |
| cg10835083 | 2 | 222395101 | EPHA4 |  | 0.3715/0.4741 |
| cg02225786 | 2 | 224808252 | WDFY1 |  | 0.8382/0.7958 |
| cg17607231 | 2 | 231090329 | SP140 |  | 0.1736/0.1229 |
| cg23539753 | 2 | 231280698 | SP100 | IFN | 0.0601/0.0506 |
| cg06096336 | 2 | 231989800 | PSMD1; HTR2B |  | 0.243/0.3387 |
| cg16967583 | 2 | 241807859 | AGXT |  | 0.5402/0.4624 |
| cg27217350 | 2 | 242813634 | C2orf85 |  | 0.447/0.5122 |
| cg26269881 | 3 | 5023310 | BHLHE40 |  | 0.4711/0.3626 |
| cg20916523 | 3 | 10184584 | VHL |  | 0.3168/0.4208 |
| cg21387009 | 3 | 10280255 | IRAK2 |  | 0.3479/0.4423 |
| cg21902966 | 3 | 18391029 | SATB1 |  | 0.6174/0.6996 |
| cg01373248 | 3 | 18480297 | SATB1 |  | 0.1423/0.2013 |
| cg04916416 | 3 | 30651317 | TGFBR2 |  | 0.4741/0.593 |
| cg19615017 | 3 | 30673459 | TGFBR2 |  | 0.3172/0.4077 |
| cg15171154 | 3 | 30722557 | TGFBR2 |  | 0.6338/0.7253 |
| cg17980786 | 3 | 32933637 | TRIM71 |  | 0.3731/0.4377 |
| cg25780219 | 3 | 35785041 | ARPP-21; MIR128-2 |  | 0.7307/0.7839 |
| cg21113478 | 3 | 36782467 | DCLK3 |  | 0.2803/0.3568 |
| cg00252934 | 3 | 36782736 | DCLK3 |  | 0.3885/0.4733 |
| cg23654821 | 3 | 39188656 | CSRNP1 |  | 0.1013/0.082 |
| cg03341377 | 3 | 39309355 | CX3CR1 |  | 0.3136/0.3935 |
| cg05461503 | 3 | 45837170 | SLC6A20 |  | 0.3839/0.4688 |
| cg24940967 | 3 | 45837197 | SLC6A20 |  | 0.253/0.3373 |
| cg20496896 | 3 | 46579532 | LRRC2 |  | 0.3832/0.4679 |
| cg04625862 | 3 | 48341880 | NME6 |  | 0.1149/0.1592 |
| cg14494596 | 3 | 48542040 | SHISA5 |  | 0.2902/0.2426 |
| cg21800196 | 3 | 48673931 | SLC26A6; CELSR3 |  | 0.6791/0.6127 |
| cg19381811 | 3 | 49851713 | UBA7 | IFN | 0.3938/0.3297 |
| cg01932734 | 3 | 50376409 | RASSF1 |  | 0.2055/0.1605 |
| cg05548488 | 3 | 50658472 | MAPKAPK3 |  | 0.1035/0.0832 |
| cg07615364 | 3 | 52088726 | DUSP7 |  | 0.2028/0.2576 |
| cg25236791 | 3 | 52302252 | WDR82 |  | 0.4232/0.5148 |
| cg25799109 | 3 | 57102900 | ARHGEF3; SPATA12 |  | 0.6429/0.5594 |
| cg02923224 | 3 | 60133085 | FHIT |  | 0.3959/0.5231 |
| cg08992499 | 3 | 63956514 | ATXN7 |  | 0.548/0.6516 |
| cg24969820 | 3 | 98497864 | ST3GAL6 |  | 0.3586/0.315 |
| cg03416645 | 3 | 105087206 | ALCAM |  | 0.0834/0.0625 |
| cg02487233 | 3 | 107810687 | CD47 |  | 0.5888/0.6785 |
| cg00492070 | 3 | 107810716 | CD47 |  | 0.5981/0.6977 |
| cg19400179 | 3 | 108321607 | DZIP3 |  | 0.1413/0.2203 |
| cg14535274 | 3 | 121151186 | POLQ |  | 0.0655/0.088 |
| cg07578772 | 3 | 150420821 | FAM194A |  | 0.3231/0.4326 |
| cg13702222 | 3 | 152017240 | MBNL1 |  | 0.2151/0.3045 |
| cg25857569 | 3 | 160717237 | PPM1L |  | 0.5832/0.6787 |
| cg14612335 | 3 | 170074131 | SKIL |  | 0.1992/0.1415 |
| cg07266910 | 3 | 178745575 | ZMAT3 |  | 0.3483/0.4322 |
| cg04400533 | 3 | 185788716 | ETV5 |  | 0.4691/0.5438 |
| cg02035018 | 4 | 2299988 | ZFYVE28 |  | 0.6005/0.6734 |
| cg17276535 | 4 | 3372123 | RGS12 |  | 0.2151/0.2687 |
| cg03132824 | 4 | 3373006 | RGS12 |  | 0.5019/0.587 |
| cg06970472 | 4 | 40910981 | APBB2 |  | 0.2954/0.2357 |
| cg26542660 | 4 | 56813860 | CEP135 |  | 0.1774/0.1326 |
| cg21446955 | 4 | 86851425 | ARHGAP24 |  | 0.0778/0.0989 |
| cg08979352 | 4 | 99580530 | TSPAN5 |  | 0.6449/0.5384 |
| cg25663524 | 4 | 103489295 | NFKB1 |  | 0.7785/0.8414 |
| cg14459011 | 4 | 103998497 | NHEDC2 |  | 0.5604/0.4852 |
| cg01150799 | 4 | 108955838 | HADH |  | 0.2795/0.3465 |
| cg24124145 | 4 | 139936112 | CCRN4L |  | 0.4856/0.4374 |
| cg02830749 | 4 | 154451068 | KIAA0922 |  | 0.5577/0.4455 |
| cg00675600 | 5 | 1255458 | TERT |  | 0.4567/0.5431 |
| cg13390570 | 5 | 1255616 | TERT |  | 0.3712/0.4917 |
| cg24894783 | 5 | 10577031 | ANKRD33B |  | 0.7187/0.649 |
| cg22953759 | 5 | 10632397 | ANKRD33B |  | 0.3134/0.2353 |
| cg15529432 | 5 | 16615750 | FAM134B |  | 0.5958/0.6897 |
| cg00461022 | 5 | 16618052 | FAM134B |  | 0.3219/0.4006 |
| cg09452568 | 5 | 54275198 | ESM1 |  | 0.7722/0.6895 |
| cg01096487 | 5 | 55184235 | IL31RA |  | 0.4687/0.5709 |
| cg11497377 | 5 | 65465543 | SFRS12 |  | 0.3602/0.444 |
| cg06677021 | 5 | 79490511 | SERINC5 |  | 0.2552/0.3403 |
| cg16518115 | 5 | 79549315 | SERINC5 |  | 0.3453/0.4329 |
| cg07019857 | 5 | 106822849 | EFNA5 |  | 0.72/0.6484 |
| cg09035699 | 5 | 131310461 | ACSL6 |  | 0.481/0.5885 |
| cg27024654 | 5 | 133904674 | PHF15 |  | 0.7285/0.6612 |
| cg12499311 | 5 | 134120499 | DDX46 |  | 0.6916/0.7467 |
| cg21069500 | 5 | 139050159 | CXXC5 |  | 0.532/0.4361 |
| cg08523384 | 5 | 141488047 | NDFIP1 |  | 0.2217/0.2886 |
| cg27579771 | 5 | 142431272 | ARHGAP26 |  | 0.6482/0.5364 |
| cg17969271 | 5 | 171430188 | FBXW11 |  | 0.457/0.3626 |
| cg06625767 | 5 | 176836695 | F12 |  | 0.7349/0.667 |
| cg13995774 | 5 | 179189810 | MAML1 |  | 0.7299/0.6621 |
| cg26166854 | 6 | 6614447 | LOC285780; LY86 |  | 0.2359/0.3349 |
| cg07970799 | 6 | 6614719 | LOC285780; LY86 |  | 0.3571/0.4582 |
| cg19988367 | 6 | 21808465 | FLJ22536 |  | 0.4903/0.5702 |
| cg00895196 | 6 | 22147182 | FLJ22536 |  | 0.6144/0.6753 |
| cg15019694 | 6 | 26217890 | HIST1H2BG |  | 0.0942/0.0656 |
| cg15878619 | 6 | 30687373 | TUBB |  | 0.1369/0.1046 |
| cg19279042 | 6 | 31550090 | LTB |  | 0.1272/0.166 |
| cg05554966 | 6 | 31648514 | LY6G5C |  | 0.1957/0.1688 |
| cg09993780 | 6 | 31648544 | LY6G5C |  | 0.1813/0.1544 |
| cg09027493 | 6 | 35109548 | TCP11 |  | 0.6607/0.7301 |
| cg12800266 | 6 | 37225002 | TMEM217; TBC1D22B |  | 0.7287/0.6732 |
| cg05255811 | 6 | 39192009 | KCNK5 |  | 0.6118/0.5121 |
| cg09130674 | 6 | 39195019 | KCNK5 |  | 0.4553/0.3812 |
| cg05568549 | 6 | 41907198 | CCND3 |  | 0.3539/0.2838 |
| cg27370104 | 6 | 42417938 | TRERF1 |  | 0.2962/0.3614 |
| cg06560379 | 6 | 44231305 | NFKBIE |  | 0.0558/0.0454 |
| cg24700316 | 6 | 90943562 | BACH2 |  | 0.5636/0.6452 |
| cg02836135 | 6 | 108052093 | SCML4 |  | 0.3833/0.4704 |
| cg17117243 | 6 | 109341365 | SESN1 |  | 0.2421/0.3343 |
| cg21775668 | 6 | 131147216 | LOC285733 |  | 0.4737/0.5633 |
| cg07474842 | 6 | 136915088 | MAP3K5 |  | 0.2696/0.2056 |
| cg15804973 | 6 | 137114513 | MAP3K5 |  | 0.4945/0.4268 |
| cg23280720 | 6 | 139483193 | HECA |  | 0.1766/0.2491 |
| cg16727231 | 6 | 139485336 | HECA |  | 0.353/0.439 |
| cg12603453 | 6 | 151694679 | ZBTB2 |  | 0.2985/0.4217 |
| cg07212702 | 6 | 157137791 | ARID1B |  | 0.6362/0.5565 |
| cg06012428 | 6 | 157477204 | ARID1B |  | 0.8452/0.7905 |
| cg08109681 | 6 | 166825084 | RPS6KA2 |  | 0.3544/0.4712 |
| cg24375364 | 7 | 2755000 | AMZ1 |  | 0.4579/0.5433 |
| cg13287553 | 7 | 4784419 | FOXK1 |  | 0.0945/0.1318 |
| cg02010481 | 7 | 28218524 | JAZF1 |  | 0.3199/0.2509 |
| cg14772935 | 7 | 29187019 | CPVL |  | 0.483/0.3996 |
| cg11251470 | 7 | 30008931 | SCRN1 |  | 0.5493/0.6403 |
| cg04858110 | 7 | 30009236 | SCRN1 |  | 0.4841/0.5646 |
| cg04065210 | 7 | 35074628 | DPY19L1 |  | 0.1506/0.1948 |
| cg03490567 | 7 | 43944817 | URGCP |  | 0.4688/0.5413 |
| cg18442362 | 7 | 44677772 | OGDH |  | 0.3066/0.2324 |
| cg07826859 | 7 | 45020086 | MYO1G |  | 0.3807/0.3053 |
| cg12573289 | 7 | 45075791 | CCM2 |  | 0.2313/0.2955 |
| cg26720010 | 7 | 45147131 | SNORA5B; TBRG4 |  | 0.412/0.5183 |
| cg11494773 | 7 | 48128242 | UPP1 |  | 0.1288/0.099 |
| cg25404758 | 7 | 70139561 | AUTS2 |  | 0.523/0.6331 |
| cg00907204 | 7 | 92461971 | CDK6 |  | 0.0869/0.0675 |
| cg21810604 | 7 | 99699701 | MCM7; AP4M1 |  | 0.0643/0.0512 |
| cg24616382 | 7 | 99767035 | GAL3ST4 |  | 0.4246/0.5228 |
| cg09507934 | 7 | 102072549 | ORAI2 |  | 0.154/0.2191 |
| cg25652701 | 7 | 105348517 | ATXN7L1 |  | 0.0611/0.0954 |
| cg06545367 | 7 | 110731527 | LRRN3; IMMP2L |  | 0.5296/0.6249 |
| cg24404329 | 7 | 115849899 | TES |  | 0.5715/0.6494 |
| cg12669355 | 7 | 116514552 | CAPZA2 |  | 0.3284/0.4238 |
| cg13914531 | 7 | 128579876 | IRF5 | IFN | 0.2876/0.2353 |
| cg05904013 | 7 | 128579933 | IRF5 | IFN | 0.5691/0.4931 |
| cg04864179 | 7 | 128579964 | IRF5 | IFN | 0.6984/0.6275 |
| cg11961845 | 7 | 129008179 | AHCYL2 |  | 0.3419/0.2849 |
| cg22544881 | 7 | 130712346 | FLJ43663 |  | 0.4147/0.3234 |
| cg16532400 | 7 | 150217056 | GIMAP7 |  | 0.3472/0.4612 |
| cg10777178 | 7 | 150264284 | GIMAP4 |  | 0.2817/0.3287 |
| cg13662290 | 7 | 150264311 | GIMAP4 |  | 0.1984/0.2521 |
| cg00323915 | 7 | 150264987 | GIMAP4 |  | 0.1942/0.2688 |
| cg16908215 | 7 | 150440016 | GIMAP5 |  | 0.4384/0.5361 |
| cg02704570 | 7 | 157647109 | PTPRN2 |  | 0.357/0.2891 |
| cg01462349 | 7 | 157664568 | PTPRN2 |  | 0.3259/0.4082 |
| cg17090611 | 8 | 17017866 | ZDHHC2 |  | 0.7815/0.6981 |
| cg03651021 | 8 | 19317380 | CSGALNACT1 |  | 0.5221/0.6215 |
| cg25764534 | 8 | 22485721 | BIN3 |  | 0.7408/0.6893 |
| cg09177577 | 8 | 22503577 | BIN3 |  | 0.6035/0.6976 |
| cg13580286 | 8 | 22925391 | TNFRSF10B |  | 0.1662/0.1351 |
| cg00420997 | 8 | 29607076 | C8orf75 |  | 0.1993/0.1551 |
| cg08573701 | 8 | 53603035 | RB1CC1 |  | 0.451/0.5525 |
| cg15973818 | 8 | 53623995 | RB1CC1 |  | 0.5691/0.6562 |
| cg14719959 | 8 | 61777711 | CHD7 |  | 0.4974/0.553 |
| cg25011252 | 8 | 61777859 | CHD7 |  | 0.4056/0.4738 |
| cg21750887 | 8 | 68658237 | CPA6 |  | 0.4091/0.4674 |
| cg19925215 | 8 | 80964918 | TPD52 |  | 0.4473/0.5191 |
| cg15723028 | 8 | 90776474 | RIPK2 |  | 0.5437/0.4242 |
| cg00219816 | 8 | 96280555 | C8orf37 |  | 0.7674/0.7071 |
| cg05127574 | 8 | 121714454 | SNTB1 |  | 0.5364/0.6467 |
| cg10054641 | 8 | 133773093 | TMEM71 |  | 0.1967/0.25 |
| cg23313885 | 8 | 133773115 | TMEM71 |  | 0.2891/0.3596 |
| cg17090901 | 8 | 133837475 | PHF20L1 |  | 0.5728/0.6555 |
| cg05037806 | 8 | 143407432 | TSNARE1 |  | 0.77/0.7163 |
| cg06517984 | 8 | 143407646 | TSNARE1 |  | 0.7953/0.7259 |
| cg02230964 | 8 | 143407817 | TSNARE1 |  | 0.7372/0.6701 |
| cg25875163 | 8 | 143763340 | PSCA |  | 0.8097/0.7664 |
| cg06894628 | 8 | 143822543 | SLURP1 |  | 0.5168/0.566 |
| cg21927363 | 9 | 15552206 | C9orf93 |  | 0.1058/0.0847 |
| cg21171339 | 9 | 79791169 | VPS13A |  | 0.4285/0.5013 |
| cg11516606 | 9 | 100175029 | TDRD7 |  | 0.0655/0.0487 |
| cg15551881 | 9 | 123688715 | TRAF1 |  | 0.3358/0.4358 |
| cg13696706 | 9 | 124396830 | DAB2IP |  | 0.2382/0.2765 |
| cg13473120 | 9 | 126776767 | LHX2 |  | 0.0265/0.0356 |
| cg13753351 | 9 | 127134207 | PSMB7 |  | 0.6685/0.5799 |
| cg14364797 | 9 | 132651576 | FNBP1 |  | 0.3484/0.4426 |
| cg10531986 | 9 | 132652466 | FNBP1 |  | 0.5186/0.6055 |
| cg06901890 | 9 | 132803508 | FNBP1 |  | 0.0768/0.0979 |
| cg14289429 | 9 | 134139878 | FAM78A |  | 0.4129/0.4868 |
| cg15986644 | 10 | 516683 | DIP2C |  | 0.5877/0.663 |
| cg14014799 | 10 | 22606053 | COMMD3 |  | 0.1719/0.117 |
| cg19378631 | 10 | 22606072 | COMMD3 |  | 0.1854/0.1243 |
| cg04691264 | 10 | 29697905 | LOC387647 |  | 0.6077/0.7176 |
| cg23648810 | 10 | 30337992 | KIAA1462 |  | 0.0975/0.1252 |
| cg04858631 | 10 | 74035570 | DDIT4 |  | 0.2262/0.3056 |
| cg08945443 | 10 | 75193254 | ZMYND17 |  | 0.3915/0.4998 |
| cg21398111 | 10 | 75528810 | SEC24C |  | 0.5341/0.6365 |
| cg25526001 | 10 | 85939451 | C10orf99 |  | 0.3913/0.4783 |
| cg16341836 | 10 | 90641389 | STAMBPL1 |  | 0.4572/0.3795 |
| cg23264429 | 10 | 90642003 | STAMBPL1 |  | 0.5293/0.3954 |
| cg14785527 | 10 | 90656978 | STAMBPL1 |  | 0.2858/0.2285 |
| cg15774510 | 10 | 90749966 | ACTA2; FAS |  | 0.2085/0.1505 |
| cg16257983 | 10 | 90750218 | ACTA2; FAS |  | 0.0721/0.0594 |
| cg27478224 | 10 | 91061115 | IFIT2 | IFN | 0.0786/0.0568 |
| cg03190891 | 10 | 97201172 | SORBS1 |  | 0.2033/0.2431 |
| cg15428620 | 10 | 102792835 | SFXN3 |  | 0.3253/0.2765 |
| cg26605164 | 10 | 102821565 | KAZALD1 |  | 0.5778/0.4883 |
| cg23691894 | 10 | 111765904 | ADD3 |  | 0.6534/0.7437 |
| cg03290131 | 10 | 112263831 | DUSP5 |  | 0.2869/0.188 |
| cg00291478 | 10 | 121301041 | RGS10 |  | 0.2597/0.3314 |
| cg05617307 | 10 | 121413182 | BAG3 |  | 0.5012/0.6217 |
| cg07858728 | 10 | 124319791 | DMBT1 |  | 0.1895/0.1304 |
| cg11524400 | 11 | 1778524 | CTSD; HCCA2 |  | 0.3384/0.4192 |
| cg08726522 | 11 | 8739587 | ST5 |  | 0.5151/0.4409 |
| cg15846482 | 11 | 18610557 | UEVLD |  | 0.326/0.2551 |
| cg23371436 | 11 | 20111534 | NAV2 |  | 0.3796/0.4498 |
| cg18908017 | 11 | 46353622 | DGKZ |  | 0.6017/0.656 |
| cg12027899 | 11 | 58385947 | ZFP91; ZFP91-CNTF |  | 0.6563/0.7106 |
| cg00271311 | 11 | 58389290 | CNTF; ZFP91-CNTF |  | 0.2979/0.3795 |
| cg14328641 | 11 | 59822727 | MS4A3 |  | 0.5628/0.4903 |
| cg03055440 | 11 | 59950405 | MS4A6A |  | 0.7789/0.6904 |
| cg24674703 | 11 | 60869960 | CD5 |  | 0.1669/0.242 |
| cg00299736 | 11 | 60869969 | CD5 |  | 0.0877/0.1216 |
| cg26904017 | 11 | 63634673 | MARK2 |  | 0.3545/0.4198 |
| cg24127061 | 11 | 65839402 | PACS1 |  | 0.3412/0.4261 |
| cg20234060 | 11 | 67183546 | ATPGD1 |  | 0.7084/0.7467 |
| cg23796243 | 11 | 67978654 | SUV420H1 |  | 0.285/0.3663 |
| cg08759026 | 11 | 69061454 | MYEOV |  | 0.3523/0.2961 |
| cg13505393 | 11 | 76377572 | LRRC32 |  | 0.3487/0.2928 |
| cg01475325 | 11 | 76498701 | TSKU |  | 0.517/0.5882 |
| cg02385173 | 11 | 76571534 | ACER3 |  | 0.5965/0.6792 |
| cg22335223 | 11 | 117698911 | FXYD2 |  | 0.3635/0.4623 |
| cg25155064 | 11 | 118100782 | MPZL3 |  | 0.3293/0.4505 |
| cg15350899 | 11 | 118781763 | BCL9L |  | 0.4327/0.5212 |
| cg20337103 | 11 | 118781778 | BCL9L |  | 0.2616/0.3431 |
| cg20029201 | 11 | 118781813 | BCL9L |  | 0.292/0.3985 |
| cg02341556 | 11 | 118781978 | BCL9L |  | 0.4729/0.5423 |
| cg12997404 | 11 | 118977474 | C2CD2L |  | 0.1675/0.1311 |
| cg24223075 | 11 | 119137279 | CBL |  | 0.2988/0.388 |
| cg09684429 | 11 | 124768015 | ROBO4 |  | 0.4248/0.3574 |
| cg03798942 | 11 | 128566958 | FLI1 |  | 0.2257/0.2982 |
| cg25354657 | 11 | 129991445 | APLP2 |  | 0.3674/0.4609 |
| cg00517080 | 11 | 134098583 | VPS26B |  | 0.6486/0.73 |
| cg08418872 | 12 | 6442954 | TNFRSF1A |  | 0.6642/0.7519 |
| cg23752651 | 12 | 6442966 | TNFRSF1A |  | 0.7694/0.8351 |
| cg22036538 | 12 | 6554051 | LOC678655; CD27 |  | 0.1489/0.2025 |
| cg00252813 | 12 | 6642229 | GAPDH |  | 0.2558/0.2021 |
| cg02519286 | 12 | 6642354 | GAPDH |  | 0.2808/0.2278 |
| cg09080114 | 12 | 6983111 | SPSB2 |  | 0.1588/0.1297 |
| cg15368872 | 12 | 10525233 | KLRK1 |  | 0.4576/0.3893 |
| cg01088404 | 12 | 48214523 | HDAC7 |  | 0.3429/0.2712 |
| cg18399183 | 12 | 51318138 | METTL7A |  | 0.2187/0.1784 |
| cg21253043 | 12 | 51783393 | GALNT6 |  | 0.5253/0.6165 |
| cg22193385 | 12 | 52638005 | KRT7 |  | 0.2153/0.1583 |
| cg22460123 | 12 | 52638294 | KRT7 |  | 0.3238/0.2791 |
| cg02699834 | 12 | 53039430 | KRT2 |  | 0.1149/0.1501 |
| cg02377704 | 12 | 53075359 | KRT1 |  | 0.2122/0.2965 |
| cg03348792 | 12 | 53075482 | KRT1 |  | 0.1625/0.1971 |
| cg11606261 | 12 | 53775336 | SP1 |  | 0.4156/0.3348 |
| cg08445469 | 12 | 53970794 | ATF7 |  | 0.5694/0.5159 |
| cg02710015 | 12 | 55362424 | KIAA0748 |  | 0.2572/0.3534 |
| cg24414325 | 12 | 56414442 | IKZF4 |  | 0.5134/0.4049 |
| cg00026033 | 12 | 56414490 | IKZF4 |  | 0.4807/0.4094 |
| cg20054248 | 12 | 56414508 | IKZF4 |  | 0.2574/0.2068 |
| cg01565774 | 12 | 56414533 | IKZF4 |  | 0.2149/0.1664 |
| cg06015525 | 12 | 57872123 | ARHGAP9 |  | 0.2356/0.2979 |
| cg15016701 | 12 | 63211683 | PPM1H |  | 0.1003/0.1227 |
| cg27292835 | 12 | 65063890 | RASSF3 |  | 0.0835/0.1091 |
| cg02266731 | 12 | 69357333 | CPM |  | 0.4036/0.3172 |
| cg25886621 | 12 | 93130251 | PLEKHG7 |  | 0.4987/0.5909 |
| cg16871561 | 12 | 98986887 | SLC25A3 |  | 0.324/0.4269 |
| cg25684349 | 12 | 107725084 | BTBD11 |  | 0.2032/0.2499 |
| cg10421247 | 12 | 120524653 | CCDC64 |  | 0.3597/0.2969 |
| cg16460342 | 12 | 121662577 | P2RX4 |  | 0.1216/0.1693 |
| cg06152215 | 12 | 124422259 | CCDC92 |  | 0.063/0.0839 |
| cg01230386 | 12 | 133383619 | GOLGA3 |  | 0.3839/0.5071 |
| cg09120938 | 12 | 133424655 | CHFR |  | 0.7873/0.8435 |
| cg17524886 | 12 | 133424709 | CHFR |  | 0.6614/0.7308 |
| cg18332814 | 13 | 24247649 | TNFRSF19 |  | 0.7625/0.8288 |
| cg01373189 | 13 | 33002820 | N4BP2L1 |  | 0.4002/0.344 |
| cg07880943 | 13 | 46744500 | LCP1 |  | 0.3907/0.4623 |
| cg02043329 | 13 | 49740771 | FNDC3A |  | 0.7756/0.7 |
| cg12229775 | 13 | 74315385 | KLF12 |  | 0.4259/0.5232 |
| cg17799287 | 13 | 92001764 | MIR19A; MIR18A; MIR17HG; MIR17 |  | 0.1754/0.128 |
| cg23665802 | 13 | 92002338 | MIR92A1; MIR19A; MIR18A; MIR19B1; MIR17HG; MIR17; MIR20A |  | 0.4281/0.3484 |
| cg16131748 | 13 | 99959606 | UBAC2; GPR183 |  | 0.0455/0.0633 |
| cg18560638 | 13 | 100008200 | MIR623; UBAC2 |  | 0.3741/0.4609 |
| cg07218880 | 13 | 115046279 | UPF3A |  | 0.2969/0.4043 |
| cg25268718 | 14 | 24604711 | PSME1 |  | 0.5934/0.5401 |
| cg21052932 | 14 | 51342320 | ABHD12B |  | 0.1696/0.2511 |
| cg00739471 | 14 | 69415588 | ACTN1 |  | 0.469/0.5774 |
| cg08280368 | 14 | 71110536 | TTC9 |  | 0.3776/0.5024 |
| cg26217402 | 14 | 74238381 | C14orf43 |  | 0.4115/0.4883 |
| cg13027206 | 14 | 91866325 | CCDC88C |  | 0.1167/0.1494 |
| cg19903805 | 14 | 92333771 | TC2N |  | 0.2059/0.2762 |
| cg24130561 | 14 | 95621734 | DICER1 |  | 0.5188/0.5988 |
| cg16062483 | 14 | 98444417 | C14orf64 |  | 0.3289/0.4246 |
| cg16278496 | 14 | 98444476 | C14orf64 |  | 0.2706/0.35 |
| cg08217526 | 14 | 98445245 | C14orf64 |  | 0.1945/0.2454 |
| cg10178917 | 14 | 99665210 | BCL11B |  | 0.479/0.5432 |
| cg02963266 | 14 | 99681710 | BCL11B |  | 0.3416/0.4474 |
| cg23479730 | 14 | 99681757 | BCL11B |  | 0.5329/0.6235 |
| cg04166500 | 14 | 100571607 | EVL |  | 0.4442/0.5161 |
| cg10167235 | 14 | 100807646 | WARS |  | 0.3785/0.3112 |
| cg00955451 | 15 | 29213640 | APBA2 |  | 0.5486/0.6174 |
| cg10768063 | 15 | 29213736 | APBA2 |  | 0.4854/0.565 |
| cg19847577 | 15 | 29213748 | APBA2 |  | 0.2236/0.2771 |
| cg12044210 | 15 | 29213858 | APBA2 |  | 0.6619/0.7385 |
| cg21917349 | 15 | 29213860 | APBA2 |  | 0.4611/0.5547 |
| cg11098259 | 15 | 58430391 | AQP9 |  | 0.5987/0.4968 |
| cg11200462 | 15 | 66786368 | SNAPC5 |  | 0.778/0.7007 |
| cg20055861 | 15 | 68055293 | MAP2K5 |  | 0.2773/0.3595 |
| cg15188939 | 15 | 72809154 | ARIH1 |  | 0.5926/0.6644 |
| cg02329430 | 15 | 73921385 | NPTN |  | 0.4282/0.4938 |
| cg02489956 | 15 | 81282510 | MESDC2 |  | 0.0799/0.066 |
| cg23536830 | 15 | 91162876 | CRTC3 |  | 0.6908/0.7597 |
| cg19348484 | 15 | 91413236 | FURIN |  | 0.2714/0.2043 |
| cg01042641 | 16 | 1575979 | IFT140 |  | 0.3331/0.4139 |
| cg01994902 | 16 | 1576069 | IFT140 |  | 0.2653/0.3141 |
| cg27316811 | 16 | 1576146 | IFT140 |  | 0.6394/0.7687 |
| cg10471113 | 16 | 1587842 | TMEM204; IFT140 |  | 0.3469/0.4356 |
| cg00305585 | 16 | 1610898 | IFT140 |  | 0.4264/0.5253 |
| cg06965409 | 16 | 1611973 | IFT140 |  | 0.8571/0.903 |
| cg08961793 | 16 | 28628118 | SULT1A1 |  | 0.4983/0.4273 |
| cg06453916 | 16 | 29690524 | QPRT |  | 0.3785/0.3192 |
| cg07046436 | 16 | 30663252 | PRR14 |  | 0.1404/0.1166 |
| cg10421029 | 16 | 30936028 | NCRNA00095; FBXL19 |  | 0.3409/0.2873 |
| cg10045909 | 16 | 31075842 | ZNF668 |  | 0.1411/0.19 |
| cg11884546 | 16 | 31366377 | ITGAX |  | 0.104/0.0773 |
| cg04742550 | 16 | 31366429 | ITGAX |  | 0.2922/0.2237 |
| cg16750777 | 16 | 50583441 | NKD1 |  | 0.6248/0.682 |
| cg01981760 | 16 | 53737576 | RPGRIP1L; FTO |  | 0.1334/0.1127 |
| cg08958168 | 16 | 68001415 | SLC12A4 |  | 0.4371/0.4983 |
| cg00259097 | 16 | 70770604 | VAC14 |  | 0.5873/0.6667 |
| cg08329113 | 16 | 70771142 | VAC14 |  | 0.5595/0.6342 |
| cg07846061 | 16 | 74732434 | MLKL |  | 0.4457/0.3532 |
| cg15286847 | 16 | 84690433 | KLHL36 |  | 0.3455/0.4349 |
| cg04431002 | 16 | 84766438 | USP10 |  | 0.86/0.7992 |
| cg03776194 | 16 | 88770966 | RNF166 |  | 0.2827/0.3617 |
| cg08843248 | 16 | 89009929 | CBFA2T3 |  | 0.408/0.4569 |
| cg06755448 | 16 | 89791093 | ZNF276 |  | 0.6481/0.7372 |
| cg03809021 | 16 | 89831123 | FANCA |  | 0.5974/0.5061 |
| cg15380836 | 17 | 1553341 | RILP |  | 0.1532/0.1247 |
| cg12077963 | 17 | 4079306 | ANKFY1 |  | 0.3816/0.4797 |
| cg13221924 | 17 | 6495080 | KIAA0753 |  | 0.7202/0.6405 |
| cg14018648 | 17 | 7083015 | ASGR1 |  | 0.6067/0.5252 |
| cg03613649 | 17 | 7341191 | FGF11 |  | 0.7998/0.7648 |
| cg12699156 | 17 | 7517016 | SHBG; FXR2 |  | 0.1763/0.221 |
| cg00688810 | 17 | 7517138 | SHBG; FXR2 |  | 0.4455/0.5208 |
| cg01046511 | 17 | 7742971 | KDM6B |  | 0.847/0.7999 |
| cg04658021 | 17 | 8056967 | PER1 |  | 0.3024/0.2332 |
| cg08461692 | 17 | 8481454 | MYH10 |  | 0.1953/0.2493 |
| cg21554670 | 17 | 9967417 | GAS7 |  | 0.5819/0.5006 |
| cg08036492 | 17 | 13976536 | COX10 |  | 0.7064/0.6015 |
| cg12370935 | 17 | 16976475 | MPRIP |  | 0.3877/0.4542 |
| cg05105919 | 17 | 25958673 | LGALS9 |  | 0.0909/0.0744 |
| cg03909504 | 17 | 25959847 | LGALS9 |  | 0.3506/0.3033 |
| cg19001909 | 17 | 26205940 | C17orf108 |  | 0.524/0.6236 |
| cg01792117 | 17 | 27088227 | C17orf63 |  | 0.8215/0.7747 |
| cg19048010 | 17 | 28084996 | SSH2 |  | 0.3637/0.4655 |
| cg16563370 | 17 | 33775952 | SLFN13 |  | 0.5615/0.4662 |
| cg07660627 | 17 | 35481970 | ACACA |  | 0.5887/0.6718 |
| cg06684503 | 17 | 36873584 | MLLT6 |  | 0.572/0.6477 |
| cg18984002 | 17 | 36876514 | MLLT6 |  | 0.7653/0.8134 |
| cg23901967 | 17 | 36890321 | CISD3; PCGF2 |  | 0.3847/0.4577 |
| cg10430963 | 17 | 37124558 | FBXO47 |  | 0.6589/0.7494 |
| cg03293732 | 17 | 38017814 | IKZF3 |  | 0.644/0.7439 |
| cg00442282 | 17 | 38471064 | RARA |  | 0.1442/0.1184 |
| cg11094248 | 17 | 38494580 | RARA |  | 0.1161/0.0892 |
| cg17980404 | 17 | 38601676 | IGFBP4 |  | 0.4503/0.3595 |
| cg14039779 | 17 | 41857714 | C17orf105; DUSP3 |  | 0.6319/0.5528 |
| cg00260201 | 17 | 46029581 | PRR15L |  | 0.3443/0.2689 |
| cg27050612 | 17 | 46133198 | NFE2L1 |  | 0.3898/0.3355 |
| cg17839611 | 17 | 47286802 | GNGT2; ABI3 |  | 0.439/0.5173 |
| cg02275530 | 17 | 59328313 | BCAS3 |  | 0.7276/0.6421 |
| cg09121543 | 17 | 61774794 | LIMD2 |  | 0.2974/0.3584 |
| cg25061701 | 17 | 62608856 | SMURF2 |  | 0.6572/0.7349 |
| cg26921093 | 17 | 63534688 | AXIN2 |  | 0.4278/0.5411 |
| cg06559756 | 17 | 65464297 | PITPNC1 |  | 0.4177/0.509 |
| cg08434692 | 17 | 73086066 | SLC16A5 |  | 0.6774/0.7628 |
| cg26550194 | 17 | 74639928 | ST6GALNAC1 |  | 0.1037/0.1393 |
| cg19950606 | 17 | 76121276 | TMC6 |  | 0.4512/0.5202 |
| cg07313882 | 17 | 76121348 | TMC6 |  | 0.1957/0.2405 |
| cg26003388 | 17 | 76129533 | TMC8; TMC6 |  | 0.6704/0.7455 |
| cg22833809 | 17 | 76129984 | TMC8; TMC6 |  | 0.3143/0.3687 |
| cg07121312 | 17 | 77970193 | TBC1D16 |  | 0.1262/0.1683 |
| cg16541275 | 17 | 78821754 | RPTOR |  | 0.3996/0.4451 |
| cg16124975 | 17 | 79938941 | ASPSCR1 |  | 0.0333/0.0429 |
| cg12182124 | 18 | 21451563 | LAMA3 |  | 0.1244/0.1565 |
| cg20937934 | 18 | 21452788 | LAMA3 |  | 0.6301/0.7041 |
| cg13270625 | 18 | 21452819 | LAMA3 |  | 0.4768/0.5526 |
| cg03634729 | 18 | 21452829 | LAMA3 |  | 0.373/0.4418 |
| cg01152726 | 18 | 21452844 | LAMA3 |  | 0.5839/0.6782 |
| cg26485825 | 18 | 21452895 | LAMA3 |  | 0.4301/0.5376 |
| cg14663914 | 19 | 827739 | AZU1 |  | 0.5/0.4306 |
| cg02324006 | 19 | 1080034 | HMHA1 |  | 0.5704/0.6524 |
| cg03882382 | 19 | 4540065 | LRG1 |  | 0.2998/0.2435 |
| cg17714703 | 19 | 4912221 | UHRF1 |  | 0.0764/0.1206 |
| cg24876035 | 19 | 7682769 | KIAA1543 |  | 0.956/0.9338 |
| cg04334723 | 19 | 13054427 | CALR |  | 0.5882/0.5191 |
| cg27384695 | 19 | 19271486 | LOC729991-MEF2B; MEF2B |  | 0.174/0.2209 |
| cg12103219 | 19 | 30165308 | PLEKHF1 |  | 0.8432/0.908 |
| cg19977428 | 19 | 35819985 | CD22 |  | 0.28/0.2221 |
| cg25289028 | 19 | 36428519 | LRFN3 |  | 0.4198/0.5411 |
| cg17769442 | 19 | 45578863 | ZNF296 |  | 0.1285/0.1014 |
| cg00763834 | 19 | 45927335 | ERCC1 |  | 0.056/0.0475 |
| cg22810489 | 19 | 45927585 | ERCC1 |  | 0.3302/0.2773 |
| cg05492306 | 19 | 45927594 | ERCC1 |  | 0.3919/0.3232 |
| cg18734095 | 19 | 50062005 | NOSIP |  | 0.2576/0.3521 |
| cg22820233 | 19 | 55385581 | FCAR |  | 0.674/0.5586 |
| cg09592958 | 20 | 207161 | DEFB129 |  | 0.4291/0.5367 |
| cg22052056 | 20 | 31351813 | DNMT3B |  | 0.7551/0.6711 |
| cg26347170 | 20 | 32441424 | CHMP4B |  | 0.4102/0.5183 |
| cg00732815 | 20 | 44636981 | MMP9 |  | 0.1482/0.1137 |
| cg06122230 | 20 | 47887219 | ZNFX1 |  | 0.7508/0.6626 |
| cg02828104 | 20 | 48770800 | TMEM189; TMEM189-UBE2V1 |  | 0.359/0.4259 |
| cg12831034 | 20 | 57582971 | CTSZ |  | 0.1993/0.1609 |
| cg14977069 | 20 | 62367698 | LIME1 |  | 0.186/0.2572 |
| cg06653796 | 20 | 62367805 | LIME1 |  | 0.0865/0.1318 |
| cg00446123 | 20 | 62367888 | LIME1 |  | 0.2091/0.3022 |
| cg13277040 | 20 | 62716332 | OPRL1; C20orf201 |  | 0.4723/0.4422 |
| cg21291385 | 21 | 35448215 | SLC5A3; MRPS6 |  | 0.1642/0.2207 |
| cg15683970 | 21 | 35747081 | FAM165B |  | 0.4197/0.3508 |
| cg21258596 | 21 | 38338458 | HLCS |  | 0.1382/0.1121 |
| cg19788186 | 21 | 39669558 | KCNJ15 |  | 0.5326/0.6342 |
| cg01881899 | 21 | 43652704 | ABCG1 |  | 0.0885/0.1171 |
| cg23732182 | 21 | 44898090 | C21orf84 |  | 0.6416/0.5781 |
| cg16334524 | 21 | 44898123 | C21orf84 |  | 0.6545/0.588 |
| cg21913632 | 21 | 46505130 | ADARB1 |  | 0.32/0.4035 |
| cg22517527 | 21 | 48056967 | PRMT2 |  | 0.3686/0.4859 |
| cg26354221 | 22 | 24822802 | ADORA2A |  | 0.7859/0.8439 |
| cg26001125 | 22 | 24823050 | ADORA2A |  | 0.4724/0.5462 |
| cg15499799 | 22 | 24823110 | ADORA2A |  | 0.604/0.6753 |
| cg04250930 | 22 | 24823141 | ADORA2A |  | 0.6857/0.7455 |
| cg04990420 | 22 | 24824362 | ADORA2A |  | 0.2283/0.2961 |
| cg23072383 | 22 | 31031044 | SLC35E4 |  | 0.2153/0.1624 |
| cg23825480 | 22 | 31336785 | MORC2 |  | 0.2052/0.3007 |
| cg08612539 | 22 | 37257124 | NCF4 |  | 0.2024/0.1536 |
| cg21345826 | 22 | 39353650 | APOBEC3A |  | 0.7028/0.6202 |
| cg03318904 | 22 | 39801522 | MAP3K7IP1 |  | 0.5134/0.5955 |
| cg26815454 | 22 | 40296767 | GRAP2 |  | 0.1336/0.1798 |
| cg04136484 | 22 | 40589892 | TNRC6B |  | 0.3346/0.3726 |
| cg04630823 | 22 | 45575276 | NUP50 |  | 0.1868/0.2417 |

Listed are those genes with a highly significant (p <1 x10^-8^) SLE-associated methylation difference that is unique to one cell-type.
