# Supplementary material for: Genome-Wide DNA Methylation Analysis of Systemic Lupus Erythematosus Reveals Persistent Hypomethylation of Interferon Genes and Compositional Changes to CD4+ T-cell Populations
Source: PLoS Genet. 2013 Aug 8;9(8):e1003678. doi: 10.1371/journal.pgen.1003678 (PMC3738443; doi:10.1371/journal.pgen.1003678)
Supplement: Table S6 — CpGs showing altered methylation on the X-chromosome in females. Listed are the top CpGs from the disease association tests on the X-chromosome in females. CpGs with FDR<1% are listed. The rightmost column indicates mean methylation proportion after correction for all covariates in controls/SLE patients. (DOCX) [file pgen.1003678.s010.docx]

**Table S6. CpGs Showing Altered Methylation on the X-chromosome in Females.**

|  |  |  |  |  |  |  |
| --- | --- | --- | --- | --- | --- | --- |
| **CpG** | **Chr** | **Pos** | **Genes** | **IFN** | **P-value** | **CD4** |
| cg15241084 | X | 12886121 | TLR7 | IFN | 2.62E-08 | 0.7466/0.7078 |
| cg24735671 | X | 12883760 | TLR7 | IFN | 8.06E-08 | 0.8201/0.7852 |
| cg02033323 | X | 49118313 | FOXP3 |  | 1.08E-07 | 0.8873/0.8629 |
| cg22695906 | X | 133955535 | FAM122C |  | 2.68E-07 | 0.6832/0.6305 |
| cg04710661 | X | 2848381 | ARSD |  | 5.01E-07 | 0.8025/0.7621 |
| cg17959821 | X | 53311012 | IQSEC2 |  | 5.43E-07 | 0.7761/0.7405 |
| cg00422452 | X | 128872061 | XPNPEP2 |  | 8.03E-07 | 0.7411/0.6968 |
| cg07885785 | X | 30591068 | CXorf21 |  | 8.69E-07 | 0.7025/0.6556 |
| cg17207552 | X | 39767775 |  |  | 1.02E-06 | 0.5145/0.4697 |
| cg01336098 | X | 21901558 | MBTPS2 |  | 1.15E-06 | 0.855/0.8307 |
| cg07343739 | X | 46617524 | SLC9A7 |  | 1.16E-06 | 0.6571/0.621 |
| cg24618413 | X | 40007471 | BCOR |  | 1.39E-06 | 0.4401/0.4078 |
| cg15812025 | X | 153523509 | TKTL1; TEX28 |  | 1.88E-06 | 0.835/0.811 |
| cg24643262 | X | 15518862 | BMX |  | 2.26E-06 | 0.712/0.6723 |
| cg05393376 | X | 53467938 |  |  | 2.38E-06 | 0.213/0.1734 |
| cg06219927 | X | 53310660 | IQSEC2 |  | 2.61E-06 | 0.7353/0.7017 |
| cg26803301 | X | 153523498 | TKTL1; TEX28 |  | 2.83E-06 | 0.8898/0.8681 |
| cg17734525 | X | 48592880 |  |  | 3.02E-06 | 0.7048/0.6718 |
| cg00719224 | X | 133681567 | MIR503; MGC16121; MIR424 |  | 3.06E-06 | 0.7865/0.7568 |
| cg06701191 | X | 65238507 | MIR223 |  | 3.06E-06 | 0.4601/0.4179 |
| cg22666517 | X | 9916563 | SHROOM2 |  | 5.60E-06 | 0.6771/0.7083 |
| cg27360158 | X | 41133058 |  |  | 5.87E-06 | 0.743/0.7021 |
| cg21283832 | X | 152680754 |  |  | 6.09E-06 | 0.7772/0.7397 |
| cg18433694 | X | 138914571 | ATP11C |  | 6.25E-06 | 0.7889/0.7538 |
| cg15383633 | X | 21674175 | KLHL34 |  | 6.46E-06 | 0.6749/0.6371 |
| cg13716034 | X | 65238737 | MIR223 |  | 7.40E-06 | 0.7011/0.6438 |
| cg00854925 | X | 152689318 |  |  | 9.01E-06 | 0.7996/0.7706 |
| cg10607675 | X | 21674415 | KLHL34 |  | 1.00E-05 | 0.5586/0.5292 |
| cg21330641 | X | 118780930 | SEPT6; MIR766 |  | 1.19E-05 | 0.8373/0.8181 |
| cg10913227 | X | 53469182 |  |  | 1.30E-05 | 0.4453/0.4137 |
| cg19912147 | X | 133955434 | FAM122C |  | 1.38E-05 | 0.627/0.5931 |
| cg17281810 | X | 40007949 | BCOR |  | 1.41E-05 | 0.4132/0.3634 |
| cg22903370 | X | 153058860 | IDH3G; SSR4 |  | 1.43E-05 | 0.6278/0.599 |
| cg08065271 | X | 153090978 | PDZD4 |  | 1.55E-05 | 0.607/0.5745 |
| cg13310805 | X | 35937904 | CXorf22 |  | 2.15E-05 | 0.4001/0.3659 |
| cg02183297 | X | 108867379 | KCNE1L |  | 2.86E-05 | 0.7864/0.7586 |
| cg08630881 | X | 70713213 | INGX; BCYRN1 |  | 2.86E-05 | 0.819/0.7836 |
| cg01115636 | X | 119751976 | MCTS1 |  | 2.93E-05 | 0.8754/0.8516 |
| cg12124912 | X | 65259910 | VSIG4 |  | 2.97E-05 | 0.6981/0.6448 |
| cg03758972 | X | 101964596 |  |  | 3.18E-05 | 0.5621/0.6267 |
| cg15068728 | X | 67913143 | STARD8 |  | 3.33E-05 | 0.8923/0.8782 |
| cg20959965 | X | 37606012 |  |  | 3.55E-05 | 0.5875/0.5573 |
| cg13015245 | X | 130207604 | FLJ30058 |  | 3.62E-05 | 0.2895/0.2657 |

Listed are the top CpGs from the disease association tests on the X-chromosome in females. CpGs with FDR<1% are listed. The rightmost column indicates mean methylation proportion after correction for all covariates in controls/SLE patients.
