# Supplementary material for: Genome-Wide DNA Methylation Analysis of Systemic Lupus Erythematosus Reveals Persistent Hypomethylation of Interferon Genes and Compositional Changes to CD4+ T-cell Populations
Source: PLoS Genet. 2013 Aug 8;9(8):e1003678. doi: 10.1371/journal.pgen.1003678 (PMC3738443; doi:10.1371/journal.pgen.1003678)
Supplement: Table S7 — Functional analysis of significant CpGs in three cell types. Results from Ingenuity Pathway Analysis for the highly significant CpGs in each cell type and the mildly significant CpGs in T-cells. (DOCX) [file pgen.1003678.s011.docx]

**Table S7.** **Functional Analysis of Significant CpGs in Three Cell Types.**

| **IPA Canonical Pathways** |  |
| --- | --- |
| **Genes near top 100 CD4 CpGs** | **p-value** |
| Interferon signaling | 2.06E-06 |
| Pattern recognition receptors of bacteria and viruses | 1.30E-04 |
| Retinoic acid mediated apoptosis signaling | 6.90E-04 |
| **Genes near top 100 CD14 CpGs** | **p-value** |
| Interferon signaling | 4.53E-14 |
| Antigen presentation pathway | 9.57E-14 |
| Protein ubiquination pathway | 7.98E-06 |
| **Genes near top 100 CD19 CpGs** | **p-value** |
| Interferon signalling | 6.31E-12 |
| JAK family kinases in IL-6 signaling | 5.31E-05 |
| Oncostatin M signaling | 1.35E-04 |
| **Genes near CD4 CpGs with 1E-08>P>1E-11** | **p-value** |
| p38 MAPK signaling | 1.17E-04 |
|  |  |
| **IPA Upstream Regulators** |  |
| **Genes near top 100 CD4 CpGs** | **p-value** |
| IFNA2 | 3.42E-30 |
| IFNL1 | 7.91E-30 |
| NKX2-3 | 6.51E-20 |
| **Genes near top 100 CD14 CpGs** | **p-value** |
| IFNA2 | 1.54E-43 |
| IFNL1 | 2.35E-36 |
| MAPK1 | 6.47E-28 |
| **Genes near top 100 CD19 CpGs** | **p-value** |
| IFNL1 | 2.32E-43 |
| IFNA2 | 3.77E-41 |
| IRF7 | 6.86E-25 |
| **Genes near CD4 CpGs with 1E-08>P>1E-11** | **p-value** |
| IFNA2 | 1.35E-12 |
| miR-421-3p | 6.94E-10 |
| IFNL1 | 8.18E-10 |

Results from Ingenuity Pathway Analysis for the highly significant CpGs in each cell type and the mildly significant CpGs in T-cells.
